# Traditional Chinese Medicine for Neck Pain and Low Back Pain: A Systematic Review and Meta-Analysis

**DOI:** 10.1371/journal.pone.0117146

**Published:** 2015-02-24

**Authors:** Qi-ling Yuan, Tuan-mao Guo, Liang Liu, Fu Sun, Yin-gang Zhang

**Affiliations:** 1 Department of Orthopaedics of the First Affiliated Hospital, Medical School, Xi’an Jiaotong University, Xi’an 710061, China; 2 Second department of Orthopaedics, Xianyang Central Hospital, Xianyang, Shaanxi, P.R. 712000, China; 3 Department of Orthopaedics of the Affiliated Hospital of Xi’an Medical College, Xi’an 710077, China; Stavanger University Hospital, NORWAY

## Abstract

**Background:**

Neck pain (NP) and low back pain (LBP) are common symptoms bothering people in daily life. Traditional Chinese medicine (TCM) has been used to treat various symptoms and diseases in China and has been demonstrated to be effective. The objective of the present study was to review and analyze the existing data about pain and disability in TCM treatments for NP and LBP.

**Methods:**

Studies were identified by a comprehensive search of databases, such as MEDLINE, EMBASE, and Cochrane Library, up to September 1, 2013. A meta-analysis was performed to evaluate the efficacy and safety of TCM in managing NP and LBP.

**Results:**

Seventy five randomized controlled trials (n = 11077) were included. Almost all of the studies investigated individuals experiencing chronic NP (CNP) or chronic LBP (CLBP). We found moderate evidence that acupuncture was more effective than sham-acupuncture in reducing pain immediately post-treatment for CNP (visual analogue scale (VAS) 10 cm, mean difference (MD) = -0.58 (-0.94, -0.22), 95% confidence interval, p = 0.01), CLBP (standardized mean difference = -0.47 (-0.77, -0.17), p = 0.003), and acute LBP (VAS 10 cm, MD = -0.99 (-1.24, -0.73), p< 0.001). Cupping could be more effective than waitlist in VAS (100 mm) (MD = -19.10 (-27.61, -10.58), p < 0. 001) for CNP or medications (e.g. NSAID) for CLBP (MD = -5.4 (-8.9, -0.19), p = 0.003). No serious or life-threatening adverse effects were found.

**Conclusions:**

Acupuncture, acupressure, and cupping could be efficacious in treating the pain and disability associated with CNP or CLBP in the immediate term. Gua sha, tai chi, qigong, and Chinese manipulation showed fair effects, but we were unable to draw any definite conclusions, and further research is still needed. The efficacy of tuina and moxibustion is unknown because no direct evidence was obtained. These TCM modalities are relatively safe.

## Introduction

Neck pain (NP) and low back pain (LBP) are common symptoms bothering people in daily life. In developed countries, more than 70% of people experience LBP [1], whereas approximately two-thirds of people experience NP [2,3] at some point in their lifetimes. These two disorders occur most frequently among the middle-aged population, in which the proportion of females is higher than that of males [4]. A majority of the acute NP and LBP sufferers obtain spontaneous relief within days or weeks, although approximately 10% of acute NP [2] and 20% of acute LBP [5] suffers experience the condition as chronic or persistent. NP and LBP can be caused by specific pathological diseases, such as tumors, infection, fracture, and inflammation. However, the pain in most individuals (approximately 85% for LBP) [1] is non-specific, which indicates that the pain is not attributable to one of the definite above pathologies but instead to some ambiguous etiology. Conventional treatments, such as medications [6] and surgery [7], have demonstrated some efficacy. Nonetheless, these treatments were not always effective, and even had some serious adverse effects [7,8]. Consequently, to find some more effective therapeutic methods, many individuals have turned their attention to some other treatments, such as complementary and alternative medicine (CAM). Although CAM may also have some adverse effects of itself, given the numerous therapeutic methods of CAM and its positive effectiveness to some extent, a growing number of researchers have focused on various CAM therapies, such as acupuncture, massage, exercise, and hydrotherapy [9]. Most significantly, as a type of traditional Chinese medicine (TCM), acupuncture has shown respectable efficacy and is broadly accepted internationally [10–12].

TCM is based on the fundamental theory of balance among yin and yang, five basic elements, and a relationship between humans and nature [13,14]. TCM has been used to treat various diseases in China and even throughout East Asia for more than 2000 years, and it still remains the first choice of treatment for many people. However, the different models of thought that are the foundations of TCM and modern science are not compatible, hindering the spread of TCM worldwide. Nevertheless, there are many articles published in various scientific journals that have attempted to explain some phenomena and mechanisms of treatments in TCM from the perspective of modern medicine.

Although acupuncture is a typical representative of TCM, it is only one of the various general therapies for NP and LBP, such as acupressure, cupping, moxibustion, tuina, gua sha, tai chi, qigong, Chinese herbal medicine, and Chinese manipulation (for definitions, see S1 Table). The objective of the present study was to review and analyze the existing data about pain and disability associated with TCM treatments for NP and LBP. The question of our study is “whether TCM treatments are more effective in pain relief or disability improvement as compared with other treatments for people with NP or LBP?”.

## Methods

### Data Sources and Search Strategy

Studies were identified by a comprehensive search in the following databases: MEDLINE, EMBASE, the Cochrane Library and the Traditional Chinese Medical Literature Analysis and Retrieval System (TCMLARS) and China National Knowledge Infrastructure (CNKI) and the Wan Fang database. The search was conducted between the inception of each database and September 1, 2013, and updated on May 25, 2014 using disease-specific search string combinations, partly according to the strategy outlined by the Cochrane Back Review Group (CBRG) (S2 Table), with subject limitations within the English or Chinese language. There were no restrictions about publication status of the searched trials. The reference lists of identified studies were screened manually for more studies related. Experts in the representative fields were also contacted for unpublished trials. The search was conducted by a veteran librarian.

### Study Selection Criteria

We included any randomized controlled trial (RCT) meeting all of the following criteria: (1) the work is published in the English or Chinese language; (2) the subjects included are men or women (age ≥17 years) with NP or LBP (with or without radiating pain) of any duration; (3) at least one of the therapies assessed pertains to TCM; (4) a comparison should be done between TCM and other treatment (e.g. TCM versus other treatment, TCM versus no treatment, TCM plus other treatment versus other treatment); (5) at least one of the following outcomes was evaluated: pain intensity or disability; (6) the principle summary measures should better be commonly used, such as pain intensity (e.g., visual analogue scale, VAS; numerical rating scale, NRS) and disability (e.g., Oswestry Disability Index, ODI; Neck Disability Index, NDI); (7) the duration of follow-up should be at least one day after all treatment sessions were concluded according to the study design of each corresponding trial.

We excluded trials of neck or back pain caused by trauma, infection, cauda equina syndrome, bone rarefaction, compression fracture of a vertebral body, tumor, or fibromyalgia.

### Data Extraction

Two evaluators independently extracted the data from the studies or SRs, and discrepancies were resolved by negotiation or a third party.

The duration of pain was defined as follows: (1) chronic (≥ 3 months); (2) sub-acute (~1–3 months); and (3) acute (< 1 month). In contrast, the follow-up (post-intervention) times were defined as follows: (1) immediate term (≤ 1 week); (2) short term (≤ 3 months); (3) intermediate term (~3–12 months); and (4) long term (≥ 1 year).

Primary outcomes included pain intensity (e.g., visual analogue scale, VAS; numerical rating scale, NRS) and disability (e.g., Oswestry Disability Index, ODI 0–60 points). Additionally, side effects (including the names of adverse effects and the number or proportion of individuals experiencing them) were recorded.

The study, treatment, population, and outcome characteristics are summarized in tables.

### Assessment of Study Quality and Reporting

Two independent assessors evaluated the quality of every trial included in our review. Discrepancies were resolved by negotiation or an authoritative third party.

The quality of the individual trials was rated according to the criteria of the Cochrane Back Review Group (Table 1) [15]. Depending on the number of “Yes” responses (coded as “1”) for four particular items (e.g., allocation concealment, baseline similarity, patient blinding, and number of or reason for dropouts), yielding a range of scores from 0–4, the quality of individual trials was graded according to the following three ranks: good (score = 4), fair (score = 2–3), or poor (score = 0–1). Because the number of “Yes” responses was recorded for 4 domains, in case of a single study, N was a whole number (0, 1, 2, 3, or 4); in case of multiple studies, N was the average number, which may have been either a whole number or a fraction (S3 Table).

**Table 1:** Updated Method Guidelines for Systematic Reviews in the Cochrane Collaboration Back Review Group—a 12 Item Tool.

Question	Item	Rating
**Q1**	Was the method of randomization adequate?	Yes / No / Unsure
**Q2**	Was the treatment allocation concealed?	Yes / No / Unsure
**Q3**	Were the groups similar at baseline regarding the most important prognostic indicators?	Yes / No / Unsure
**Q4**	Was the patient blinded to the intervention?	Yes / No / Unsure
**Q5**	Was the care provider blinded to the intervention?	Yes / No / Unsure
**Q6**	Was the outcome assessor blinded to the intervention?	Yes / No / Unsure
**Q7**	Were co-interventions avoided or similar?	Yes / No / Unsure
**Q8**	Was the compliance acceptable in all groups?	Yes / No / Unsure
**Q9**	Was the drop-out rate described and acceptable?	Yes / No / Unsure
**Q10**	Was the timing of the outcome assessment in all groups similar?	Yes / No / Unsure
**Q11**	Did the analysis include an intention-to-treat analysis?	Yes / No / Unsure
**Q12**	Are reports of the study free of suggestion of selective outcome reporting?	Yes / No / Unsure

### Quantitative Synthesis

We grouped the results with respect to the interventions used (e.g., acupuncture), the general locus of the pain (e.g., neck or low back), the persistence of the pain (e.g., acute, sub-acute, or chronic), and the cause of pain (e.g., specific or non-specific).

The data abstracted were classified into continuous and dichotomous variables. Generally, fixed-effects models (inverse-variance method) were used in the meta-analysis. However, we also used random-effects models (DerSimonian-Laird method) to pool data if the statistical heterogeneity was high (I^2^ ≥ 50%). The source of heterogeneity was explored by fitting covariables (ie, intervention characteristics, mean age, baseline total symptom scores) one by one in the meta-regression. We analyzed the subgroups according to the source of heterogeneity if possible, and sensitivities if there were unaccountable sources of heterogeneity. We used contour-enhanced funnel plots and Egger test to examine publication bias if the number of pooled trials were near or above 10[16,17]. All analyses were performed in STATA 12.0 (StataCorp LP, College Station, TX).

If the data permitted the assessment and there were statistically significant differences across the pooled data in pain relief or disability improvement, clinical importance was assessed according to Cohen’s 3 levels (Table 2) [18].

**Table 2:** Rating of Clinical Importance.

Rating	Range
**Small**	a weighted mean difference (WMD) less than 10% of the scale (e.g., <10 mm on a 100 mm VAS); a standardized mean difference (SMD) or “d” score <0.5; a relative risk of <1.25 or >0.8 (depending on whether the report referred to the risk of benefit or the risk of harm, respectively)
**Medium**	a WMD from 10–20% of the scale; an SMD or “d” score from 0.5 to 0.8; a relative risk between 1.25 and 2.0 or between 0.5 and 0.8 (depending on the factor described above)
**Large**	a WMD >20% of the scale; an SMD or “d” score ≥ 0.8; a relative risk >2.0 or <0.5 (depending on the factor described above)

### Rating the Strength of Evidence

The overall strength of evidence was evaluated with the aid of the grading system outlined in the Methods Guide prepared by the AHRQ Evidence-based Practice Center (EPC) program [19]. The rating scheme focuses on 4 major domains: risk of bias (high, medium, low), consistency, directness, and precision. Consistency indicated that 75% of the trials showed that effects were in the same direction (positive or negative) or that heterogeneity was low (i.e., I^2^ < 50%). The strength of evidence was classified into one of four levels: high, moderate, low, or insufficient (no evidence) (Table 3, S4 Table) [19]. The level was lowered one stage if any of the aforementioned domains was not met and was directly lowered two stages if the trial had a high risk of bias. Additionally, the level was lowered one stage if the sample size was smaller than 40 patients per group (to enable adequate power) [20].

**Table 3:** Grading of Evidence.

Grade	Domain
**High**	All 4 domains are met (e.g., low risk of bias, precise, direct, consistent)
**Moderate**	1 of the domains is not met (e.g., medium risk of bias, precise, direct, consistent)
**Low**	2–4 of the domains are not met (e.g., high risk of bias, precise, indirect, inconsistent)
Insufficient	No evidence/ absence of evidence

## Results

**Fig 1:**
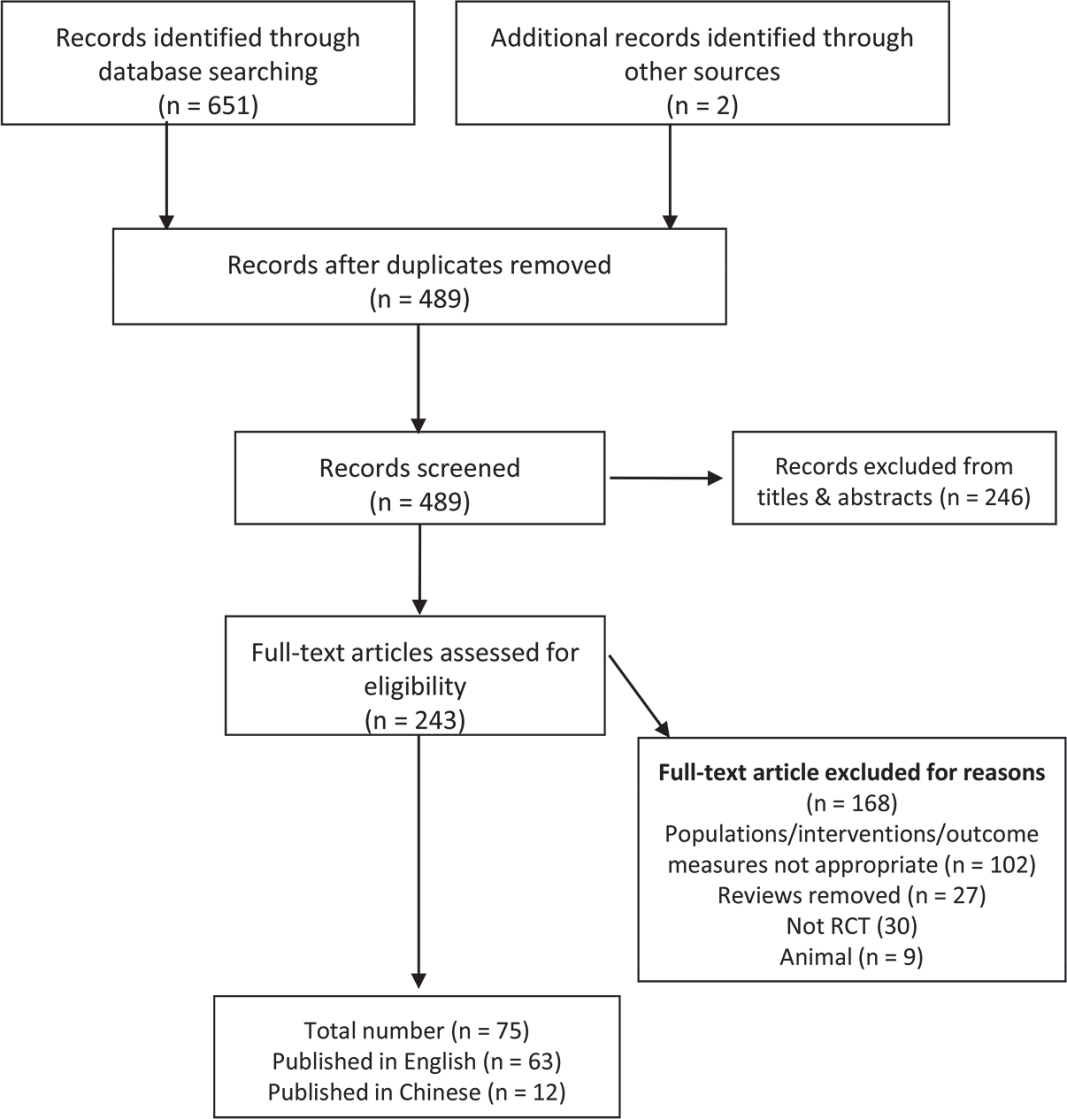
Flow Diagram.

After searching the databases rigorously and systematically, 658 unique records were identified, and the titles and abstracts were screened. The full-text of 243 articles were assessed for eligibility, 75 studies [21–93] were included in the systematic review (Fig. 1). Of these studies, 12 studies [35,36,59,78–81,90–93] were in Chinese (2 unpublished trials about Chinese herbal medicine), others in English. The kappa value for agreement between the reviewers (YQL and LL) was 0.90 which indicated an excellent agreement. We found that most of the included studies were principally about acupuncture, acupressure, and cupping (Table 4). In contrast, the number of studies on the other seven treatments was less than 3 (most = 1) for each treatment. The treatment sessions and treatment durations of interventions also were shown in Table 4. The specific results of our meta-analysis were shown in S5 Table.

**Table 4:** The specific number of studies included and basic characteristics of treatments for each intervention.

Intervention	Condition	Study (N)	Patients (N)	Duration of one treatment (minutes)$	Treatment sessions (times)$	Course of Treatment (weeks)$	Number of acupoints selected$
Acupuncture	NP	17	1434	25 (20, 30)	8.5 (5.8, 10.5)	4 (3, 4.5)	6 (5.8, 10)
	LBP	31	6656	25 (20, 30)	10 (6, 12)	4.5 (3.3, 7)	9.8 (6, 14)
Acupressure	NP	1	32	35–40	8	3	7
	LBP	5	417	15 or 30	8 (6, 9)	4 (3, 4)	7 or 18
Cupping	NP	5	251	10 or 15	5 (4, 6)	2	n.a.
	LBP	6	415	15 or 20	7.5 (3.5, 10)	3 (1.9, 3)	n.a.
Gua sha	NP	2	69	15 or 30	1		n.a.
	LBP	1	19	15	1		n.a.
Qi gong	NP	3	378	60 (45, 90)	18 (12, 18)	12	n.a.
	LBP	0	0				
Tai chi	NP	0	0				
	LBP	1	170	40	18	10	n.a.
Chinese herbal medicine	NP	3	840	n.a.	8 (8, 12)	4	
	LBP	0	0				
Chinese manipulation	NP	3	396	20	8 (8, 10)	4 (4, 5)	n.a.
	LBP	0	0				
Moxibustion	NP	0	0				
	LBP	0	0				
Tuina	NP	0	0				
	LBP	0	0				
Total		75*	11077				

LBP, low back pain; NP, neck pain; N, number; RCT, randomized controlled trial; n.a., not applicable.

*Some of the studies were included into two or more rows.

$The results were shown as median and interquartile range.

### Study characteristics

**Fig 2:**
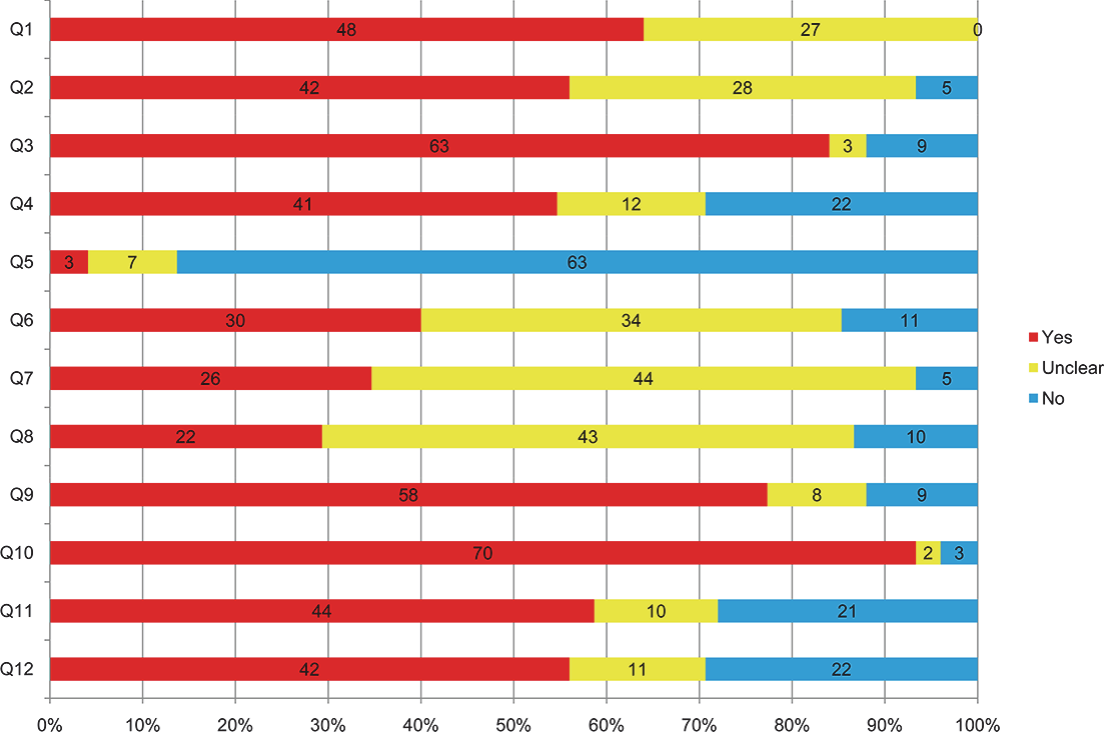
Risk-of-Bias of studies included. Q, question.

Seventy five studies involving 11077 subjects ranging from 17 to 90 years were included. A majority of the participants were females (> 60%) with chronic neck pain (CNP) or chronic low back pain (CLBP). The basic characteristics of the trials were presented in S6 Table. For the findings of our risk-of-bias assessment were shown in Fig. 2 (and S7 Table). The median and interquartile range (IQR) of the quality score of the studies was 6 (4.5 to 8), which meant that the overall quality were of higher-quality. Most of the studies didn’t provide adequate information on outcome assessor blinding, co-intervention and compliance. Given the characteristics of some interventions, the blinding of the care provider was unapplicable. The strength of evidence and its clinical importance was presented in S8 Table.

### Acupuncture in CNP

There were seventeen studies (n = 1434 individuals) identified [21–37]. Two of the studies were published in Chinese[35,36]. The IQR of the quality score of the studies was 5 (4 to 7).

Acupuncture versus sham-acupuncture

**Fig 3:**
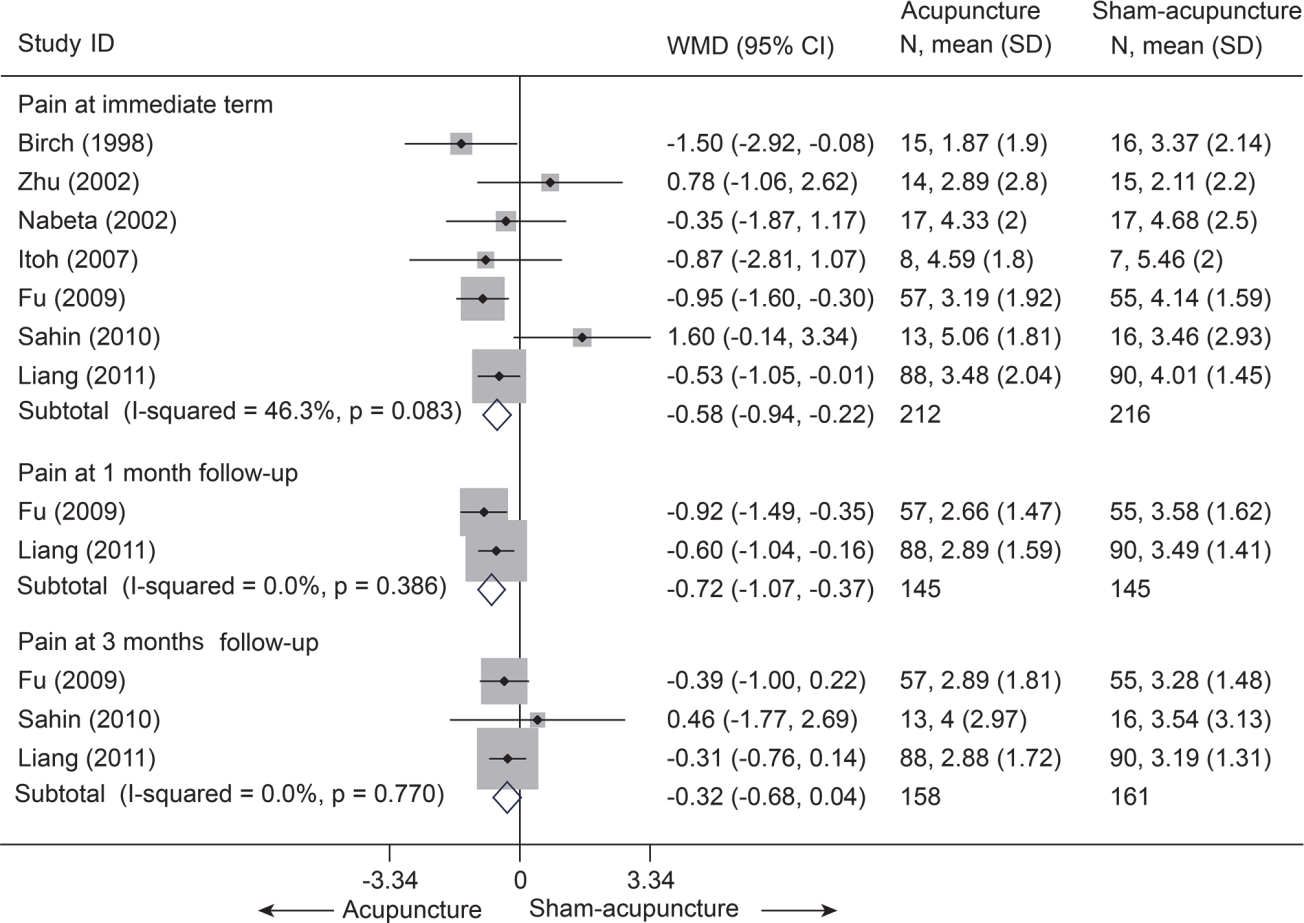
Meta-Analysis of Acupuncture versus Sham-Acupuncture for CNP in Pain Intensity on the VAS (0–10 mm). Fixed-effects model was used; CI, confidence interval; CNP, chronic neck pain; SD, standard deviation; VAS, visual analogue scale; WMD, weighted mean difference.

**Fig 4:**
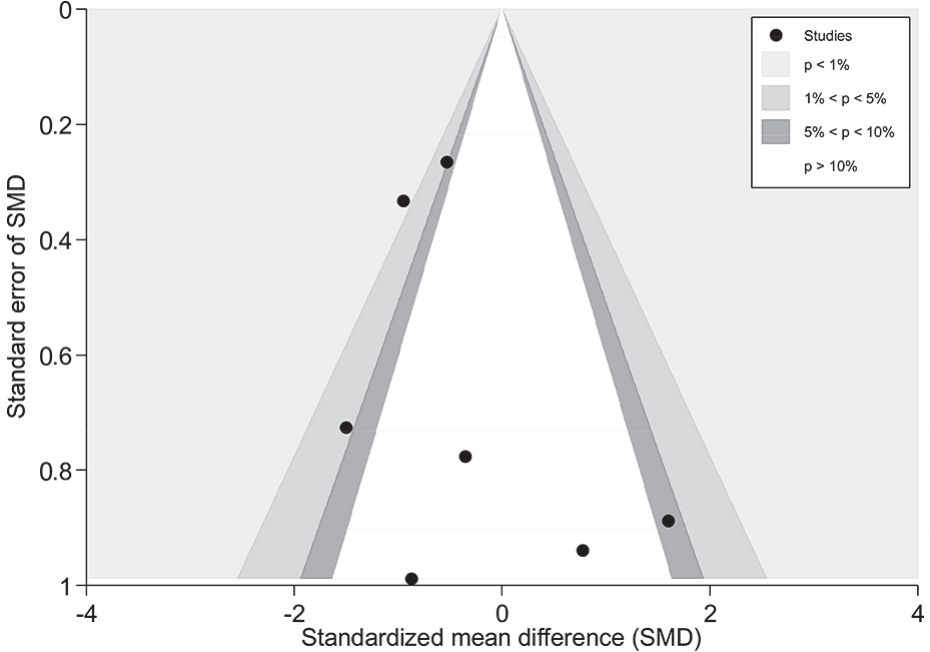
Contour-enhanced funnel plot of Acupuncture versus Sham-Acupuncture for CNP in Pain. Visual inspection of the funnel plot suggested symmetry. Specifically, there were most of trials with negative results (i.e., more trials in areas of statistical nonsignificance), indicating no evidence of publication bias.

**Fig 5:**
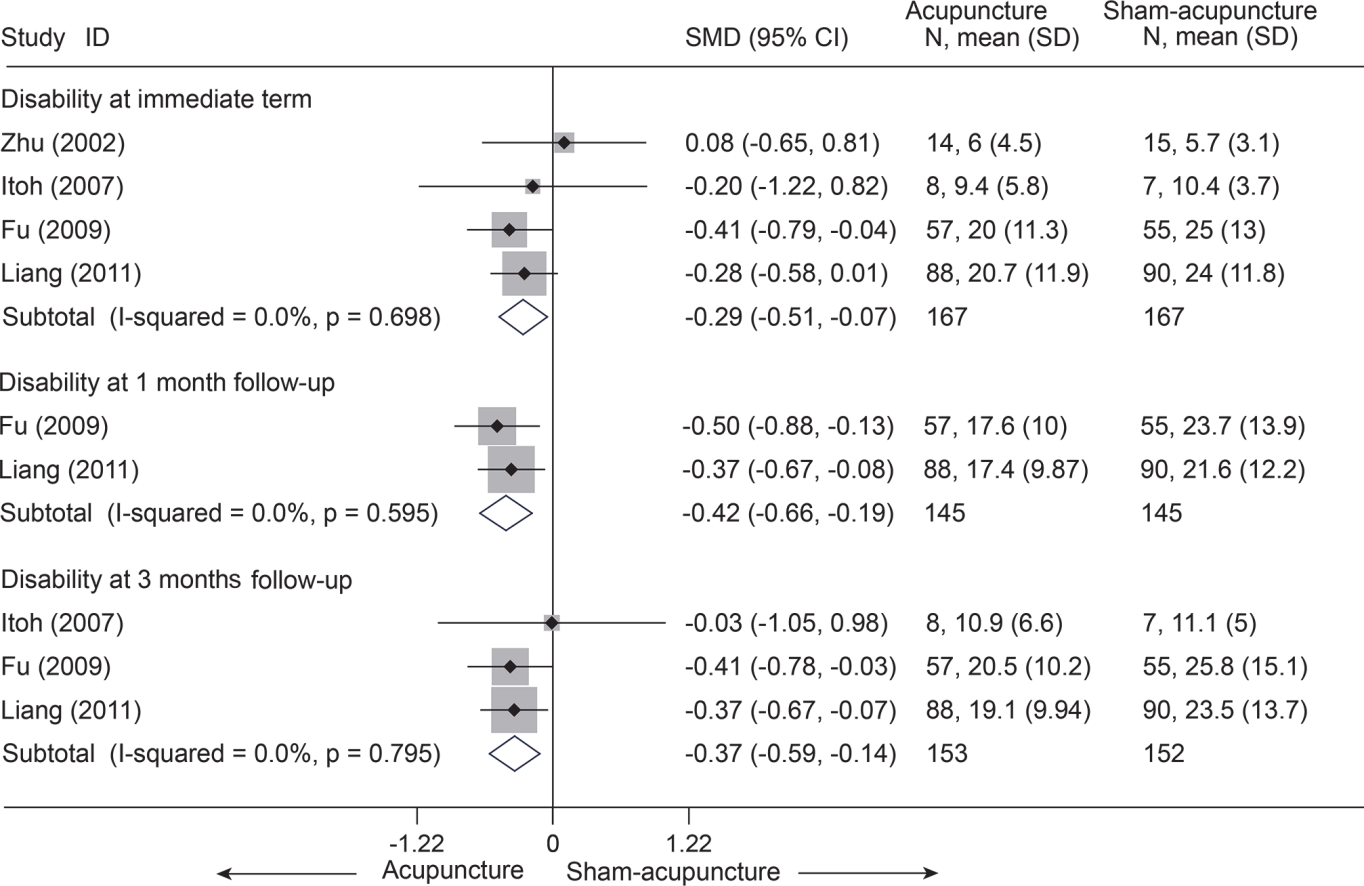
Meta-Analysis of Acupuncture versus Sham-Acupuncture for CNP in Disability. Fixed-effects model was used; CI, confidence interval; CNP, chronic neck pain; SD, standard deviation; SMD, standardized mean difference.

There were seven trials (428 subjects) identified [21–24,27,28,30].We observed significant differences in pain relief in favor of acupuncture compared with the sham group (VAS 10 cm, MD, -0.58 [-0.94, -0.22], I^2^ = 46.3%) (Fig. 3 and S5 Table)[21–24,27,28,30],. The contour-enhanced funnel plot indicated symmetry (Fig. 4) and the Egger test suggested there was no evidence of publication bias (coefficient = 1.00; SE = 1.05; P = 0.39). This superiority persisted until 1 month post-intervention (MD, -0.72 [-1.07, -0.37])[21,23], whereas after 3 months of follow-up, this effect gradually diminished until there were no differences between the groups (MD, -0.32 [-0.68, 0.04])[21–23]. However, with respect to disability (Fig. 5)[21,23,24,27], this tendency in favor of acupuncture was also displayed in these terms.

Acupuncture versus sham treatments (inactive treatments)

**Fig 6:**
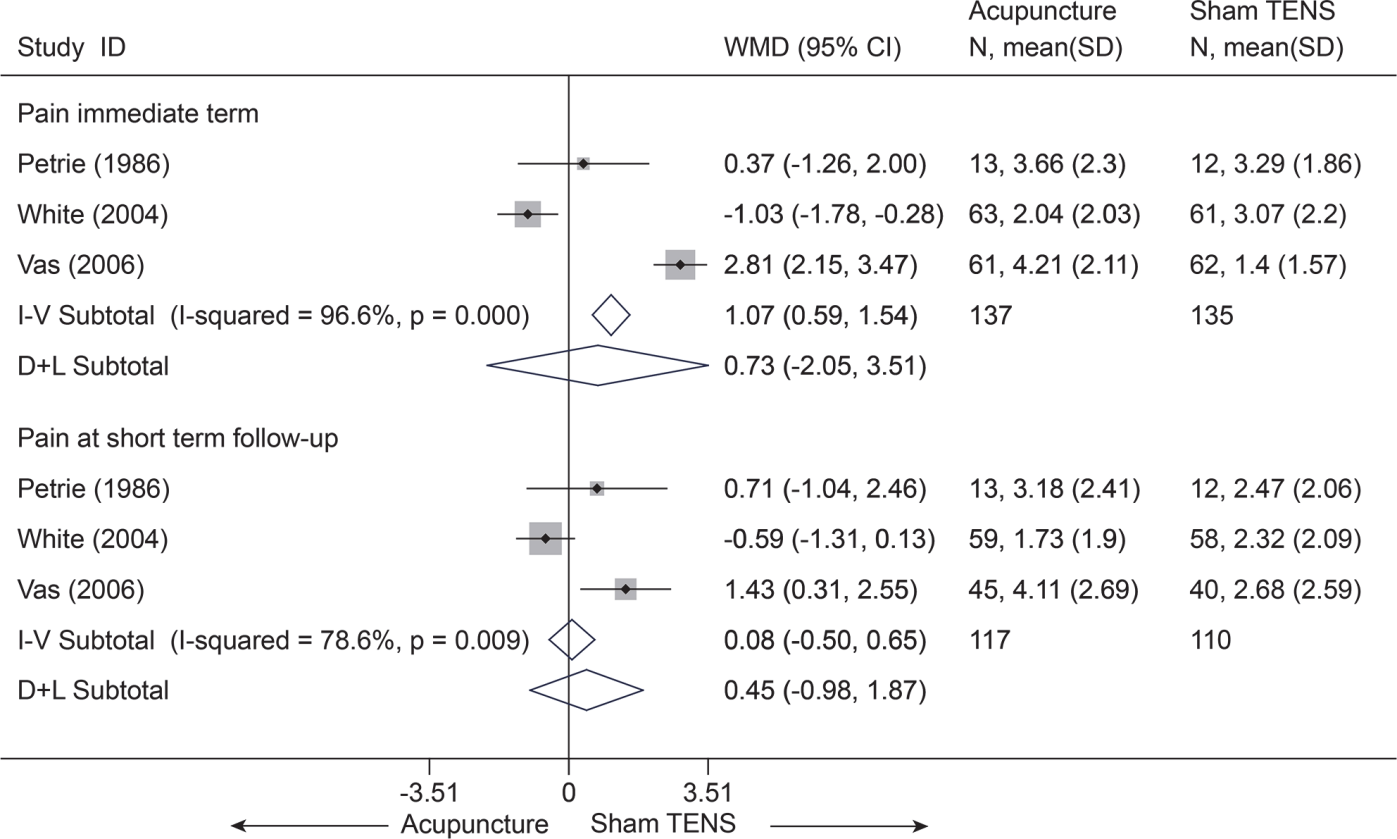
Meta-Analysis of Acupuncture versus Sham-TENS for CNP in pain on VAS 10 cm. I-V, inverse-variance method (fixed-effects model); D+L, DerSimonian-Laird method (random-effects model); CI, confidence interval; CNP, chronic neck pain; SD, standard deviation; WMD, weighted mean difference.

**Fig 7:**
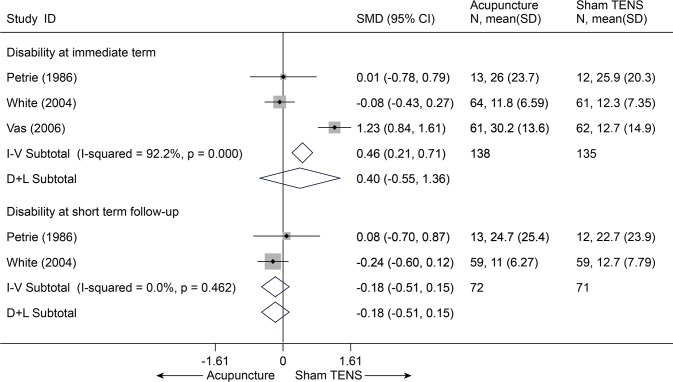
Meta-Analysis of Acupuncture versus Sham-TENS for CNP in Disability. I-V, inverse-variance method (fixed-effects model); D+L, DerSimonian-Laird method (random-effects model); CI, confidence interval; CNP, chronic neck pain; SD, standard deviation; SMD, standardized mean difference.

There were three trials (272 subjects) identified that hold comparisons between acupuncture and sham transcutaneous electrical nerve stimulation (TENS) [25,26,31]. Compared with sham TENS for pain, acupuncture didn’t display any differences in pain relief (Fig. 6) and disability improvement (Fig. 7) for CNP at immediate term and even at short term (p > 0.10), and these results were still robust in sensitivity analysis[25,26,31]. One trial (108 subjects) compared acupuncture and sham laser [29]. Similarly, no difference was found between acupuncture and sham laser in pain relief immediately post-treatment (p = 0.202) (S5 Table) [29].

Acupuncture versus waitlist (no treatment)

Only one trial (30 subjects) showed a significant difference in pain for CNP immediately post-treatment on VAS 10 cm, with an odds ratio of 26.00 (3.69 to 183.42, p = 0.001)[37].

Acupuncture versus active treatments

**Fig 8:**
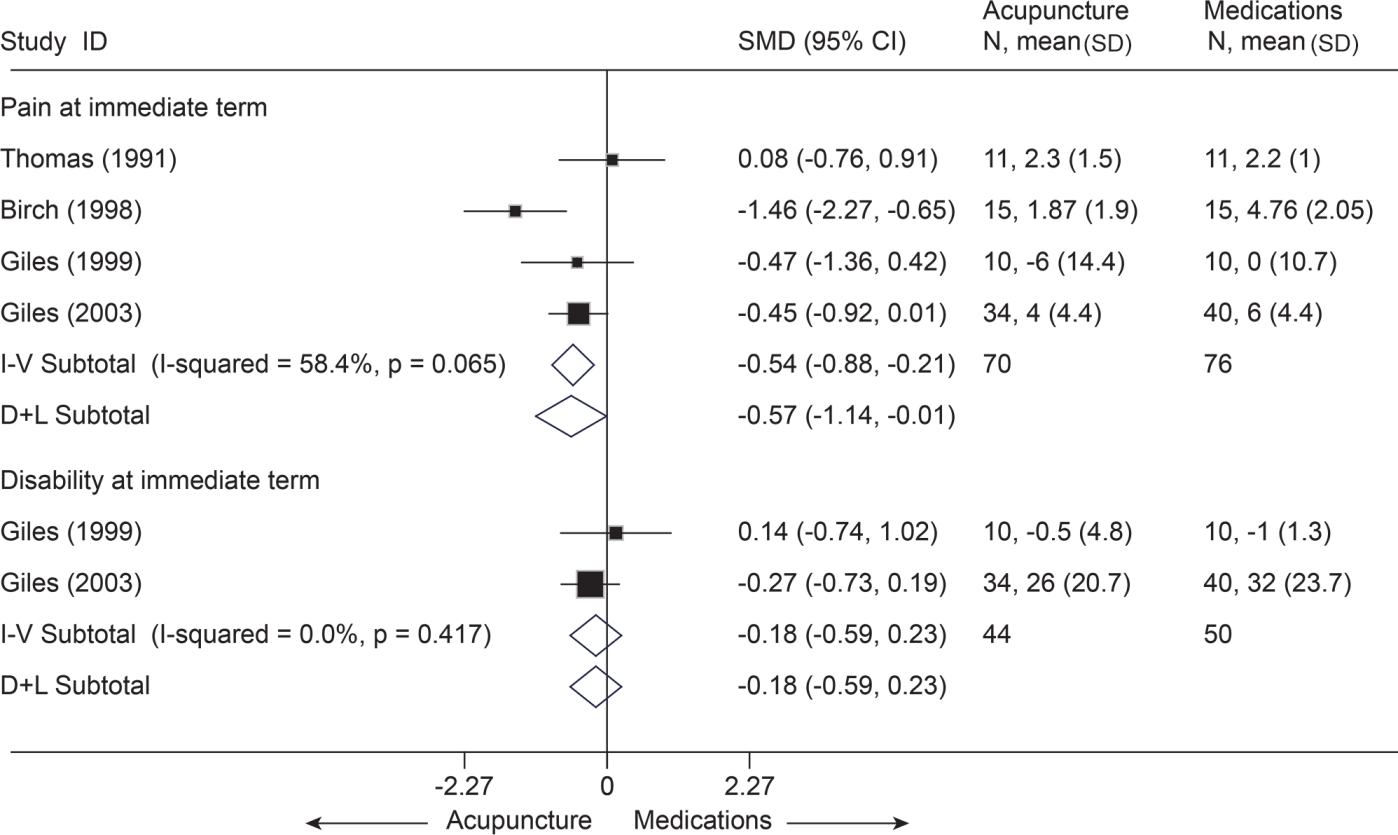
Meta-Analysis of Acupuncture versus Medications for CNP in Pain and Disability. I-V, inverse-variance method (fixed-effects model); D+L, DerSimonian-Laird method (random-effects model); CI, confidence interval; CNP, chronic neck pain; SD, standard deviation; SMD, standardized mean difference.

**Fig 9:**
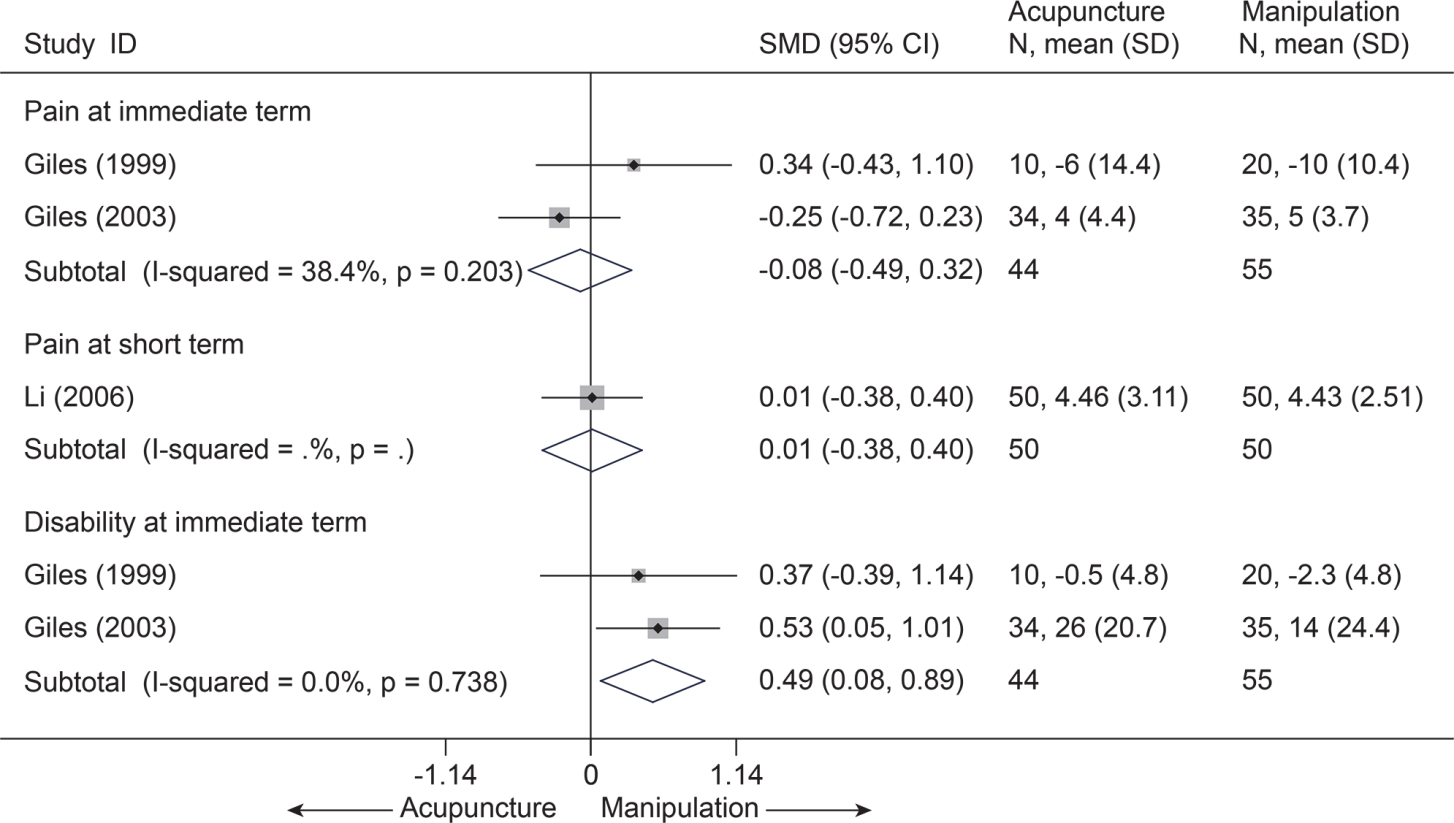
Meta-Analysis of Acupuncture versus Manipulation for CNP in Pain and Disability. Fixed-effects model was used; CI, confidence interval; CNP, chronic neck pain; SD, standard deviation; SMD, standardized mean difference.

With respect to findings comparing acupuncture with other active treatments, such as medications with a SMD of-0.57 [-1.14, -0.01][30,32–34], massage with a MD of-1.63 [-2.68, -0.58] on VAS 10 cm [29], significant superiority favoring acupuncture was found about pain relief at immediate term (p<0.05) (Fig. 8 and S5 Table), but the result was not robust after sensitivity analysis was performed (p = 0.06). Whereas, acupuncture was even inferior to manipulation (SMD, -0.08 [-0.49, 0.32], I^2^ = 38.4%) (Fig. 9)[32,33,35] and cervical traction (VAS 10 cm, MD, 1.31 [0.78, 1.84]) (S5 Table)[36].

Side effects

There were some complaints reported in the trials, such as local bleeding, numbness, pain, and fainting when needles were inserted in some subjects. All these side effects were transient and mild. No life-threatening side effects were reported [21,25,26,29].

Summary

Several studies showed that there was moderate evidence with small clinical importance that acupuncture was more effective than sham-acupuncture in reducing pain and disability associated with CNP in the immediate term and at the one-month follow-up.

### Acupuncture in LBP

Thirty one studies with a total of 6656 patients compared acupuncture with other treatments in low back pain about pain or disability[32,33,38–48,50–65,94]. The IQR of the quality score of the studies was 7 (5 to 9).

Acupuncture versus sham-acupuncture

**Fig 10:**
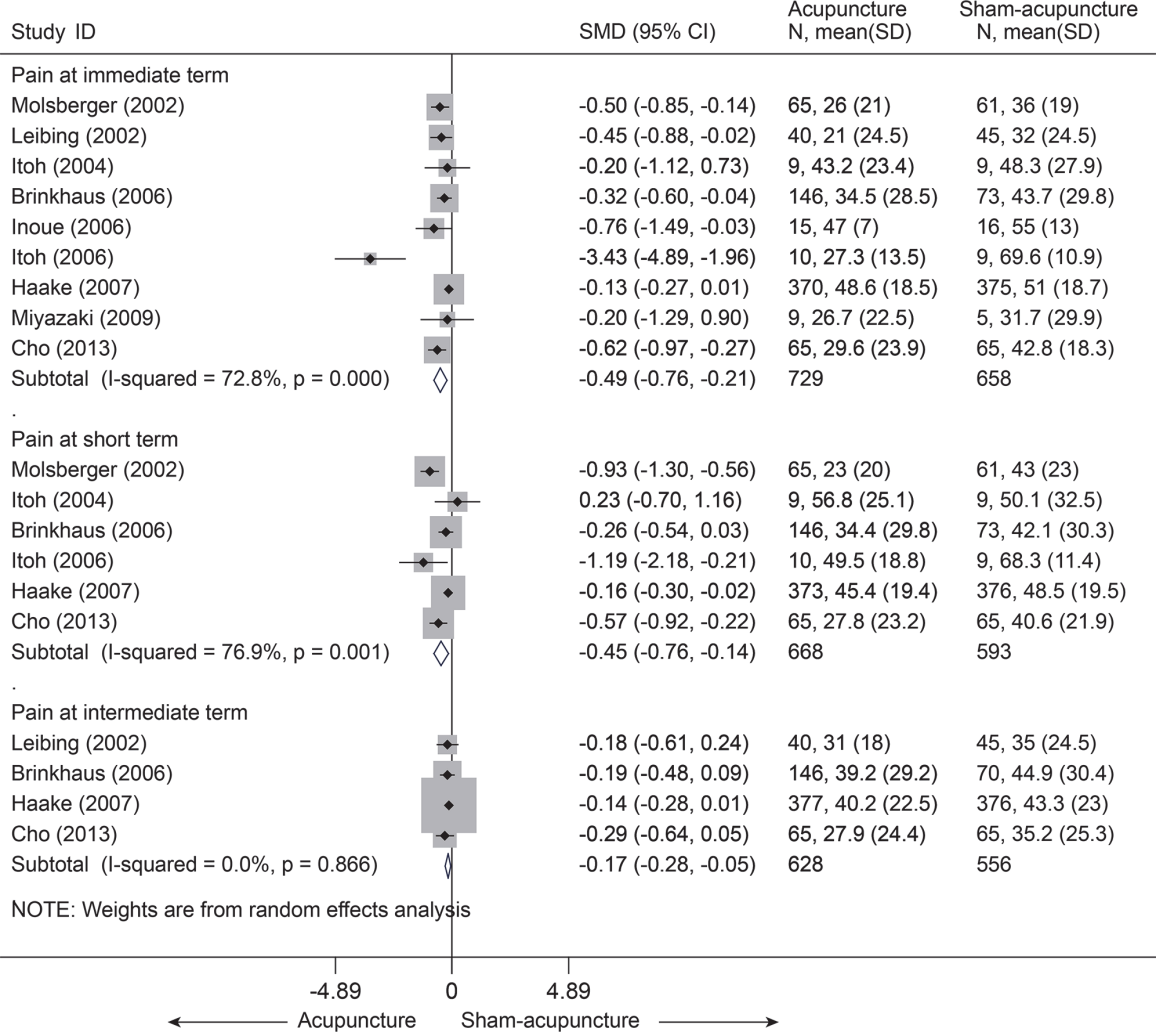
Meta-Analysis of Acupuncture versus Sham-acupuncture for CLBP in Pain. CI, confidence interval; CLBP, chronic low back pain; SD, standard deviation; SMD, standardized mean difference.

**Fig 11:**
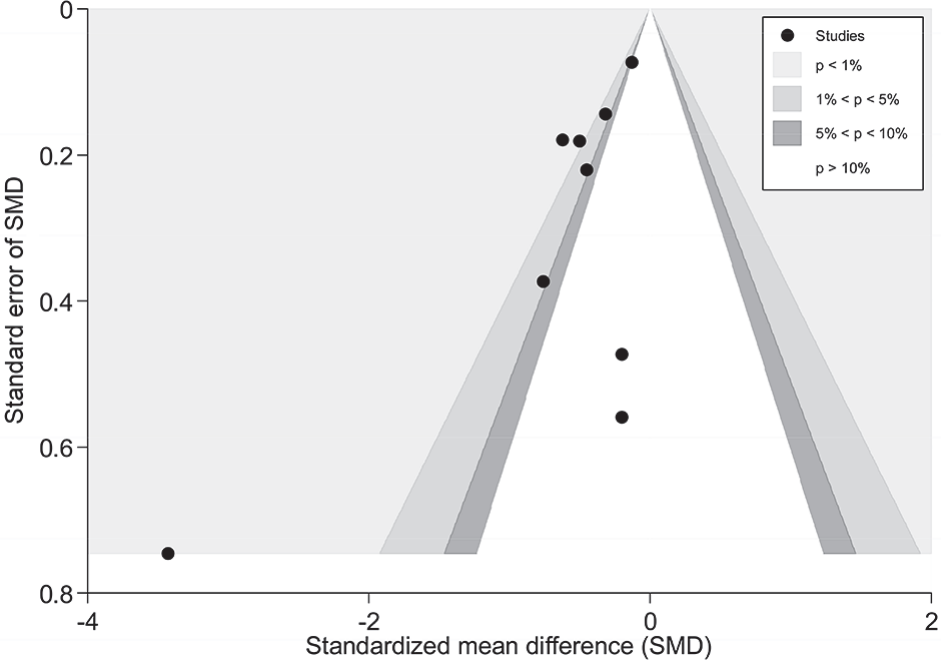
Contour-Enhanced Funnel Plot of Acupuncture versus Sham-acupuncture for CLBP in Pain at immediate term. Visual inspection of the funnel plot suggested some degree of asymmetry. Specifically, there was a relative lack of trials with negative results (i.e., fewer trials in areas of statistical nonsignificance), indicating a potential for publication bias; meanwhile, the dot on the lower left part of the Figure suggested an evidence of small-study effect.

**Fig 12:**
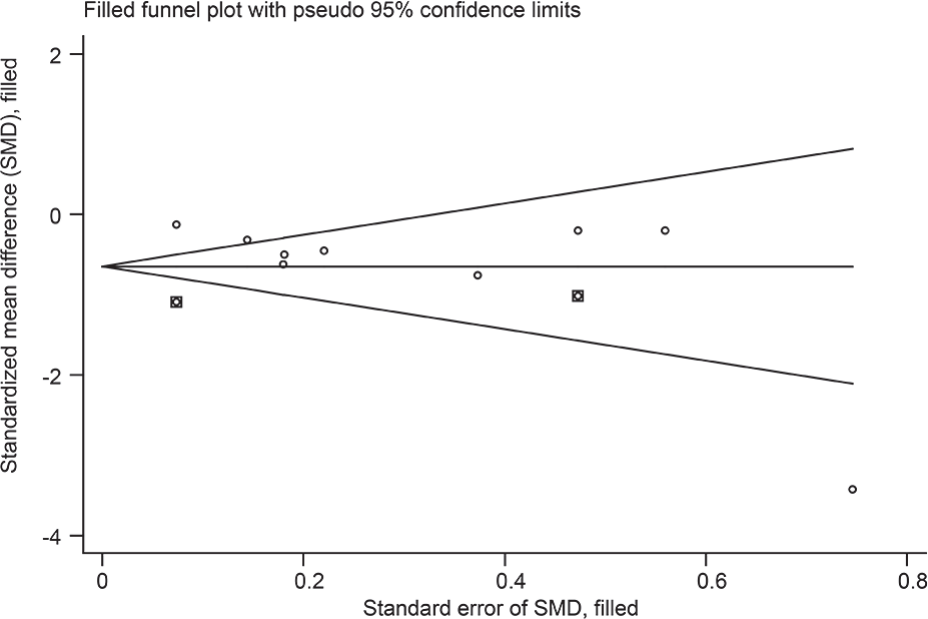
Metatrim Funnel Plot of Acupuncture versus Sham-acupuncture for CLBP in Pain at immediate term. The dots in the squares were the studies filled. There were two trials with positive effects filled.

**Fig 13:**
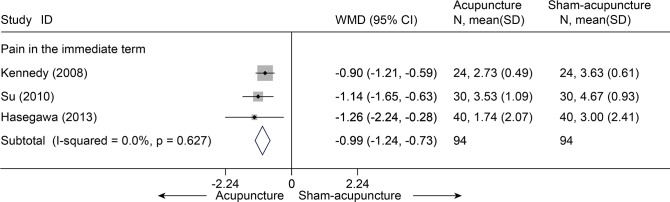
Meta-Analysis of Acupuncture versus Sham-acupuncture for acute LBP in pain on VAS 10 cm. Fixed-effects model was used; CI, confidence interval; LBP, low back pain; SD, standard deviation; WMD, weighted mean difference.

**Fig 14:**
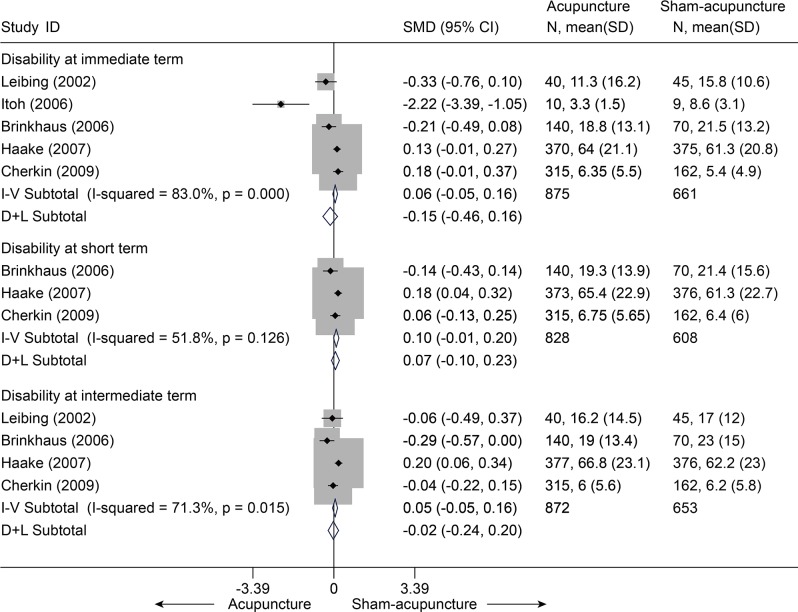
Meta-Analysis of Acupuncture versus Sham-acupuncture for CLBP in Disability. CI, confidence interval; CLBP, chronic low back pain; SD, standard deviation; SMD, standardized mean difference.

Thirteen studies[38–48,50,51] compared acupuncture and sham-acupuncture, of which ten studies[38–47] were about CLBP (n = 1864) and the remaining three[48,50,51] about acute LBP (n = 188). With respect to pain reduction, nine studies [38,39,41–47] (n = 1387) showed that acupuncture was clinically superior to sham acupuncture for CLBP immediately post-treatment (SMD = -0.49, 95% CI-0.76 to-0.21) and up to 3 months post-treatment (SMD = -0.45, 95% CI-0.76 to-0.14), but these were highly heterogeneous across studies (I^2^ = 72.8% and 76.9%, respectively) (Fig. 10). The source of these heterogeneities was not apparent. A sensitivity analysis still yielded robust results and decreased the heterogeneities (I^2^ = 39.7% and 56.2%, respectively) after small studies with favorable treatment effect were removed (S5 Table). The Egger test indicated publication bias due to small-study effects (coefficient = -2.18; P = 0.031) about pain at immediate term. The contour-enhanced funnel plot showed an asymmetry (Fig. 11), and adjusting for this bias removed a small study [42] with favorable treatment effect (coefficient = -1.37; 95% CI, -3.05 to 0.30; P = 0.092). We performed metatrim, and two assumed studies with favorable effect were added and the pooled result was still robust and in favor of acupuncture (SMD = -0.65, 95% CI-1.00 to-0.30, random) (Fig. 12). This positive effectiveness also persisted in individuals with acute LBP in the immediate term with a MD of-0.99 (VAS10 cm, 95% CI-1.24 to-0.73) (Fig. 13) [48,50,51]. However, regarding disability improvement, no significant differences were observed between groups for CLBP (Fig. 14 and S5 Table).

Acupuncture versus waitlist (no treatment)

**Fig 15:**
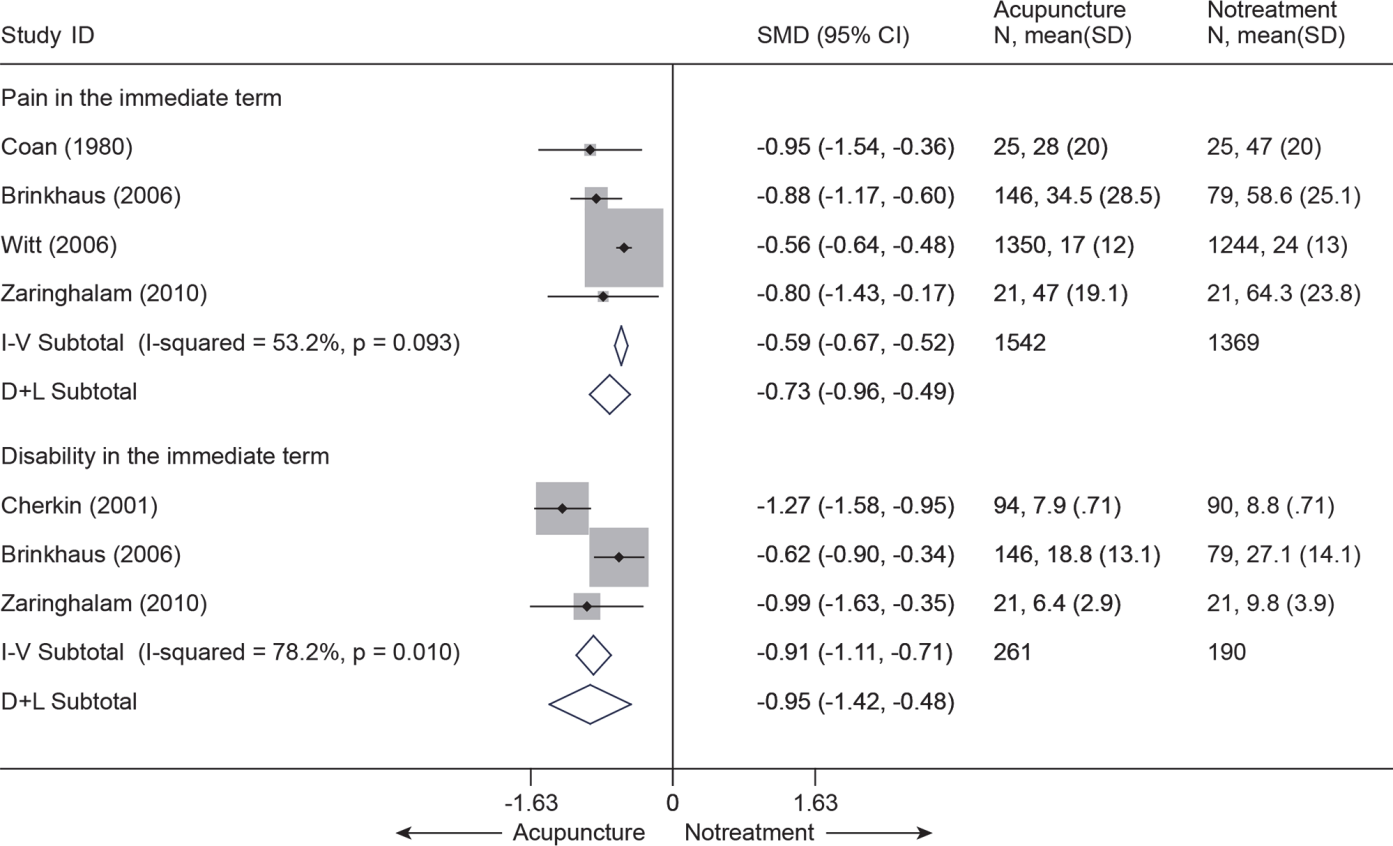
Meta-Analysis of Acupuncture versus Notreatment for CLBP in Pain and Disability. I-V, inverse-variance method (fixed-effects model); D+L, DerSimonian-Laird method (random-effects model); CI, confidence interval; CLBP, chronic low back pain; SD, standard deviation; SMD, standardized mean difference.

Four trials [44,52,53,55] (n = 2911) compared acupuncture with no treatment with respect to pain relief and disability improvement for CLBP. All four studies that evaluated the immediate relief of pain showed superiority in favor of acupuncture (SMD = -0.73, 95% CI-0.96 to-0.49) (Fig. 15). Meanwhile, three studies [44,52,55] were pooled according to the levels of function, a significant advantage emerged favoring acupuncture immediately post-treatment (SMD = -0.95 (-1.42, - 0.48)). The results above were still robust in sensitivity analyses (S5 Table).

Acupuncture versus TENS

**Fig 16:**
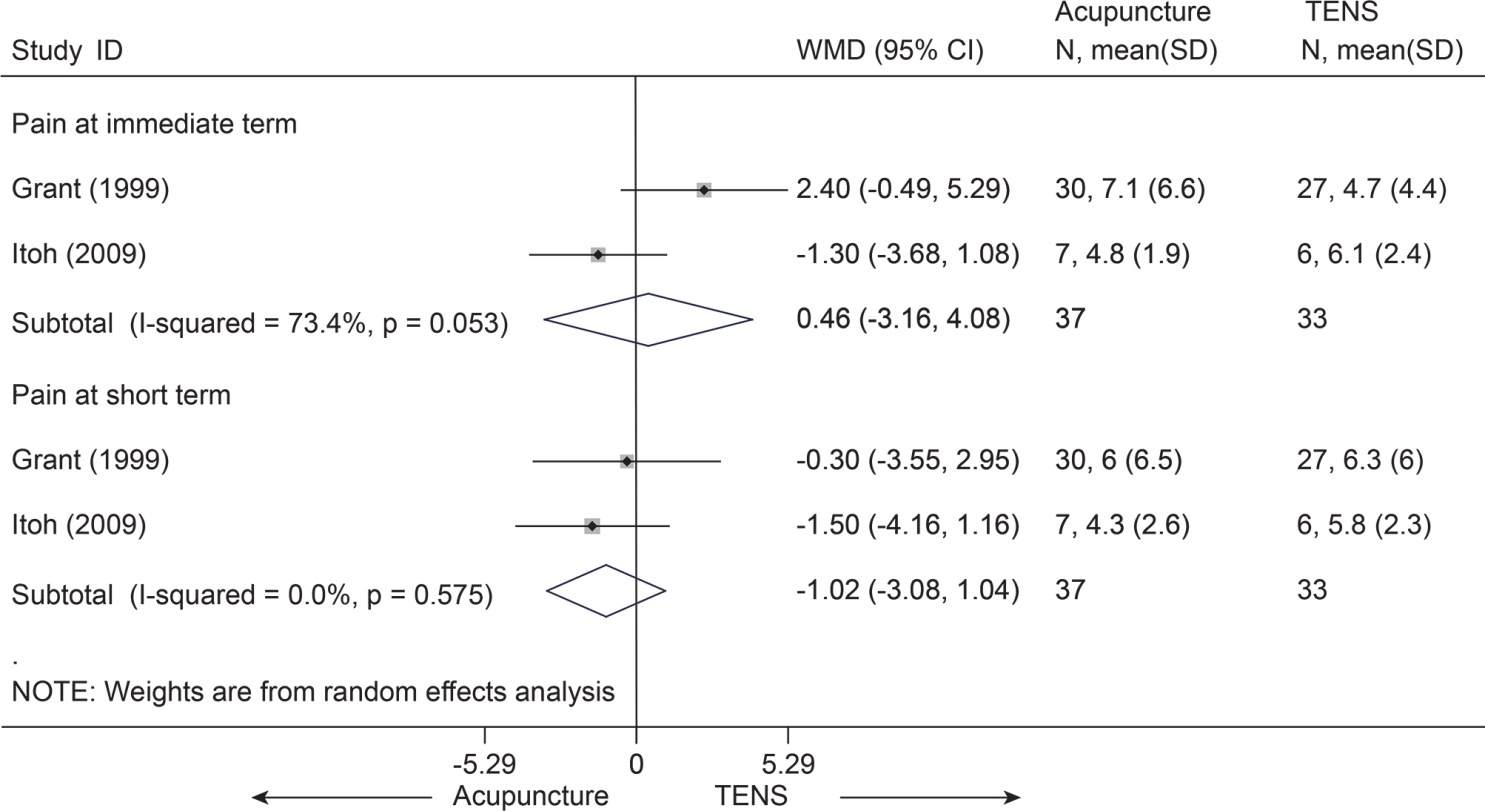
Meta-Analysis of Acupuncture versus TENS for CLBP in pain on VAS 10 cm. TENS, transcutaneous electrical nerve stimulation; CI, confidence interval; CLBP, chronic low back pain; SD, standard deviation; WMD, weighted mean difference.

Two studies [56,57] (n = 70) compared acupuncture with TENS and showed no significant differences between groups with respect to pain (Fig. 16), not only in the immediate term (p = 0.81) but also at short term follow-up (p = 0.33). However, functional status was not assessed.

Acupuncture versus medications

**Fig 17:**
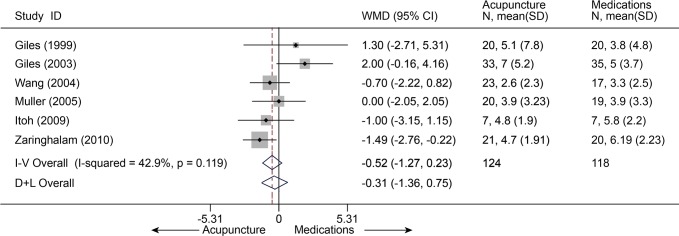
Meta-Analysis of Acupuncture versus Medications for CLBP in pain on VAS 10 cm. Fixed-effects model was used; CI, confidence interval; CLBP, chronic low back pain; SD, standard deviation; WMD, weighted mean difference.

**Fig 18:**
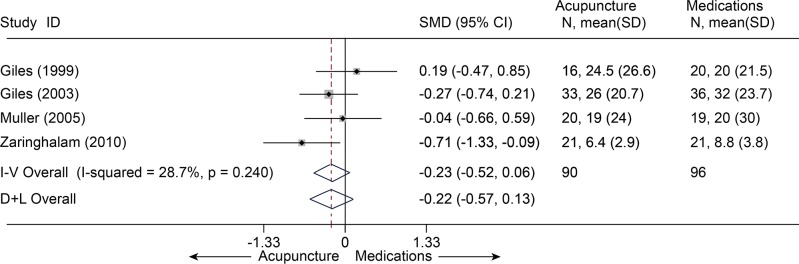
Meta-Analysis of Acupuncture versus Medications for CLBP in disability. I-V, inverse-variance method (fixed-effects model); D+L, DerSimonian-Laird method (random-effects model); CI, confidence interval; CLBP, chronic low back pain; SD, standard deviation; SMD, standardized mean difference.

Six studies [32,33,52,56,58,59] (n = 242) comparing acupuncture with medications about pain at immediate term had a pooled MD of-0.52 (95% CI, -1.27 to 0.23, VAS 10 cm) (Fig. 17). Four studies [32,33,52,58] (n = 186) compared disability at immediate term between acupuncture and medications (Fig. 18). The estimated SMD was-0.23 (95% CI, -0.52 to 0.06). However, these differences not statistically significant. The heterogeneities were small about pain and disability.

Acupuncture plus usual care versus usual care

**Fig 19:**
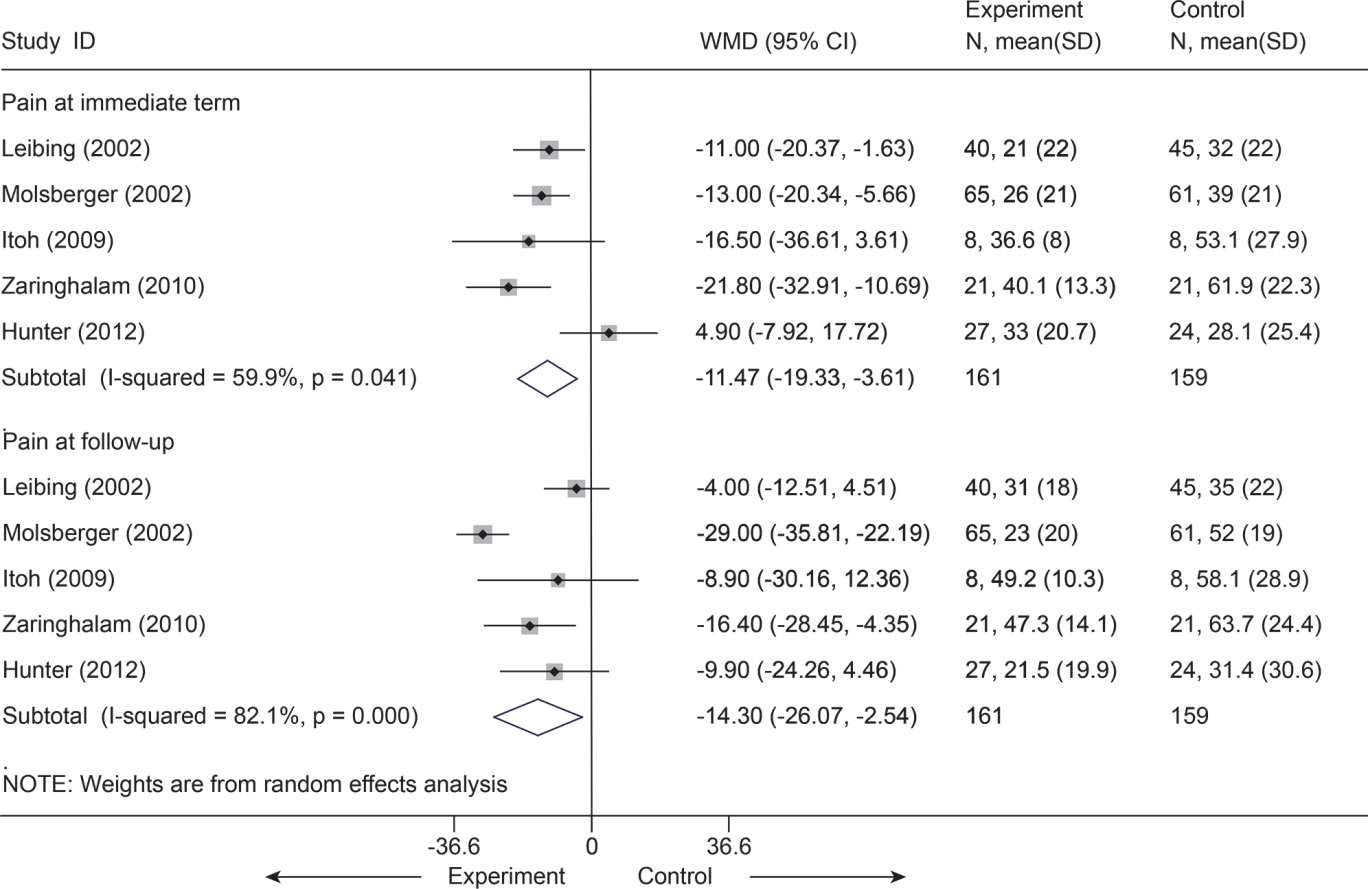
Meta-Analysis of Acupuncture plus UC versus UC for CLBP in pain on VAS 10 cm. UC, usual care; CI, confidence interval; CLBP, chronic low back pain; SD, standard deviation; WMD, weighted mean difference.

**Fig 20:**
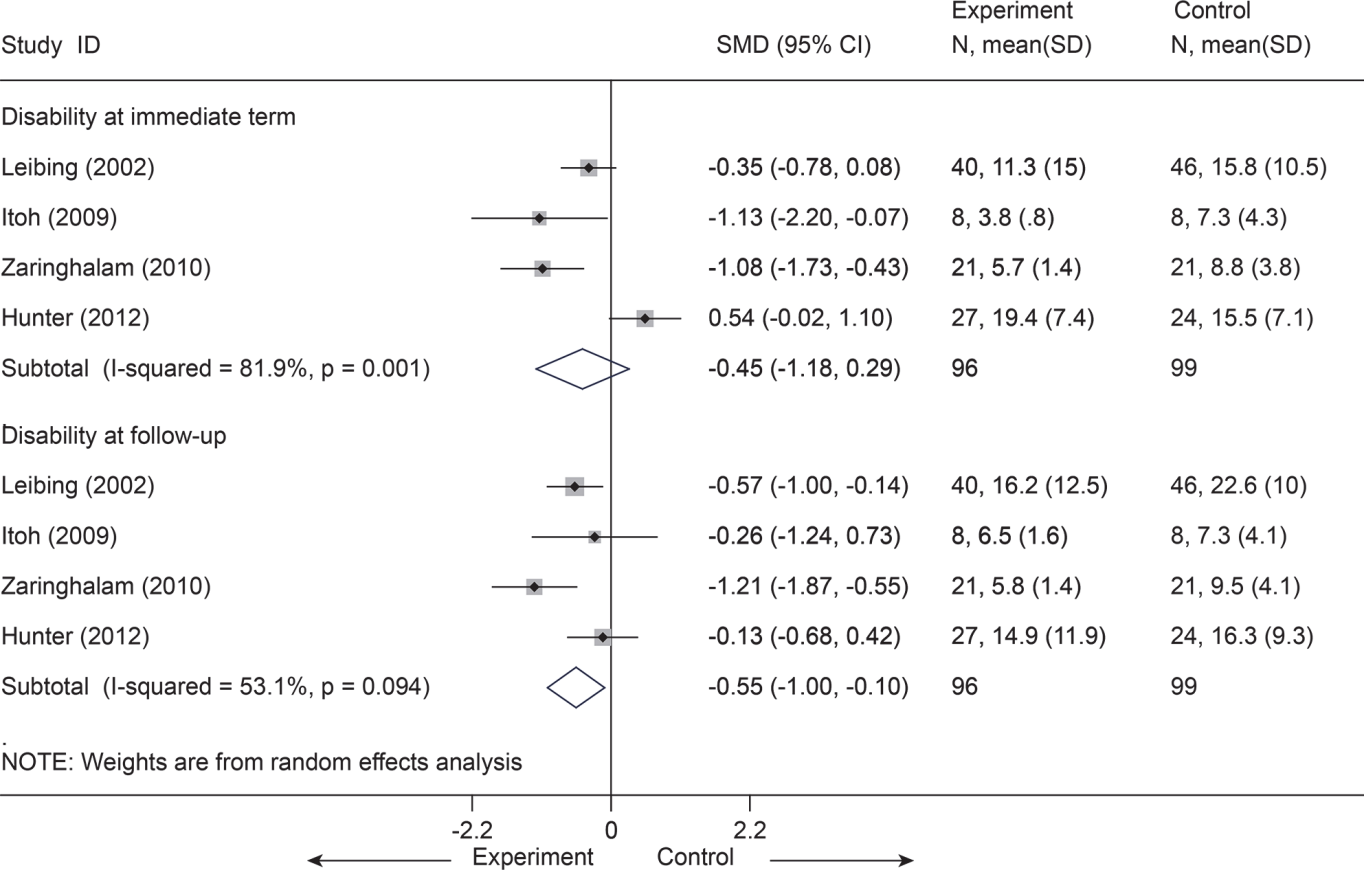
Meta-Analysis of Acupuncture plus UC versus UC for CLBP in Disability. UC, usual care; CI, confidence interval; CLBP, chronic low back pain; SD, standard deviation; standardized mean difference.

Usual care indicated that participants received no study-related care—just the care, if any, that they and their physicians chose: mostly massage and physical therapy visits and continued use of medications (mostly nonsteroidal anti-inflammatory drugs). Five studies [46,47,52,56,66] (n = 320) were identified and categorized for this comparison. After data were pooled for pain reported according to the VAS (100 mm), a significant difference was observed in favor of acupuncture administered concomitantly over usual care for pain associated with CLBP immediately post-intervention (MD = -11.47, 95% CI-19.33 to-3.61, I^2^ = 59.9%) (Fig. 19). In sensitivity analyses limited the 4 studies [46,47,52,56] with adequate randomization, the MD was-14.41 (95% CI, -19.38 to-9.45, I^2^ = 0) on VAS 100 mm Homogeneous effectiveness was reported at follow-up as well with a MD of-14.30 (95% CI, -26.07 to-2.54, I^2^ = 85.6%) on VAS 100 mm[46,47,52,56,66]. The result was still robust after one study [46] with effectiveness was dropout (VAS 100 mm, MD = -0.85, 95% CI-14.50 to-2.50, I^2^ = 0) (S5 Table). Meanwhile, with respect to functional status, after 4 studies [47,52,56,66] (n = 195) were pooled (Fig. 20), results in favor of the intervention group were also found at follow-up (SMD = -0.55, 95% CI-1.00 to-0.10). However, the function at immediate term showed no difference between groups (P = 0.231).

Acupuncture versus usual care

**Fig 21:**
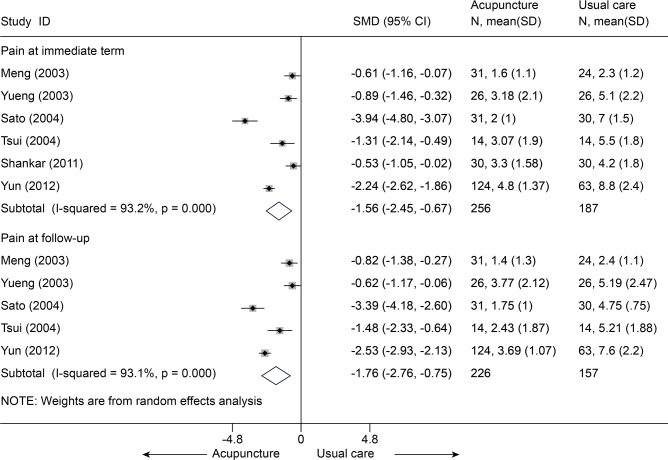
Meta-Analysis of Acupuncture versus Usual Care for CLBP in pain. CI, confidence interval; CLBP, chronic low back pain; SD, standard deviation; standardized mean difference.

Six studies [60–65] (n = 443) compared mean pain score between acupuncture and usual care (S6 Table). All these six studies reported the pain at immediate term (Fig. 21), the SMD in the random-effects model was-1.56 (95% CI, -2.45 to-0.67), which was in favor of acupuncture, but this was highly heterogeneous across studies (I^2^ = 93.2%). The source of this heterogeneity was not apparent. A sensitivity analysis limited to the 4 studies [61,62,64,65] with adequate sequence generation yielded an SMD of-0.75 (95% CI, -1.04 to-0.46, I^2^ = 0). Five of these studies [60,62–65] (n = 383) reported the pain at follow-up term, the SMD was-1.76 (95% CI, -2.76 to-0.75, I^2^ = 93.1%), The SMD of a sensitivity analysis was-0.86 (95% CI, -1.21 to-0.50, I^2^ = 29.7%) [62,64,65].

Side effects

There were a total of 10 subjects who reported side effects in the subsequent trial (130 individuals), namely temporary worsening of LBP (4); pain (2); bruising (1) at the site of insertion; shoulder pain (2); and pain, numbness, or other side effects in the leg (including the knee) (1) [38]. Another subsequent study reported pain (14%), redness (2%), and minor bleeding (1%) at the acupuncture site [66].

Summary

Several studies showed that there was low evidence that acupuncture was more effective than sham-acupuncture, waitlist care, or usual care in reducing pain and disability for CLBP immediately post-treatment. Moreover, three small studies showed that acupuncture may be more effective than sham-acupuncture in reducing pain in the immediate term for acute or sub-acute LBP (moderate evidence).

### Acupressure in NP

We found one study (32 individuals), which was of fair quality but had a small sample size [70].

Acupressure (+ acupoint stimulation + CT) versus conventional therapy (CT) (sub-acute LBP)

Compared with the control group, reduction in the pain intensity in the intervention group was statistically significant at the 1-month follow-up (approximately a 23% reduction in the VAS, p = 0.02, effect size = 0.43) [70].

Side effects

None reported.

Summary

The sample size was so small that we were unable to draw any definite conclusions.

Acupressure in CLBP

We found five RCTs (n = 407) [67–69,71,72]. Almost all of the studies were fair in quality and were conducted in CLBP patients.

Acupressure versus physical therapy

**Fig 22:**
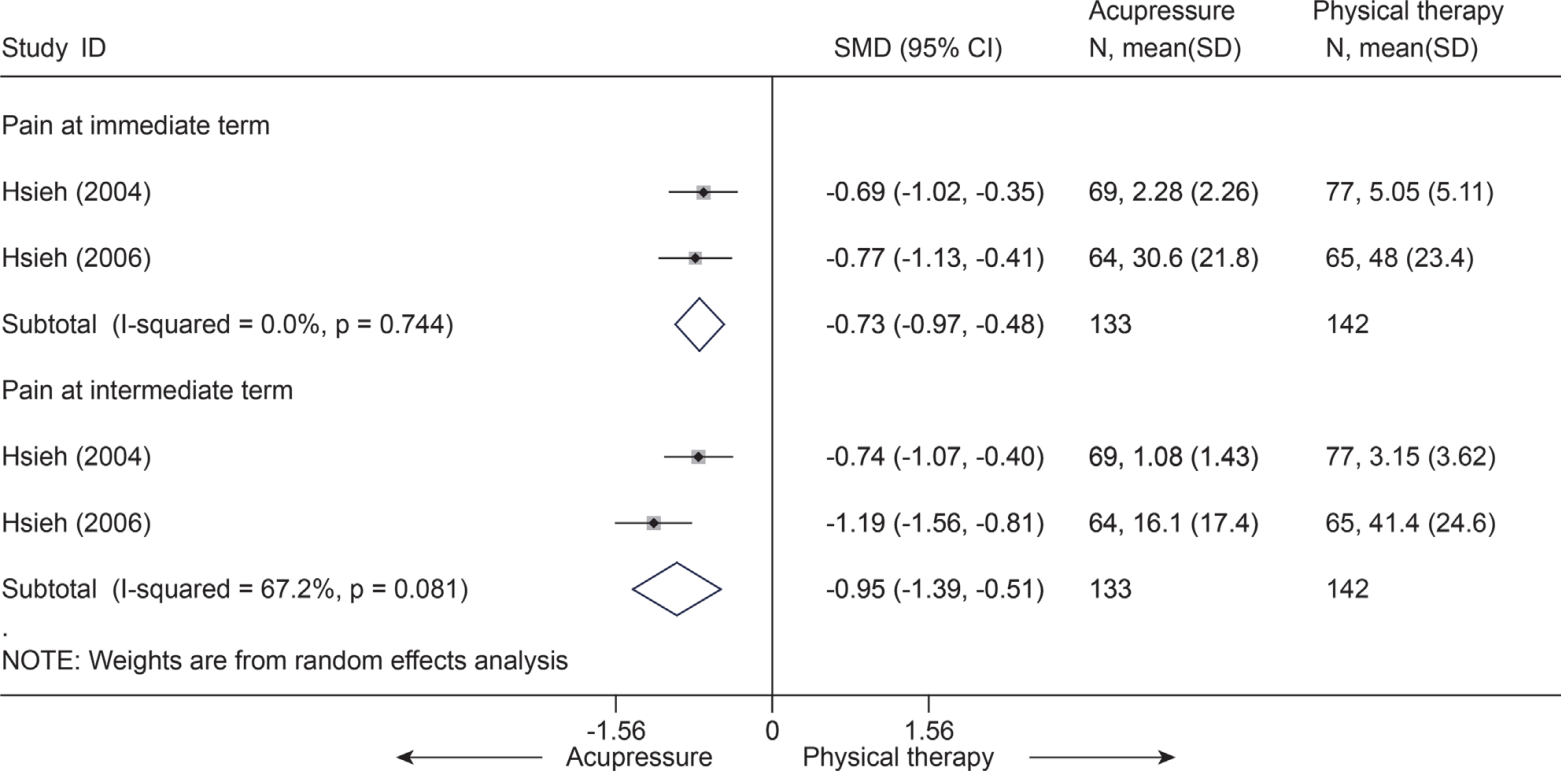
Meta-Analysis of Acupressure versus Physical Therapy for CLBP in Pain. CI, confidence interval; CLBP, chronic low back pain; SD, standard deviation; standardized mean difference.

Two studies [67,71] (n = 275) compared mean pain score between acupressure and physical therapy. The SMDs in pain were-0.73 (95% CI, -0.97 to-0.48) at immediate term and-0.95 (95% CI, -1.39 to-0.51) at intermediate term (Fig. 22). One of the studies [71] reported the disability in RMQ (0–24) scale with MDs of-3.8 (95% CI, -5.7 to-1.9) at immediate term and-4.5 (95% CI, -6.1 to-2.9) at intermediate term.

Acupressure versus sham acupressure

**Fig 23:**
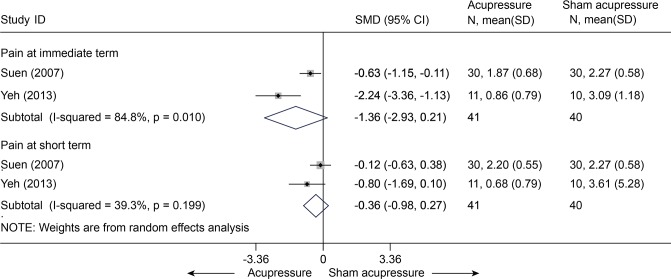
Meta-Analysis of Acupressure versus Sham-Acupressure for CLBP in Pain. CI, confidence interval; CLBP, chronic low back pain; SD, standard deviation; standardized mean difference.

Two studies [68,69] comparing acupressure and sham acupressure in pain were pooled (n = 81). The SMDs were-1.36 (95% CI, -2.93 to 0.21) at immediate term and-0.36 (95% CI, -0.98 to 0.27) at short term (Fig. 23). It indicated that there was no significant difference in pain between these two groups. One of these two studies [68] (n = 21) evaluated the disability score in RMQ between groups with MDs of-5.33 (95% CI, -9.81 to-0.85) at immediate term and-4.23 (95% CI, -7.83 to-0.63).

Acupressure (+ acupoint stimulation + CT) versus conventional therapy (CT)

One RCT [72] (51 subjects) was found regarding this comparison, which included participants with both sub-acute LBP and CNSLBP. A statistically significant reduction at immediate term was found between groups with a MD of-0.38 (95% CI, -0.41 to-0.35) in pain and-0.12 (95% CI, -0.14 to-0.10) in disability.

Side effects

Only one study reported adverse effects, which were associated with the seed placement, causing some subjects’ ears to itch (N = 7, 37%), soreness (N = 4, 21%), increased sensitivity (N = 3, 16%), sleep disturbance (N = 2, 11%), and discomfort (N = 4, 21%) [68]. This discomfort usually appeared on days 1–2 and gradually disappeared.

Summary

Two large studies showed that there was moderate evidence that acupressure could be more effective than physical therapy for pain and disability associated with CLBP in the immediate and intermediate term.

### Cupping in NP

We identified 5 RCTs (241 subjects) for cupping therapy, all of which were of fair quality and had small sample sizes [73,74,77,82,95]. Almost all of the trials principally targeted patients with chronic non-specific neck pain (CNSNP).

Cupping versus waitlist

**Fig 24:**
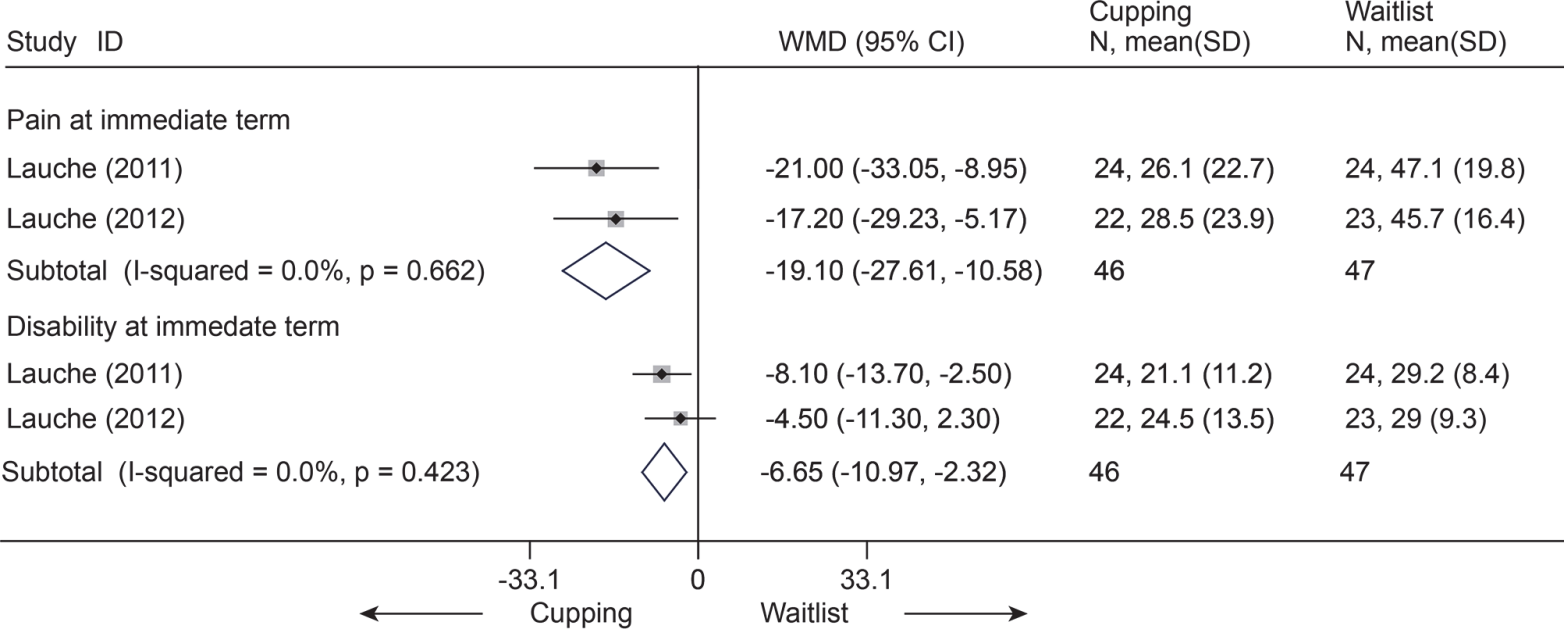
Meta-Analysis of Cupping versus Waitlist for CNP in Pain (VAS 100) and Disability (NDI 100). Fixed-effects model was used; NDI, neck disability index; CI, confidence interval; CNP, chronic neck pain; SD, standard deviation; WMD, weighted mean difference.

Two RCTs (n = 93) were identified [74,95]. After data were quantified, we found that cupping was significantly more effective than waitlist for pain (VAS 100 mm, MD, -19.10 (-27.61, -10.58)) and disability (Neck Disability Index (NDI) 100, MD, -6.65 (-10.97, -2.32)) (Fig. 24).

Cupping versus standard medical care

One trial (n = 48) was found that compared cupping with standard medical care (SMC) [77]. The experimental group experienced significantly superior reductions in pain—(NRS 0–10 cm, MD, 1.72 [-2.74, -0.70], p = 0.0009) (S5 Table), improvement in disability (NDI 0–100 points, MD, -5.78 [-10.80, -0.76], p = 0.025).

Cupping versus heating pad application

One RCT [82] (n = 40) was identified. It was reported that cupping was superior in reducing pain at 1 week (MD, -36.30 [-46.48, -26.12]) and 1 month post-treatment (MD, -21.55 [-34.92, -8.18]) on NRS 0–100 points, and improving disability at 1 week (MD, -7.69 [-13.68, -1.70]) and 1 month (MD, -10.44 [-15.48, -5.40]) post-treatment on NDI 0–100 points(S5 Table).

Cupping versus progressive muscle relaxation

One trial [73] (n = 61) was found. However, no significant differences were reported in pain (VAS 100 mm, MD, -0.16 [-13.90, 13.55], p = 0.98) or disability (NDI 0–50 points, MD, -2.18 [-4.56, -0.21], p = 0.07).

Side effects

Three trials found some adverse events, which were minor and transient [73,74,82].

Summary

Two small (number per group < 40) studies showed that cupping could be more effective than waitlist with respect to pain and disability in the immediate term for CNP (moderate evidence).

Cupping in LBP

Six RCTs pertaining to cupping therapy were identified [76,78–81,83]. Four studies were in Chinese which compared cupping with medications, and all these studies were of poor quality [78–81]. The other two fair-quality studies were in English.

Cupping versus medications

**Fig 25:**
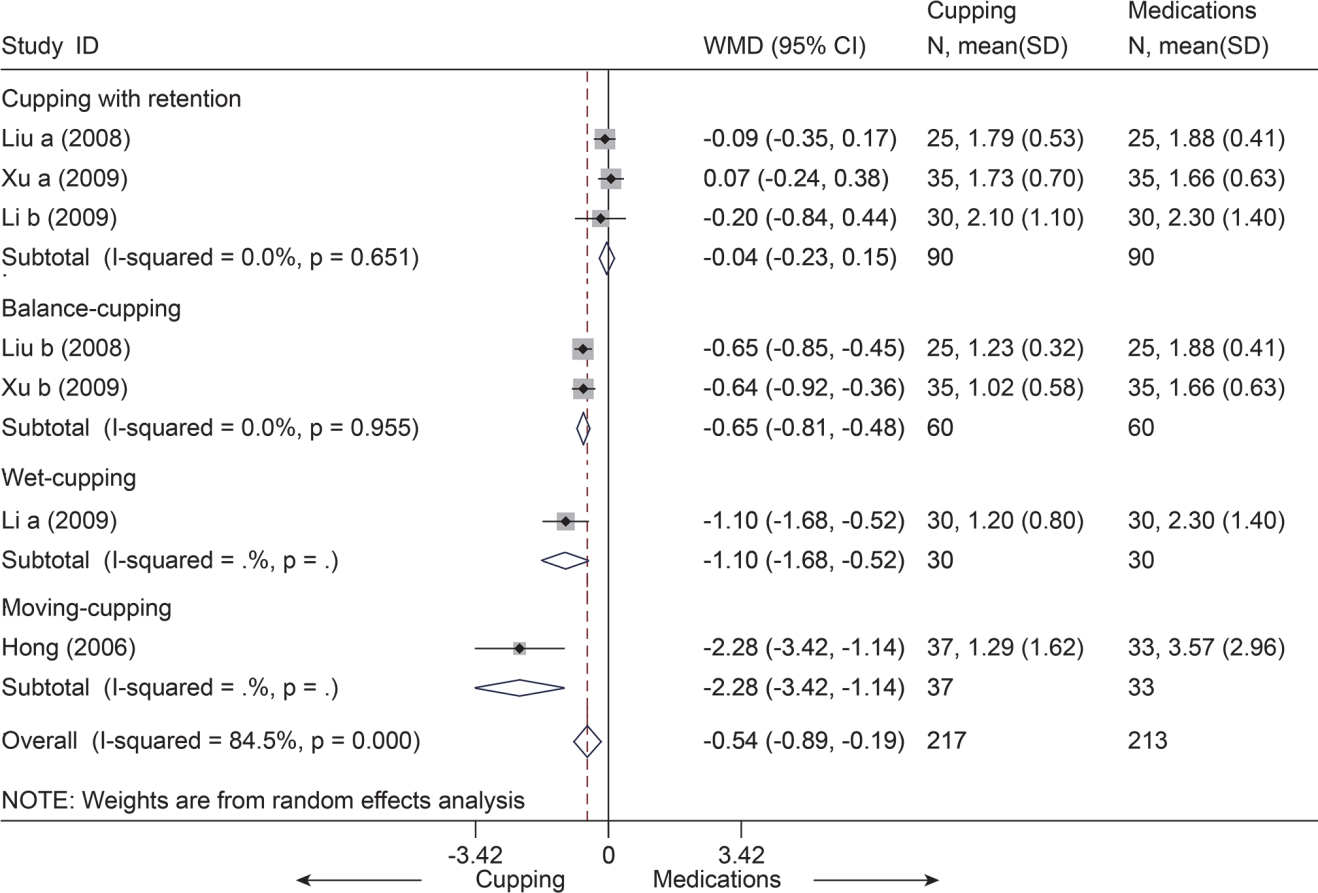
Meta-Analysis and Subgroup-Analysis of Cupping versus Medications for CLBP in Pain on VAS 10 cm. CI, confidence interval; CLBP, chronic low back pain; SD, standard deviation; WMD, weighted mean difference.

**Fig 26:**
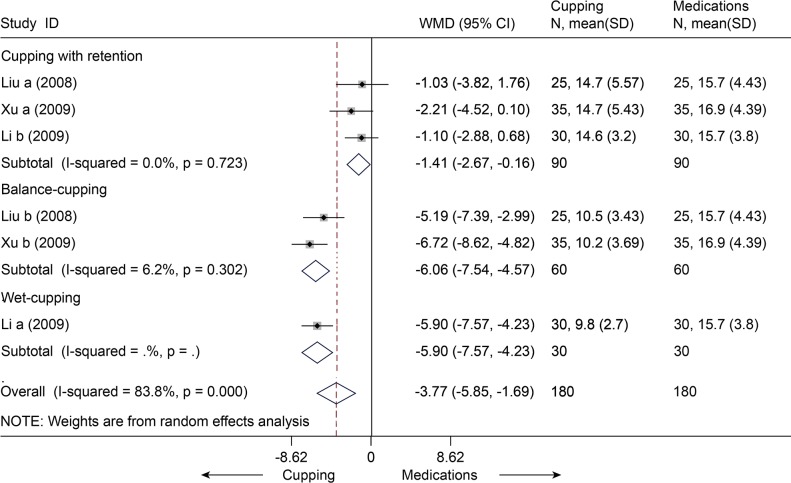
Meta-Analysis and Subgroup-Analysis of Cupping versus Medications for CLBP in Disability on ODI 50. ODI, oswestry disability index; CI, confidence interval; CLBP, chronic low back pain; SD, standard deviation; WMD, weighted mean difference.

Four studies [78–81] with seven trials (n = 430) compared cupping and medications (e.g. NSAID) for the outcome of pain or disability scores. With respect to pain, all the seven trials [78–81] were pooled in the random-effects model (Fig. 25), and the MD was-0.54 (95% CI, -0.89 to-0.19). That is, the mean pain scores were 0.54 units on the VAS (10 cm) scales significantly lower in the cupping groups than in the medications groups. However, this was highly heterogeneous across studies (I^2^ = 81.7%). No evidence of publication bias was found (Egger test, P = 0.138). Meta-regression identified that the source of heterogeneity was the types of the cupping (Adjusted R-squared = 96.34%). Subgroup analysis was performed by the types of the cupping. We found statistical significance between cupping (balance\moving\wet) and medications, but no difference about cupping with retention. With respect to disability, three studies [78–80] with six trials (n = 360) were pooled with a MD of-3.77 (95% CI, -5.85 to-1.69, random, I^2^ = 83.8%) (Fig. 26). This indicated that cupping could decrease mean disability score of 3.77 units on ODI (0–50) scale. The Egger test suggested no evidence of publication bias (P = 0.166). Meta-regression suggest that the types of the cupping was the source of heterogeneity (Adjusted R-squared = 100.00%). We performed subgroup analysis according to the types of the cupping. Significant differences were still found between cupping (retention\balance\wet) and medications.

Cupping versus waitlist

One RCT (n = 32) was identified [76]. There were no significant differences between the two groups in pain and disability (S5 Table).

Cupping versus usual care

Usual care indicated that participants received no study-related care—just the care, if any, that they and their physicians chose: mostly massage and physical therapy visits and continued use of medications (mostly NSAIDs). One trial (n = 98) was found [83]. Upon a 3-month follow-up, the report noted that the cupping showed significant differences in pain (Present Pain Itensity 0–5 points, MD, -2.20 [-2.60, -1.70], p = 0.01) and disability (ODI 0–60 points, MD, -15.0 [-18.8, -11.2], p = 0.01).

Side effects

Three individuals experienced fainting (vaso-vagal shock) [83]. However, no adverse events were found in another study [76].

Summary

Several small and lower-quality studies showed that there was low evidence with small clinical significance that cupping was more effective than medications (e.g. NSAID) in reducing pain and disability at immediate term for CLBP. One large study showed that there was moderate evidence with a large clinical significance that cupping was more effective than usual care in treating pain and disability in the short term for CLBP.

### Gua sha in CNP

There were only two studies (n = 69) that had researched the efficacy of gua sha for NP [84,85], both of which were fair-quality RCTs.

Gua sha versus waitlist/no treatment

There was only one RCT (n = 21) found [84]. Seven days after treatment, significant differences favoring gua sha were found for CNP with respect to pain (VAS 10 cm, MD-1.6 (-3.0, -0.1)) (S5 Table). The study did not report disability.

Gua sha versus thermal therapy

Likewise, only one trial (n = 48) [85] was identified. The pain (VAS 100 mm) showed a statistically and clinically significant reduction in the gua sha group compared with the control group during a 1-week period (MD, -29.9 (-43.3, -16.6)). These differences were also observed for disability (NDI, MD-8.5 (-13.6, -3.5)).

Side effects

One trial [85] reported that there may be petechiae, slight muscle aches, and soreness in the application area, with none of these effects being serious.

Summary

Small studies showed that there was low evidence with medium to large clinical importance that gua sha was more effective than waitlist and thermal therapy for pain in the immediate term for CNP.

### Gua sha in CLBP

Only one study was identified [84].

Gua sha versus waitlist/no treatment

There was a fair-quality RCT (n = 21) found [84]. At a 7-day follow-up, with respect to pain (VAS 10 cm), significant between-group differences favoring gua sha were observed (MD, -1.1 (-2.0, -0.2)). Nevertheless, the study did not report on disability.

Side effects

None reported.

Summary

A very small study provided low evidence with medium clinical importance that gua sha was more effective than waitlist in treating pain in the immediate term for CNP.

### Qigong in CNP

There were three fair-quality RCTs associated with the use of qigong therapy for NP [86–88].

Qigong versus waitlist/no treatment

**Fig 27:**
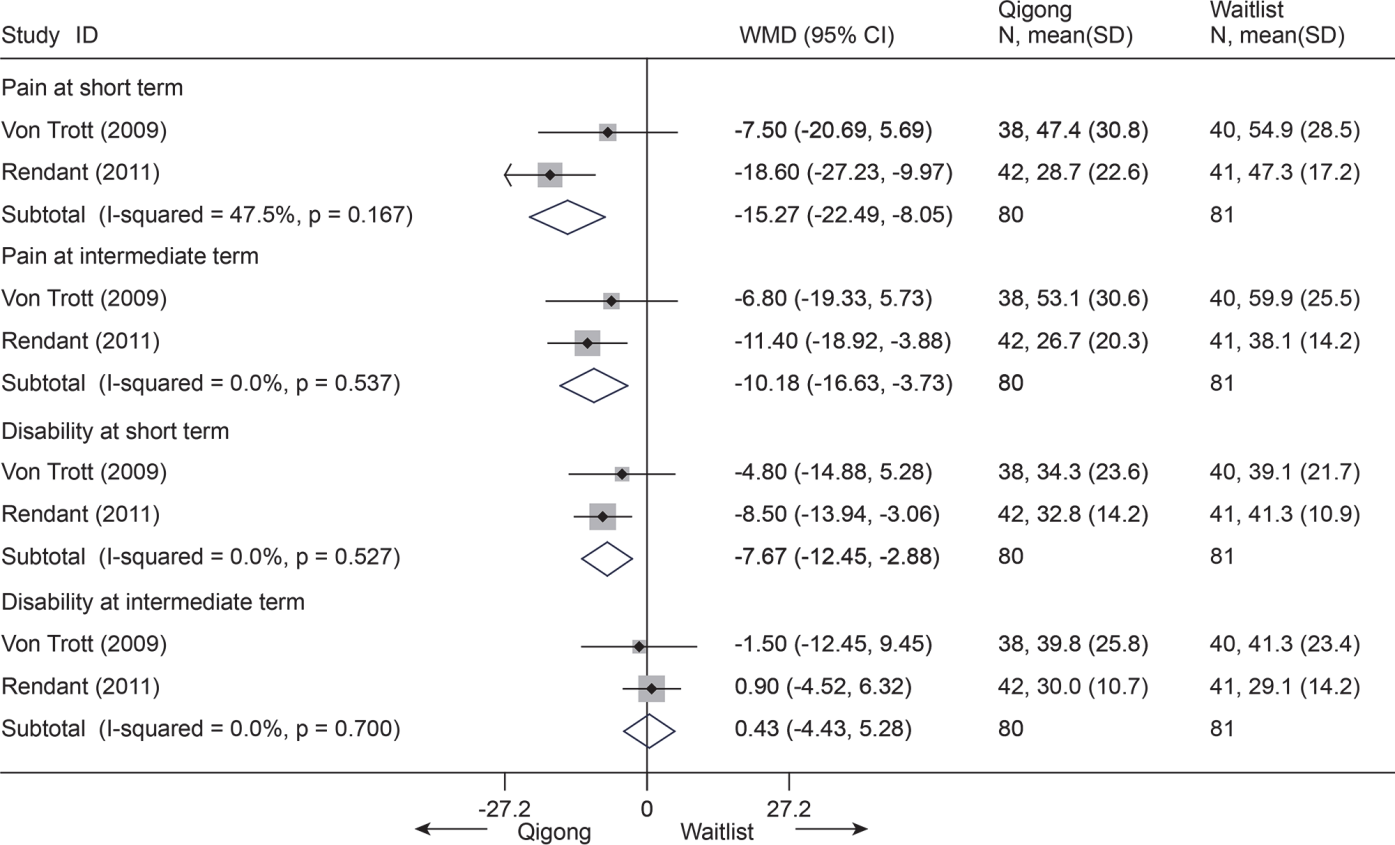
Meta-Analysis of Qigong versus Waitlist for CNP in Pain (VAS 100) and Disability (NDI 100). Fixed-effects model was used; NDI, neck disability index; CI, confidence interval; CNP, chronic neck pain; SD, standard deviation; WMD, weighted mean difference.

There were 2 RCTs (n = 161) found [86,87]. Differences were evident between these two groups in pain (VAS 100mm) at 3 and 6 months post-intervention, except for the disability outcome (NDI 100) (Fig. 27).

Qigong versus exercise

**Fig 28:**
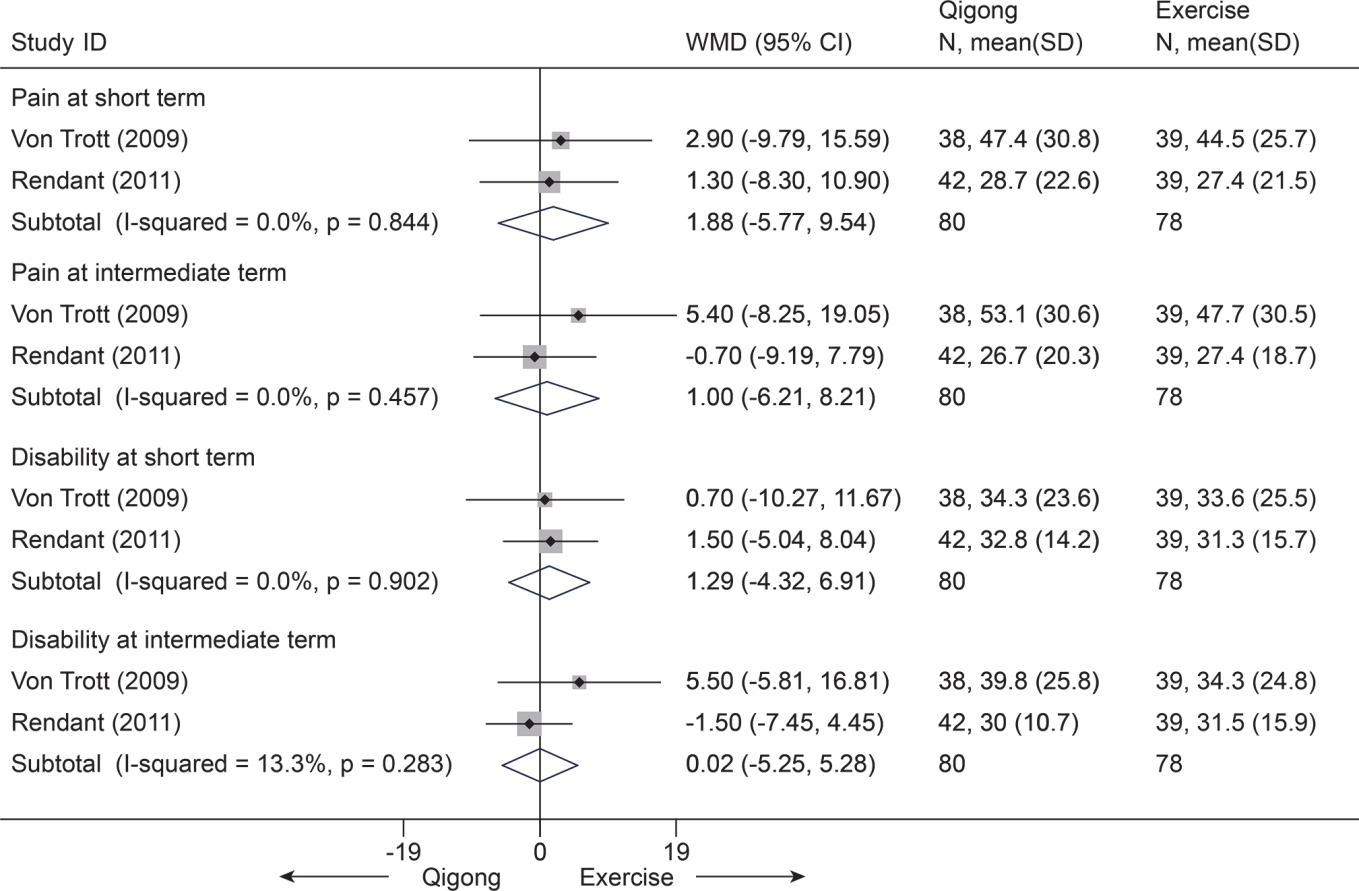
Meta-Analysis of Qigong versus Exercise for CNP in Pain (VAS 100) and Disability (NDI 100). Fixed-effects model was used; NDI, neck disability index; CI, confidence interval; CNP, chronic neck pain; SD, standard deviation; WMD, weighted mean difference.

Three trials (n = 280) were identified. The first trial [88] showed that there were no differences about pain relief (VAS 100 mm, IQR, 41 (2–81) vs 26 (0–84), p>0.05) and disability improvement (NDI 0–70 points, IQR, 24 (2–68) vs 17 (2–52), p>0.05) at immediate term. Nevertheless, the other two trials (158 individuals) [86,87] showed no differences in pain at short term (MD, 1.88 [-5.78, 9.54], p = 0.63) and intermediate term (MD, 1.00 [-6.21, 8.21], p = 0.79) on VAS 100 mm, and also in disability improvement at short term (MD, 1.29 [-4.33, 6.91], 0.65) and intermediate term (MD, 0.02 [-5.25, 5.28], p = 1.00) on Neck Pain and Disability Scale 0–100 points between groups (Fig. 28).

Side effects

One study showed that 5 side effects were reported by 4 patients (2 with nausea, 2 with aching muscles, and 1 with muscle tension) [87]. The last study reported side effects in 19 patients (muscle soreness (15), myogelosis (12), vertigo (10), and other pain (4)) [86]. These side effects were not serious and also occurred in the exercise group.

Summary

Two small studies showed that qigong may be superior compared with waitlist for CNP sufferers (moderate evidence), but no differences were found compared with other exercises.

### Qigong in LBP

There were no RCTs identified.

### Tai chi in NP

There were no RCTs identified.

### Tai chi in LBP

Only one trial of tai chi for LBP was found. This was a fair-quality RCT with a large sample size (n = 160) [96]. All of the subjects experienced CNSLBP.

Tai chi versus waitlist

Compared with waitlist, tai chi reduced pain (NRS 0–10) and disability (Pain Disability Index, 0–70) by 1.3 points (95% CI, 0.7 to 1.9) and 5.7 points (95%, 1.8 to 9.6) immediately post-treatment, respectively.

Side effects

None reported.

Summary

A large study showed that tai chi could be more effective than waitlist for CLBP (moderate evidence). Because the findings were derived from only one RCT, the validity of the evidence above should be treated cautiously.

### Chinese Herbal Medicine in NP

**Fig 29:**
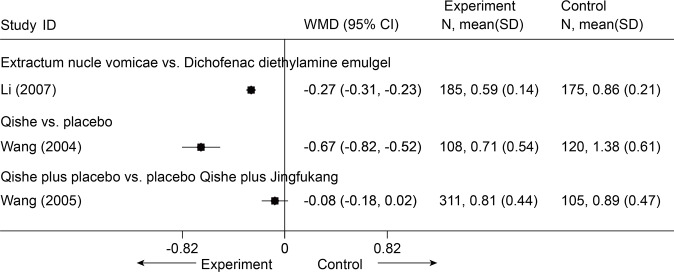
Meta-Analysis of Chinese Herbal Medicine for CNP in Pain (NRS 3). NRS, numerical rating scale; CI, confidence interval; CNP, chronic neck pain; SD, standard deviation; WMD, weighted mean difference.

There were 3 studies identified. One study[90] retrieved from database, and two other studies (H. Wang, personal communication, 2004; H. Wang, personal communication, 2005) were identified through consultation with experts. All these studies were in Chinese and 2 of these studies were unpublished. All 3 included trials assessed pain severity. Functional status was not assessed in all 3 trials. There were no continuous outcomes reported. The primary outcomes were expressed with an ordinal scale. So we re-expressed the result as continuous data using NRS (0–3 points, the lower indicates the better) (Fig. 29).

Extractum nucis vomicae vs. diclofenac diethylamine emulgel

One trial[90] (n = 360) showed that the experimental group exhibited a significant difference in pain (p < 0.001) associated with CNP compared with the control group in the immediate term (MD = -0.27, 95% CI-0.31 to-0.23).

Qishe vs. placebo

One RCT (n = 240) (H. Wang, personal communication, 2004) showed that Compound Qishe Tablets outperformed a placebo with respect to pain associated with CNP at 2 months (MD = -0.67, 95% CI-0.82 to-0.52).

Qishe plus placebo Jingfukang vs. placebo Qishe plus Jingfukang

One trial (n = 440) (H. Wang, personal communication, 2005) showed no significant difference between groups with respect to pain associated with CNP in a 2-month follow-up (MD = -0.08, 95% CI-0.18 to 0.02).

Side effects

All of the trials included reported side effects, such as watery stool, abdominal pain, stomachache, pruritus, reddish skin, and small blisters on the skin. However, no life-threatening complications were reported in any of the trials.

Summary

A large trial showed that there was moderate evidence with large clinical importance that the Chinese herbal medicine Qishe may be more effective in treating pain for CNP in the short term than placebo. In view of the limitations of the included trials, such as a lack of allocation concealment, blinding, and intention-to-treat analysis, the results above should be treated with caution.

### Chinese Herbal Medicine in LBP

No RCTs or SRs were identified.

### Chinese Manipulation in CNP

Three studies (n = 396) were identified[91–93], in which two were published in Chinese[92,93].

Chinese manipulation (CM) versus Chinese traditional massage (CTM)

**Fig 30:**
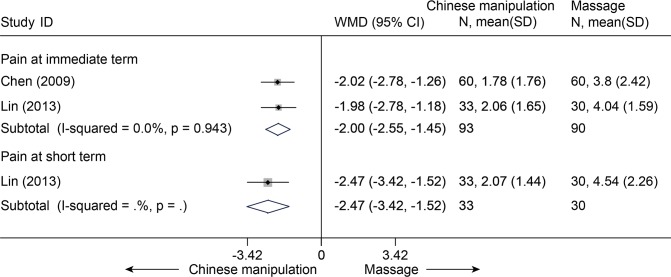
Meta-Analysis of Chinese Manipulation versus Chinese Massage for CNP in Pain (NRS 10). Fixed-effects model was used; NRS, numerical rating scale; CI, confidence interval; CNP, chronic neck pain; SD, standard deviation; WMD, weighted mean difference.

Two studies (n = 183) were identified [91,92]. Both of these studies showed that CM was more effective than CTM for immediate-term pain relief (NRS 10 cm, MD, –2.00 (–2.55, –1.45)) (Fig. 30). One study reported that CM was more effective for short-term pain relief (p < 0.001) and for immediate-term (p < 0.001) and short-term disability improvement (NPQ 40) (p < 0.001)[91].

Chinese manipulation versus cervical traction

There was only one study [93] (213 subjects, higher quality) that reported pain. This study showed that CM was more effective in pain relief immediately post-intervention (VAS 10 cm, MD, –1.06 (–1.37, –0.75)). Disability was not reported.

Side effects

Two studies mentioned adverse events; none were observed in either study [91,92].

Summary

Two small studies showed that there is moderate evidence with large clinical importance that Chinese manipulation is more effective than traditional Chinese massage in relieving pain associated with CNP in the immediate term. Additionally, one large study also showed this favorable effect compared with traction.

### Chinese Manipulation in LBP

No RCTs were found.

### Moxibustion in NP

No RCTs were found.

### Moxibustion in LBP

No study reported directly about pain and function, which were of principal concern in our study.

Summary

There was insufficient evidence supporting moxibustion because the outcomes reported in the review were not directly about pain and function.

### Tuina in NP

No RCTs were found.

### Tuina in LBP

No studies directly compared tuina alone with one of the other treatments; however, tuina was combined with other traditional Chinese therapies (tuina-focused integrative Chinese medical therapy, TICMT), and TICMT was then compared with other traditional Chinese therapies. And no trial reported pain and disability.

Side effects

None reported.

Summary

There was insufficient evidence regarding tuina because there were no direct comparisons between tuina alone and other treatments.

## Discussion

There were a total of 10 methods of treatment for NP or LBP. The included studies concentrated primarily on three therapies (i.e., acupuncture, acupressure, and cupping), with an emphasis on acupuncture, whereas the studies on the other seven treatments were fewer in number. This finding might suggest that these treatments were not well known in western countries and were not standard of care.

All the treatments showed positive effectiveness compared with baseline measurements. Compared with sham acupuncture (SA), acupuncture may be more effective in reducing pain and disability in the immediate and one-month term for individuals with CNP, which was consistent with the former SRs [97,98]. However, inconsistency was found with another SR, which reported no significant differences between these two groups [9]. We determined that this inconsistency arises from three subsequent RCTs with larger sample sizes and positive results favoring acupuncture. Similarly, these differences in immediate-term and short-term outcomes about pain also existed for individuals with CLBP, but no difference about disability. Moreover, the difference was evident for acute LBP in the immediate term. Nevertheless, the difference in clinical importance between acupuncture and SA was small. The SA group was used to estimate the specificity of the acupuncture points and of the technique itself. However, a standardized SA has not yet been established. Therefore, it has been a challenge for researchers to choose the correct acupoints for the SA group. As a result, the effect of true acupuncture will be underestimated. Thus, various degrees of efficacy were observed in different studies. For example, the trial by Vas et al. (2012) [49] reported the effects of acupuncture in subjects with acute nonspecific LBP. In its protocol [99], the authors had chosen some acupoints for SA groups, such as Neiguan (PC-6) and Kongzui (LU-6), which were proven to be effective in previous studies. In 1989, Geng [100] found that 51 patients with acute LBP (pain duration < 10 days) were treated only by PC-6. Thirty eight patients got complete pain relief after just one treatment session. Meanwhile, a case report by Xu [101] reported that the acute LBP of a 37-year-old man was relieved by only using LU-6. A review [102] reported that up to forty one acupoints had been found to be efficacious for individuals with acute LBP, including the PC-6 and LU-6. Thus, it is particularly important to establish a unified standard with which to choose the correct acupoints for SA. We found that acupressure may be more effective than physical therapy but sham-acupressure for CLBP. Additionally, small studies (number per group < 40) showed that CNP patients treated with cupping experienced more pain relief than waitlist controls with CNP and that cupping was more effective at reducing CLBP than medications (e.g. NSAID). The studies associated with these two treatments were relatively higher in number and quality, such as the trial on acupressure by Hsieh [71]. We found that the Chinese herbal medicine Qishe was more effective for reducing CNP in the short term compared with placebo. Small studies showed that gua sha may be more effective than thermal therapy or waitlist control. However, the sample sizes were very small (number per group < 10), and it was difficult to draw any conclusions. Chinese manipulation may be effective for CNP, with results that are consistent with the results of studies on conventional Western manipulation [103,104]. As specific types of mind-body exercises, tai chi and qigong show superior effects for CNP or CLBP compared with effects observed in the waitlist control groups; this finding is consistent with the previous reviews regarding other exercises [105,106], especially yoga, which is also a mind-body exercise. However, the number of studies was so sparse that we were unable to draw conclusions regarding these two treatments. There was insufficient evidence supporting tuina and moxibustion because there were no direct comparisons between tuina and other treatments, and no direct outcomes on pain or disability were reported for moxibustion.

With regard to clinical importance, we found that most of the study sizes were small, others were medium, and only a few were large. This result may be due to the small clinical significances between these treatments themselves, as well as to the small sample sizes, which may give rise to random errors. With respect to the strength of evidence, we found that most of the comparisons had low strengths of evidence, others were moderate, and none were high. We believe that the causes for this variation mainly derive from the following reasons: 1) the quality of the individual studies was lowered because most of the trials failed to include a blinded control group, which was due to the characteristics of the interventions themselves (e.g., tai chi, qigong, tuina, gua sha, cupping, and Chinese manipulation); 2) the sample sizes of some trials were smaller than 40 individuals in each arm, which would lower the strength of the evidence directly by one level; and 3) inconsistency (i.e., different directions of effects or high heterogeneity (I^2^ ≥ 50%)) exists between trials. Obviously, inconsistency of study results in a meta-analysis reduces the confidence of recommendations regarding treatment [107].

Our review has several main limitations, which were due to the studies included. First, we found that the number of studies was small (even zero for some treatments, such as qigong for NP). Thus, further studies in these areas are warranted. Second, the strength of the evidence was low or moderate rather than high, which means that the results may change through further research. Third, the analysis was limited to studies published in English and Chinese when numerous studies had been carried out in various countries. However, the outcome measures in Chinese studies were usually cure rates or efficacy rates of overall improvements, and we were unable to obtain data on pain and disability. Although there were usually larger sample sizes in Chinese studies compared with English studies, there were definite flaws in the designs of Chinese studies and higher risks of bias, such as a lack of allocation concealment[78–81,90]. Forth, the studies on moxibustion did not report the outcomes we pre-established, and the studies on tuina even compared tuina with itself. As a consequence, we were unable to obtain any direct findings regarding tuina and moxibustion.

The clinical heterogeneities of some of our meta-analyses might limit the translations of our results. First, the selections of acupoints for LBP varied from study to study, and even from patient to patient. However, these heterogeneities were inevitable because the selections of acupoints should be individualized and disease-specific according to TCM theory, but the meridians of these points were homogeneous in some extent. Second, the heterogeneities might be due to different population, such as the wide range of ages (17–90 years) and different peoples from different countries. Third, the underlying differential TCM-diagnoses in patients suffering from NP or LBP might be responsible for the heterogeneity to some extent. Lastly, the variances of treatments methods in different trials might also contribute to the heterogeneity, such as the numbers of treatment sessions or durations, and the frequencies or intervals of treatments. For these reasons, a standardized treatment for each group of a sub-diagnosis is needed in order to gain a higher homogeneity for future studies.

## Conclusions

Although there were many deficiencies in our review, the major strength of our study was the coverage of all aspects of TCM in individuals with NP or LBP. Our review demonstrated that acupuncture, acupressure, and cupping could be efficacious in pain and disability for CNP or CLBP in the immediate term. Some treatments, such as gua sha, tai chi, qigong, and manipulation, showed fair effects, but we were unable to draw definite conclusions, and further studies on these treatments are needed. Currently, we do not know much about the efficacy of tuina and moxibustion because there was no direct evidence in any of the studies evaluated. No serious or life-threatening adverse effects were found, indicating that TCM treatments are safe for patients. Nevertheless, considering the sparse studies and inferior methodological quality of the individual trials, we propose that TCM could be used as a supplement to occupational therapies for people with CNP or CLBP. Moreover, sham therapies need to be designed to improve the strength of evidence. The studies in Chinese should report the outcomes of pain and disability definitely, which will benefit the evaluations of these outcomes used internationally. In summary, many more studies with higher quality and longer-term follow-ups are warranted. For future studies, a standardized treatment for each group of a sub-diagnosis is needed in order to gain a higher homogeneity.

## Supporting Information

S1 PRISMA Checklist. (DOC)

S1 Table Definitions of Included Interventions. (DOC)

S2 Table Search Strategy for Finding Evidence on Interventions for Low Back Pain and Neck Pain in Pubmed. (DOC)

S3 Table Study Quality and Risk of Bias. (DOC)

S4 Table Strength of Evidence Grades and Definitions. (DOC)

S5 Table Meta-analyses and sensitivity-analyses and subgroup-analyses of pain and disability. (DOC)

S6 Table The Basic Characteristics of Individual Studies Included. (DOCX)

S7 Table The Risk of Bias and Quality of Individual Studies Included. (DOCX)

S8 Table Strength of Evidence and Clinical significance (Major Outcomes: Pain Intensity, Disability). (DOC)
