# Supplementary material for: Traditional Chinese Medicine for Neck Pain and Low Back Pain: A Systematic Review and Meta-Analysis
Source: PLoS One. 2015 Feb 24;10(2):e0117146. doi: 10.1371/journal.pone.0117146 (PMC4339195; doi:10.1371/journal.pone.0117146)
Supplement: S1 Table — (DOC) [file pone.0117146.s002.doc]

# S1 Table. Definitions of Included Interventions.

| **Intervention** | **Definition** |
| --- | --- |
| **Acupuncture** | Acupuncture is a therapeutic method that prevents and cures disease by means of penetrating needles into specific acupoints on the human body, guided by the theory of meridians and acupoints of traditional Chinese medicine . There are various types of acupuncture styles, such as manual stimulation of the needles as well as electricity or heat stimulation of the needles. |
| **Acupressure** | Acupressure uses the application of fingers and pressure to stimulate specific acupoints on the human body . This noninvasive therapy was originally developed from TCM, which focuses on the balance of yin and yang and maintains the function of vital organs through circulation of blood and energy (chi) in the body. |
| **Cupping** | Cupping is an ancient technique that aims to induce local congestion and blood stasis for healing. It works according to the principles of acupoints and meridians. The cups are made in different sizes and come in bamboo, plastic, glass or porcelain. The treatment consists of creating a vacuum by burning a taper inside a cup and quickly placing the cup over the selected area. TCM holds that the resulting suction effect invigorates [blood](http://www.shen-nong.com/eng/principles/blood.html)and [qi](http://www.shen-nong.com/eng/principles/qi.html) flows, unblocks the meridians, dispels dampness and cold, and eases swelling and pain. It is commonly applied in so-called bi syndromes (bi zheng), such as musculoskeletal pains . |
| **Gua sha** | Under the guidance of the basic theories of traditional Chinese medicine (TCM), gua sha is a technique of scraping the surface of body at targeted locations using specific scraping instruments to prevent disease . After scraping, ecchymosis and petechia is evident at the local skin site, which are called “sha;” it is believed that poisons in the inner body have been extracted in the shape of the sha . Gua sha is technique used for pain relief, muscular tension, and spasms. Its main mechanisms are, on one hand, to strengthen regional circulation and evaluate the temperature of local tissue and, on the other hand, to boost the pain threshold of regional tissue. |
| **Qigong** | Qigong , a therapeutic Chinese practice that has been used for a thousand years to guard against and treat disease, refers to a mind- and body-training method that focuses on breathing adjustment, physical activity modulation, and willing adjustment. Qigong is part of traditional Chinese medicine and follows the principles of regulating “qi” (energy), which is assumed to harmonize mind and body to reduce pain . Five-animal exercise is a subgroup of qigong, which imitates the movements and expressions of five animals and was an important regimen in ancient times. TCM therapists who practice acupuncture and moxibustion usually enhance the curative effects of these other therapies by means of qigong exercise. |
| **Tai chi** | Tai chi is the name for an immemorial Chinese philosophy and conveys the ideas of the supreme, extreme, absolute, and unique . The symbol of tai chi conveys the two opposing forces, yin and yang,which are not absolute and are capable of changing each other’s direction. Tai chi quan is a perfect combination of Chinese national dialectical theoretical thinking and martial arts, art, guiding methodology, and traditional Chinese medicine. The core concept consists of Confucianism, the tai chi of Taoism, and the philosophy of yin and yang . Tai chi as a high-level expression of the human body has multiple functions, which include taking care of one’s temperament, building one’s body, and the art of attack and defense. |
| **Chinese herbal medicine** | In TCM, herbal therapies, which have been used for centuries in China, are generally formula-based, and single herbs are rarely used . TCM holds that every medicinal substance has its strengths and its shortcomings, and each ingredient in the formula should be carefully balanced in quality and quantity, in order to accentuate its efficacy while reducing side effects . The herbs are not simply added in a cumulative fashion but are combined according to particular principles. First, through a unique diagnostic process, physicians discern the subtle patterns according to the symptoms of the individual, which then guides them to determine therapeutic strategies and to design or select the appropriate formulae. |
| **Chinese manipulation** | Chinese manipulation is an important part of TCM. It is sorted into different schools according to the names of their initiators or specific techniques . In brief, according to the differences that exist in mechanisms and techniques, it can be classified into three approaches: rotation, traction or a combination . Despite their different names or styles, Chinese manipulation disciplines share the same principles: to reconstruct the balance of the musculoskeletal system through relaxing the muscles and rectifying abnormalities in the relationships between joints . Chinese manipulation, which is based on the meridians and acupoints of TCM , differs from Western manipulation in theory and practice. It is mainly composed of two parts: relaxation of the soft tissues and bone setting. Universally, massage techniques and acupressure are used for relaxing the soft tissue and dredging the channel, whereas bone setting is used to restore joints . |
| **Moxibustion** | Moxibustion is a TCM therapy that treats of diseases by means of the heat generated by burning an herbal praeparatum mainly containing mugwort . Moxibustion’s purported effectiveness is based on opposing inflammation or inflammatory reactions in the human body . Various methods of moxibustion therapy for neck or back pain have almost the same mechanisms, which may improve regional blood circulation, eliminate inflammatory reactions and the edema of nerve roots. Furthermore, the heat that follows mugwort burning may change the local microcirculation of the vertebra lumbalis or cervicalis. Acupoints in human body have two conditions: activation and rest. The acupoints on the body surface are activated and sensitized when individuals get sick. The areas of sensitivity, which are called heat-sensitive acupoints, are sensitive to thermal stimulus. These sensitive areas, on one hand, could reflect the pathological phenomena of diseases; on the other hand, they may be effective stimulus sites. |
| **Tuina** | As a part of traditional Chinese medicine using manual therapy, tuina also emphasizes the meridians and acupoints as well as some anatomy and physiology. Tuina is mainly composed of two components: soft tissue manipulation and backbone manipulation. The soft tissue techniques, which are analogous to massage, include stroking, kneading, and drumming. In contrast, the backbone manipulation is similar to mobilization and other regulation techniques. These techniques use manual operation procedures without thrust, which can maintain the range of the spine during physiological activity. Whereas, they also include procedures with thrust, which can make the spine exceed its physiological and anatomical range. |
