# Supplementary material for: Traditional Chinese Medicine for Neck Pain and Low Back Pain: A Systematic Review and Meta-Analysis
Source: PLoS One. 2015 Feb 24;10(2):e0117146. doi: 10.1371/journal.pone.0117146 (PMC4339195; doi:10.1371/journal.pone.0117146)
Supplement: S2 Table — (DOC) [file pone.0117146.s003.doc]

**S2 Table. Search Strategy for Finding Evidence on Interventions for Low Back Pain and Neck Pain in Pubmed.**

| **Steps** | **Fields** | **Strings** |
| --- | --- | --- |
|  | Traditional Chinese medicine [tiab] | Interventions |
|  | Alternative medicine [tiab] |  |
|  | Complementary medicine [tiab] |  |
|  | Acupuncture [tiab] |  |
|  | Electroacupuncture [tiab] |  |
|  | Acupoints [tiab] |  |
|  | Auricular needle [tiab] |  |
|  | Auriculotherapy [tiab] |  |
|  | Acupressure [tiab] |  |
|  | Cupping [tiab] |  |
|  | Chinese herbal medicine [tiab] |  |
|  | Moxibustion [tiab] |  |
|  | Gua sha OR Guasha [tiab] |  |
|  | Scraping [tiab] |  |
|  | Chinese manipulation [tiab] |  |
|  | Tuina [tiab] |  |
|  | Tai chi OR Taichi OR Tai ji [tiab] |  |
|  | Qigong [tiab] |  |
|  | OR/ 1-18 |  |
|  | (Disc OR Disk) AND degeneration [tiab] | Participants |
|  | Intervertebral disc degeneration [mesh] |  |
|  | Degenerative AND (Disk OR disc) [tiab] |  |
|  | Low back pain [tiab] |  |
|  | Lower back pain [tiab] |  |
|  | Back pain OR lumbago [tiab] |  |
|  | Lumbodorsal OR dorsalgia [tiab] |  |
|  | Discogenic pain [tiab] |  |
|  | Neck pain OR cervical pain [tiab] |  |
|  | Cervicodynia OR cervicogenic pain [tiab] |  |
|  | Disc herniation OR disc prolapse [tiab] |  |
|  | Disc protrusion [tiab] |  |
|  | Radicular pain OR root pain [tiab] |  |
|  | Cervical spondylosis [tiab] |  |
|  | Cervical spondylopathy [tiab] |  |
|  | Cervical sydrome [tiab] |  |
|  | OR/20-35 |  |
|  | Randomized controlled trial [pt] | Filter |
|  | Clinical trial [pt] |  |
|  | Controlled clinical trial [pt] |  |
|  | Randomized OR randomly OR random [tiab] |  |
|  | Blind OR blinded OR blinding [tiab] |  |
|  | Sham OR placebos OR placebo [tiab] |  |
|  | Scientific integrity review [tiab] |  |
|  | Systematic review [pt] |  |
|  | Systematic review [tiab] |  |
|  | Review literature as Topic |  |
|  | Evidence-based medicine [tiab] |  |
|  | Guideline OR practice guideline [tiab] |  |
|  | Meta-analysis [tiab] |  |
|  | Meta-analysis [pt] |  |
|  | OR/37-50 |  |
|  | English [la] |  |
|  | “1996/01/01” to present^[dp] |  |
|  | AND/19,36,51,56,57 |  |

*[mesh], [tiab], [pt], [dp], [la]* search field used in pubmed, abbreviations of Subject headings, title/abstract, publication type, publication date, and language, respectively. ^ present is the last time the databases were run. The strategy above can also be run in OVID and CDSR.
