# Supplementary material for: Traditional Chinese Medicine for Neck Pain and Low Back Pain: A Systematic Review and Meta-Analysis
Source: PLoS One. 2015 Feb 24;10(2):e0117146. doi: 10.1371/journal.pone.0117146 (PMC4339195; doi:10.1371/journal.pone.0117146)
Supplement: S3 Table — (DOC) [file pone.0117146.s004.doc]

**S3 Table. Study Quality and Risk of Bias**

| **Quality score^** | **Study Quality** | **Risk of Bias** |
| --- | --- | --- |
| **0 ≤N ＜ 2** | Poor | High |
| **2≤ N ＜4** | Fair | Medium |
| **N = 4** | Good | Low |

^ Number of “Yes” on 4 domains; in case of a single study, N is a whole number (0, 1, 2, 3, 4); in case of multiple studies, N is a mean number which may be whole number or fraction
