# Supplementary material for: Traditional Chinese Medicine for Neck Pain and Low Back Pain: A Systematic Review and Meta-Analysis
Source: PLoS One. 2015 Feb 24;10(2):e0117146. doi: 10.1371/journal.pone.0117146 (PMC4339195; doi:10.1371/journal.pone.0117146)
Supplement: S4 Table — (DOC) [file pone.0117146.s005.doc]

**S4 Table.** Strength of Evidence Grades and Deﬁnitions.

| **Grade** | **Deﬁnition** |
| --- | --- |
| **High** | High conﬁdence that the evidence reﬂects the true effect. Further research is very unlikely to change our conﬁdence in the estimate of effect. |
| **Moderate** | Moderate conﬁdence that the evidence reﬂects the true effect. Further research may change our conﬁdence in the estimate of effect and may change the estimate. |
| **Low** | Low conﬁdence that the evidence reﬂects the true effect. Further research is likely to change the conﬁdence in the estimate of effect and is likely to change the estimate. |
| **Insufﬁcient** | Evidence either is unavailable or does not permit a conclusion |
