# Supplementary material for: Traditional Chinese Medicine for Neck Pain and Low Back Pain: A Systematic Review and Meta-Analysis
Source: PLoS One. 2015 Feb 24;10(2):e0117146. doi: 10.1371/journal.pone.0117146 (PMC4339195; doi:10.1371/journal.pone.0117146)
Supplement: S5 Table — (DOC) [file pone.0117146.s006.doc]

**S5 Table.** Meta-analyses and sensitivity-analyses and subgroup-analyses of pain and disability.

| **Number of Studies and Participants** | **Comparison** | **Outcome ( Follow-Up Time)** | **Effect Size (95% CI)*** | **P Value**§ | **Fixed or Random model** | **I^2^ for Heterogenity (%)** |
| --- | --- | --- | --- | --- | --- | --- |
| **Acupuncture in NP** |  |  |  |  |  |  |
| 7, 428 | Acupuncture v sham acupuncture | Pain (immediate term) | -0.58 [-0.94, -0.22] MD | 0.001 | Fixed | 46.3 |
| 2, 290 |  | Pain (1 month) | -0.72 [-1.07, -0.37] MD | 0.000 | Fixed | 0 |
| 3, 319 |  | Pain (short term) | -0.32 [-0.68, 0.04] MD | 0.082 | Fixed | 0 |
| 4, 334 |  | Disability (immediate term) | -0.29 [-0.51, -0.07] SMD | 0.009 | Fixed | 0 |
| 2, 290 |  | Disability (1 month) | -0.42 [-0.66, -0.19] SMD | 0.000 | Fixed | 0 |
| 3, 305 |  | Disability (short term) | -0.37 [-0.59, -0.14] SMD | 0.001 | Fixed | 0 |
| 3, 272 | Acupuncture v sham TENS | Pain (immediate term) | 0.73 [-2.05, 3.51] MD | 0.607 | Random | 96.6 |
| 2, 149 |  | Pain (immediate term) # | -0.53 [-1.84, 0.79] MD | 0.433 | Random | 57.1 |
| 3, 227 |  | Pain (short term) | 0.45 [-0.98, 1.87] MD | 0.539 | Random | 78.6 |
| 2, 142 |  | Pain (short term) # | -0.40 [-1.07, 0.27] MD | 0.241 | Fixed | 44.6 |
| 3, 273 |  | Disability (immediate term) | 0.40 [-0.55, 1.36] SMD | 0.409 | Random | 92.2 |
| 2, 150 |  | Disability (immediate term) # | -0.07 [-0.39, 0.25] SMD | 0.686 | Fixed | 0 |
| 2, 143 |  | Disability (short term) | -0.18 [-0.51, 0.15] SMD | 0.274 | Fixed | 0 |
| 1, 108 | Acupuncture v sham laser | Pain (immediate term) | -0.69 [-1.75, 0.37] MD | 0.202 | - | - |
| 1, 108 | Acupuncture v massage | Pain (immediate term) | -1.63 [-2.68, -0.58] MD | 0.002 | - | - |
| 4, 146 | Acupuncture v medication | Pain (immediate term) | -0.57 [-1.14, -0.01] SMD | 0.048 | Random | 58.4 |
| 3, 116 |  | Pain (immediate term) # | -0.35 [-0.72, 0.01] SMD | 0.060 | Fixed | 0 |
| 2, 94 |  | Disability (immediate term) | -0.18 [-0.59, 0.23] SMD | 0.387 | Fixed | 0 |
| 2, 99 | Acupuncture v manipulation | Pain (immediate term) | -0.08 [-0.49, 0.32] SMD | 0.682 | Fixed | 38.4 |
| 1, 100 |  | Pain (short term) | 0.01 [-0.38, 0.40] SMD | 0.958 | - | - |
| 2, 99 |  | Disability (immediate term) | 0.49 [0.08, 0.89] SMD | 0.019 | Fixed | 0 |
| 1, 120 | Acupuncture v traction | Pain (immediate term) | 1.31 [0.78, 1.84] MD | 0.000 | - | - |
| 1, 30 | Acupuncture v no treatment | Pain (immediate term) | 26 [3.686, 183.418] OR | 0.000 | - | - |
| **Acupuncture in LBP** |  |  |  |  |  |  |
| 9, 1387 | Acupuncture v sham acupuncture | Pain (immediate term) | -0.49 [-0.76, -0.21] SMD | 0.000 | Random | 72.8 |
| 8, 1368 |  | Pain (immediate term) # | -0.36 [-0.54, -0.19] SMD | 0.000 | Random | 39.7 |
| 6, 1261 |  | Pain (short term) | -0.45 [-0.76, -0.14] SMD | 0.004 | Random | 76.9 |
| 5, 1135 |  | Pain (short term) # | -0.31 [-0.56, -0.06] SMD | 0.014 | Random | 56.2 |
| 4, 1184 |  | Pain (intermediate term) | -0.17 [-0.28, -0.05] SMD | 0.005 | Fixed | 0 |
| 5, 1536 |  | Disability (immediate term) | -0.15 [-0.46, 0.16] SMD | 0.336 | Random | 83.0 |
| 4, 1517 |  | Disability (immediate term) # | 0.00 [-0.20, 0.20] SMD | 0.971 | Random | 66.0 |
| 3, 1436 |  | Disability (short term) | 0.07 [-0.10, 0.23] SMD | 0.420 | Random | 51.8 |
| 2, 1226 |  | Disability (short term) # | 0.14 [0.02, 0.25] SMD | 0.019 | Fixed | 0 |
| 4, 1525 |  | Disability (intermediate term) | -0.02 [-0.24, 0.20] SMD | 0.856 | Random | 71.3 |
| 3, 1315 |  | Disability (intermediate term) # | 0.07 [-0.12, 0.26] SMD | 0.460 | Random | 54.1 |
| 3, 188 | Acupuncture v sham acupuncture (acute LBP) | Pain (immediate term) | -0.99 [-1.24, -0.73] MD | 0.000 | Fixed | 0 |
| 4, 2911 | Acupuncture v no treatment | Pain (immediate term) | -0.73 [-0.96, -0.49] SMD | 0.000 | Random | 53.2 |
| 3, 2686 |  | Pain (immediate term) # | -0.57 [-0.65, -0.49] SMD | 0.000 | Fixed | 8.7 |
| 3, 451 |  | Disability (immediate term) | -0.95 [-1.42, -0.48] SMD | 0.000 | Random | 78.2 |
| 2, 267 |  | Disability (immediate term) # | -0.68 [-0.93, -0.42] SMD | 0.000 | Fixed | 7.9 |
| 2, 70 | Acupuncture v TENS | Pain (immediate term) | 0.46 [-3.16, 4.08] MD | 0.805 | Random | 73.4 |
| 2, 70 |  | Pain (short term) | -1.02 [-3.08, 1.04] MD | 0.333 | Fixed | 0 |
| 6, 242 | Acupuncture v medication | Pain (immediate term) | -0.52 [-1.27, 0.23] MD | 0.173 | Fixed | 42.9 |
| 4, 186 |  | Disability (immediate term) | -0.23 [-0.52, 0.06] SMD | 0.017 | Fixed | 28.7 |
| 6, 443 | Acupuncture v usual care | Pain (immediate term) | -1.56 [-2.45, -0.67] SMD | 0.001 | Random | 93.2 |
| 4, 195 |  | Pain (immediate term) # | -0.75 [-1.04, -0.46] SMD | 0.000 | Fixed | 0 |
| 5, 383 |  | Pain (follow-up) | -1.76 [-2.76, -0.75] SMD | 0.001 | Random | 93.1 |
| 3, 135 |  | Pain (follow-up) | -0.86 [-1.21, -0.50] SMD | 0.000 | Fixed | 29.7 |
| 5, 320 | Acupuncture plus usual care v usual care | Pain (immediate term) | -11.47 [-19.33, -3.61] MD | 0.004 | Random | 59.9 |
| 4, 269 |  | Pain (immediate term) # | -14.41 [-19.38, -9.45] MD | 0.000 | Fixed | 0 |
| 5, 320 |  | Pain (follow-up) | -14.30 [-26.07, -2.54] MD | 0.017 | Random | 82.1 |
| 4, 194 |  | Pain (follow-up) # | -8.50 [-14.50, -2.50] MD | 0.006 | Fixed | 0 |
| 4, 195 |  | Disability (immediate term) | -0.45 [-1.18, 0.29] SMD | 0.231 | Random | 81.9 |
| 3, 144 |  | Disability (immediate term) # | -0.75 [-1.32, -0.19] SMD | 0.009 | Random | 54 |
| 4, 195 |  | Disability (follow-up) | -0.55 [-1.00, -0.10] SMD | 0.016 | Random | 53.1 |
| **Acupressure in NP** |  |  |  |  |  |  |
| 1,32 | Acupressure + CT v conventional treatment (CT) | Pain (immediate term) | 23% reduction in VAS, effect size =0.43) | 0.02 | - | - |
|  |  | Pain (short term) | 23% reduction in VAS, effect size =0.43) | 0.02 | - | - |
| **Acupressure in LBP** |  |  |  |  |  |  |
| 2,275 | Acupressure v Physical therapy | Pain (immediate term) | -0.73 [-0.97, -0.48] SMD | 0.000 | Fixed | 0 |
|  |  | Pain (intermediate term) | -0.95 [-1.39, -0.51] SMD | 0.000 | Random | 67.2 |
| 1,129 | Acupressure v Physical therapy | Disability (immediate term) | -3.8 (-5.7, -1.9) MD | 0.000 | - | - |
|  |  | Disability (intermediate term) | -4.5 (-6.1, -2.9) MD | 0.000 | - | - |
| 2, 81 | Acupressure v Sham acupressure | Pain (immediate term) | -1.36 [-2.93, 0.21] SMD | 0.090 | Random | 84.8 |
|  |  | Pain (short term) | -0.36 [-0.98, 0.27] SMD | 0.266 | Fixed | 39.3 |
| 1,21 |  | Disability (immediate term) | -5.33 (-9.81, -0.85) MD | 0.000 | - | - |
|  |  | Disability (short term) | -4.23 (-7.83, -0.63) MD | 0.000 | - | - |
| 1,51 | Acupressure + CT v conventional treatment (CT) | Pain (immediate term) | -0.38 (-0.41, -0.35) MD | 0.000 | - | - |
|  |  | Disability (immediate term) | -0.12 (-0.14, -0.10) MD | 0.000 | - | - |
| **Cupping in NP** |  |  |  |  |  |  |
| 2, 93 | Cupping v waitlist | Pain (immediate term) | -19.10 [-27.61, -10.58]MD | 0.000 | Fixed | 0 |
|  |  | Disability (immediate term) | -6.65 [-10.97, -2.32] MD | 0.005 | Fixed | 1.2 |
| 1, 48 | Cupping v standard medical care | Pain (immediate term) | -1.72 [-2.74, -0.70] MD | 0.0009 | - | - |
|  |  | Disability (immediate term) | -5.78 [-10.80, -0.76] MD | 0.025 | - | - |
| 1, 40 | Cupping v heating pad | Pain (immediate term) | -36.30 [-46.48, -26.12]MD | 0.0009 | - | - |
|  |  | Pain (short term) | -21.55 [-34.92, -8.18] MD | 0.0009 | - | - |
|  |  | Disability (immediate term) | -7.69 [-13.68, -1.70] MD | 0.025 | - | - |
|  |  | Disability (short term) | -10.44 [-15.48, -5.40] MD | 0.025 | - | - |
| 1, 61 | Cupping v muscle relaxation | Pain (immediate term) | -0.16 [-13.90, 13.55] MD | 0.98 | - | - |
|  |  | Disability (immediate term) | -2.18 [-4.56, -0.21] MD | 0.07 | - | - |
| **Cupping in LBP** |  |  |  |  |  |  |
| 4, 430 | Cupping v medications | Pain (immediate term) | -0.54 [-0.89, -0.19] MD | 0.003 | Random | 84.5 |
| 3, 180 | Cupping (retention) v medications |  | -0.04 [-0.23, 0.15] MD | 0.686 | Random | 0 |
| 2, 120 | Cupping (balance) v medications |  | -0.65 [-0.81, -0.48] MD | 0.000 | Random | 0 |
| 1, 60 | Cupping (wet) v medications |  | -1.10 [-1.68, -0.52] MD | 0.000 | - | - |
| 1, 70 | Cupping (moving) v medications |  | -2.28 [-3.42, -1.14] MD | 0.000 | - | - |
| 3, 360 | Cupping v medications | Disability (immediate term) | -3.77 [-5.85, -1.69] MD | 0.000 | Random | 83.8 |
| 3, 180 | Cupping (retention) v medications |  | -1.41 [-2.67, -0.16] MD | 0.028 | Fixed | 0 |
| 2, 120 | Cupping (balance) v medications |  | -6.06 [-7.54, -4.57] MD | 0.000 | Fixed | 0 |
| 1, 60 | Cupping (wet) v medications |  | -5.90 [-7.57, -1.69] MD | 0.000 | - | - |
| 1, 32 | Cupping v waitlist | Pain (immediate term) | -6.9 [-19.16, 5.36] MD | 0.27 | - | - |
|  |  | Pain (2 weeks) | -0.8 [-12.16, 10.56] MD | 0.89 | - | - |
|  |  | Disability (immediate term) | -3.8 [-8.98, 1.38] MD | 0.15 | - | - |
|  |  | Disability (2 weeks) | -2.4 [-8.48, 3.68] MD | 0.44 | - | - |
| 1, 98 | Cupping v usual care | Pain (short term) | -2.20 [-2.60, -1.70] MD | 0.01 | - | - |
|  |  | Disability (short term) | -15.0 [-18.8, -11.2] MD | 0.01 | - | - |
| **Gua sha in NP** |  |  |  |  |  |  |
| 1,19 s | Gua sha v thermal therapy | Pain (immediate term) | -29.9 (-43.3, -16.6) MD | 0.000 | - | - |
|  |  | Disability (immediate term) | -8.5 (-13.6, -3.5) MD | 0.000 | - | - |
| 1,21 | Gua sha v wait list | Pain (immediate term) | -1.6 (-3.0, -0.1) MD | 0.000 | - | - |
| **Gua sha in LBP** |  |  |  |  |  |  |
| 1,19 | Gua sha v wait list | Pain (immediate term) | -1.1 (-2.0, -0.2) MD | 0.000 | - | - |
| **Qigong in NP** |  |  |  |  |  |  |
| 2, 161 | Qigong v waitlist | Pain (short term) | -15.27 [-22.49, -8.05] MD | 0.000 | Fixed | 47.5 |
|  |  | Pain (intermediate term) | -10.18 [-16.63, -3.73] MD | 0.002 | Fixed | 0 |
|  |  | Disability (short term) | -7.67 [-12.45, -2.88] MD | 0.002 | Fixed | 0 |
|  |  | Disability (intermediate term) | 0.43 [-4.43, 5.28] MD | 0.863 | Fixed | 0 |
| 2, 158 | Qigong v exercise | Pain (short term) | 1.88 [-5.78, 9.54] MD | 0.630 | Fixed | 0 |
|  |  | Pain (intermediate term) | 1.00 [-6.21, 8.21] MD | 0.785 | Fixed | 0 |
|  |  | Disability (short term) | 1.29 [-4.33, 6.91] MD | 0.652 | Fixed | 0 |
|  |  | Disability (intermediate term) | 0.02 [-5.25, 5.28] MD | 0.995 | Fixed | 13.3 |
| **Tai chi in LBP** |  |  |  |  |  |  |
| 1, 160 | Tai chi v Waitlist | Pain (immediate) | -1.3 (-0.7, -1.9) MD | 0.000 | - | - |
|  |  | Disability (immediate) | -5.7 (-1.8,- 9.6) MD | 0.000 | - | - |
| **Chinese herbal medicine in NP** |  |  |  |  |  |  |
| 1,360 | Exractum nucis vomicae v Diclofenac diethylamine emulgel | Pain (immediate) | -0.27 [-0.31, -0.23] MD | 0.000 | - | - |
| 1,240$ | Qishe vs. placebo | Pain (short term) | -0.67 [-0.82, -0.52] MD | 0.000 | - | - |
| 1,440$ | Qishe plus placebo Jingfukang v placebo Qishe plus Jingfukang | Pain (short term) | -0.08 [-0.18, 0.02] MD | 0.13 | - | - |
| **Chinese manipulation (CM) in NP** |  |  |  |  |  |  |
| 2, 183 | CM v Chinese massage | Pain (immediate term) | -2.00 [-2.55, -1.45] MD | 0.000 | Fixed | 0 |
| 1, 63 |  | Pain (short term) | -2.47 [-3.42, -1.52] MD | 0.000 | - | - |
| 1, 63 |  | Disability (immediate term) | -9.35 [-14.06, -4.64] MD | 0.000 | - | - |
|  |  | Disability (short term) | -10.81 [-15.78, -5.84] MD | 0.000 | - | - |
| 1, 213 | CM v cervical traction | Pain (immediate term) | -1.06 [-1.37, -0.75] MD | 0.000 | - | - |

*Intervention group minus control group.

§P value was for effect size.

#sensitivity analysis

$ study was unpublished.

CI, confidence interval; MD, mean difference; SMD, standard mean difference; RCT, randomized controlled trial; LBP, low back pain; NP, neck pain
