# Supplementary material for: Traditional Chinese Medicine for Neck Pain and Low Back Pain: A Systematic Review and Meta-Analysis
Source: PLoS One. 2015 Feb 24;10(2):e0117146. doi: 10.1371/journal.pone.0117146 (PMC4339195; doi:10.1371/journal.pone.0117146)
Supplement: S6 Table — (DOCX) [file pone.0117146.s007.docx]

**S6 Table.** The Basic Characteristics of Individual Studies Included.

| **Author Country** | **Study Characteristics** | **Population Characteristics** | **Pain Characteristics** | **Intervention Detail** | **Outcome Results: Pain, Disability** | | **Outcome Results: Other Outcomes; Harms** |
| --- | --- | --- | --- | --- | --- | --- | --- |
| **Acupuncture for NP** | | | | | | | |
| **Liang, 2011 [**[**21**](#_ENREF_21)**] China** | **Trial Design:** RCT  **Tx duration**: 3 wks  **Fu duration** (last assessment): 3mons  N screened: NR  N randomized:190  N completed tx: 183  N attended last fu: 178  **Inclusion**:(a)18 to 60 yrs; (b) neck pain or stiffness, >=1 monthly recurrence, >=6 mons; (c) (VAS,0-10) 3-7 points;  **Exclusion**:  (a) had received acupuncture due to neck pain in the past 6 months; (b) were unwilling to following the study; (c) had a history of cervical or thoracic (i.e., T1—T6) vertebra trauma, or had received surgery on the neck or had systematic neurological, skeletal disorders (i.e., positive ﬁnding in radiological examination); (d) afraid of acupuncture treatment; | **Mean age (SD/range)**:  IG= 36.72 (10. 21) yrs; CG= 37.25 (9.56) yrs  **% of female**: 72.5%  **Racial composition**: NR  **Other socio-demographics**:  education,  **Co morbidities**: NR  **Prior episode of pain if acute**: more than one monthly recurrence  **Prior CAM intervention**: no acupuncture for neck pain  **Prior surgery related to current complaint**: no surgery for neck pain | **Cause of Pain**:  Chronic neck  **Duration of Pain**:  IG= 50.43 (49.61)mons  CG=44.89 (36.78)mons  >= 6mons  **Severity of pain (Grading)**:  VAS (0-10)，  IG= 5.30 (1.91)  CG= 5.49 (1.56)  **Co-interventions**: NR | **Groups**  **IG** (n = 93) –Traditional acupuncture: 3 times per week during a total of 3 wks, nine sessions, 20 mins, inserted into the muscle (to a depth of 20mm), true acupoints.  **Drop outs**: A = 0, B =3,C=2  **CG** (n = 97) –Sham: sham points which were 1 cm lateral to the standard acupuncture points selected in the study group. Other the same as IG.  **Drop outs**: A = 0, B =4,C=3 | **Outcomes:**  **Pain**: VAS (0-10cm),  Not based on ITT  **Disability**: NPQ (0-100)%  Not based on ITT  **Results-Baseline:** mean (SD)  Pain**:** IG= 5.30 (1.91); CG= 5.49 (1.56)  Disability: IG = 32.73 (12.48), CG = 33.03 (10.64)  **Immediate post tx**:  Pain**:** IG = 3.48 (2.04), CG = 4.01 (1.45) Disability: IG = 20.71 (11.91), CG = 24.04 (11.83)  **1 month:**  Pain**:** IG = 2.89 (1.59), CG = 3.49 (1.41) Disability: IG = 17.44 (9.87), CG = 21.59 (12.23)  **Short term**:  Pain**:** IG = 2.88 (1.72), CG = 3.19 (1.31)Disability: IG = 19.09 (9.94), CG = 23.53 (13.67) | **Outcome**  **instruments**:  **QoL/ well being**: SF-36 (GH)  (0-100) **Other**: NR  **Results:**  **Baseline**:  IG = 49.14 (17.96), CG = 53.48 (15.93)  **immediate post tx**:  IG = 57.79 (16.74), CG = 56.58 (16.19)  **1 month:**  IG = 60.71 (17.23), CG = 57.87 (16.92)  **Short term**:  IG = 60.76 (16.51), CG = 59.90 (17.40)  **intermediate**: NR  **Long term**: NR  **Harms**: local bleeding on the selected points, local numbness and aching, and fainting during acupuncture. seven participants (three in the study group and four in the control group) fainted. Four participants in the study group and two in the control group complained of feeling numb and aching on the treated points | |
| **Sahin, 2010 [**[**22**](#_ENREF_22)**] Turkey** | **Trial Design:** RCT  **Tx duration**: 3 wks  **Fu duration** (last assessment): 3mons  N screened: 40  N randomized:31  N completed tx: 29  N attended last fu: 29  **Inclusion**:(a)18 to 65 yrs; (b) chronic soft tissue neck pain, >=3 mons; (c) (VAS,0-10) >=3 points; not respond to physical therapy, medical therapy or collar continued for one month; not previously received acupuncture therapy.  **Exclusion**: having complaints of radicular pain, neurological deficits, and disk hemiation; lumbar pain for the last three months with a VAS score above five; radiological evidence of narrowing of cervical neural foramen and facet osteoarthritis; fracture; congenital neck deformities such as lordosis and seoliosis except mild eases; spondylolysis or spondylolisthesis; history of trauma, an so on. | **Mean age (SD/range)**:  IG= 38.50 ± 10.47 yrs; CG= 35.20 ±9.18 yrs  **% of female**: 89.3%  **Racial composition**: NR  **Other socio-demographics**:  education, marriage  **Co morbidities**: NR  **Prior episode of pain if acute**:  **Prior CAM intervention**: no acupuncture for neck pain  **Prior surgery related to current complaint**: no surgery for neck pain | **Cause of Pain**:  Chronic soft tissue,  **Duration of Pain**:  >= 3mons  **Severity of pain (Grading)**:  VAS (0-10)，  IG= 4.00±3.03; CG= 5.25±1.95  **Co-interventions**: NR | **Groups**  **IG** (n = 15) –Electro-acupuncture: 3 times per week d for a total of 10 sessions, 30 mins, steel acupuncture needles, electro stimulated until Diqi perception, to a depth of 2 cm.  **Drop outs**: A = 0, B =2,C=0  **CG** (n = 16) –Sham acupuncture: sham points which were 1-2 cm lateral to the standard acupuncture points selected in the study group. Other the same as IG.  **Drop outs**: A = 0, B =0,C=0 | **Outcomes:**  **Pain**: VAS (0-10cm),  Not based on ITT  **Disability**: NR  Not based on ITT  **Results-Baseline:** mean (SD)  Pain**:** IG= 4.00±3.03; CG= 5.25±1.95  Disability: NR  **Immediate post tx**:  Pain**:** IG = 5.06±1.8l, CG = 3.46±2.93  Disability: NR  **1 month:**  NR  **Short term**:  Pain**:** IG = 4.00±2.97, CG = 3.54±3.13  Disability: NR  **Intermediate**: NR | **Outcome**  **instruments**:  **QoL/ well being**: SF-36 (Bodily pain)  (0-100)  **Other**: NR  **Results:**  **Baseline**: Unclear  **immediate post tx**: Unclear  **1 month:** NR  **Short term**: Unclear  **intermediate**: NR  **Long term**: NR  **Harms**: NR | |
| **Fu, 2009 [**[**23**](#_ENREF_23)**] China** | **Trial Design:** RCT  **Tx duration**: 18 days  **Fu duration** (last assessment): 3mons  N screened: NR  N randomized:117  N completed tx: 112  N attended last fu: 112  **Inclusion**: The diagnosis of cervical spondylosis CS was made according to the "Standard for diagnosis and efficacy evaluation of traditional Chinese medicine syndromes and diseases" issued by the State Administration of Traditional Chinese Medicine (1994) (a)18 to 60 yrs; (b) having not received acupuncture treatment on neck pain in the latest 6 months;  **Exclusion**:  (1) patients with CS other than cervical type or nerve root type; (2) having a history of neck trauma; (3) women in the pregnancy or lactation period; and (4) complicated with severe internal disease or tumor. | **Mean age (SD/range)**:  IG= 34.33±10.32 yrs; CG= 35.45±10.27 yrs  **% of female**: 72.6%  **Racial composition**: NR  **Other socio-demographics**: NR  **Co morbidities**: NR  **Prior episode of pain if acute**: NR  **Prior CAM intervention**: no acupuncture for neck pain at near 6 month  **Prior surgery related to current complaint**: NR | **Cause of Pain**:  cervical spondylosis  **Duration of Pain**:  IG=46.93±45.25  (6-248) mons,  CG=37.82±34.04(6-156)mons  **Severity of pain (Grading)**:  VAS (0-10)，  IG= 5.14±1.83  CG= 5.58±1.64  **Co-interventions**: infrared radiation in both group | **Groups**  **IG** (n = 59–Normal acupuncture: once every other day, 9 sessions (in 18 days) as one therapeutic course, 20 mins, inserted into the muscle (to a depth of 20mm), true acupoints. operated to get the due sensation (called Diqi)  "Deqi"  **Drop outs**: A = 0, B =2,C=0  **CG** (n = 58–Sham acupuncture: sham points which were 1 cm lateral to the standard acupuncture points selected in the study group. Other the same as IG.  **Drop outs**: A = 0, B =3,C=0 | **Outcomes:**  **Pain**: VAS (0-10cm),  Not based on ITT  **Disability**: NPQ (0-100)%  Not based on ITT  **Results-Baseline:** mean (SD)  Pain**:** IG= 5.14±1.83; CG= 5.58±1.64  Disability: IG = 33.63±12.68, CG = 33.21±11.34  **Immediate post tx**:  Pain**:** IG = 3.19±1.92, CG = 4.14±1.59  Disability: IG = 19.98±11.30, CG = 25.02±13.03  **1 month:**  Pain**:** IG = 2.66±1.47, CG = 3.58±1.62  Disability: IG = 17.61±10.04, CG = 23.70±13.94  **Short term**:  Pain**:** IG = 2.89±1.81, CG = 3.28±1.48  Disability: IG = 20.55±10.20, CG = 25.77±15.05 | **Outcome**  **instruments**:  **QoL/ well being**: NR  **Other**: NR  **Results:**  **Baseline**: NR  **immediate post tx**: NR  **1 month:**NR  **Short term**: NR  **intermediate**: NR  **Long term**: NR  **Harms**: NR | |
| **Itoh, 2007 [**[**24**](#_ENREF_24)**]Japan** | **Trial Design:** RCT  **Tx duration**: 3 wks  **Fu duration** (last assessment): 3mons  N screened: NR  N randomized:36  N completed tx: 31  N attended last fu: 31  **Inclusion**:Pts with CNP (> 6mo) age>=45 yrs, no radiation of NP, well functioning cervical neve, deep tendon reflexes, voluntary muscle action, sensory and function  **Exclusion**: Major trauma or systemic disease, other ongoing tx except those receiving unified dosage for a mo or longer | **Mean age (SD/range)**:  IG1= 62.3(11) yrs;IG2= 62.3(10.1) yrs  **% of female**: 72.5%  **Racial composition**: Asian  **Other socio-demographics**: **NR**  **Co morbidities**: NR  **Prior episode of pain if acute**: NR  **Prior CAM intervention**: NR  **Prior surgery related to current complaint**:NR | **Cause of Pain**:  N-S NP  **Duration of Pain**:  IG= 3.2(3.1)  CG=2.9 (2.7)yrs  **Severity of pain (Grading)**:  NR  **Co-interventions**: NR | **Groups**  **IG1** (n = 8) –Traditional acupuncture: needles inserted into muscle to depth of 20min-“sparrow pecking” technique-needle retention for 10 min-or until “deqi” sensation; 3wks  **Drop outs**: A = 2  IG (n=8)-TP-Acu: applied to myofascial TPs located by palpation, local twitch elicided-similar technique as IG1; 3wks  **Drop outs**: A = 2  **CG1**(n = 10)-Non-TP-Acu: NR; NR  **CG2** (n = 10) –Sham: NR; NR | **Outcomes:**  **Pain**: VAS (0-100mm),  based on ITT  **Disability**: NDI  Not based on ITT  **Results-Baseline:** mean (SD)  Pain**:** IG1= 69.5(18.6); IG2=67(13.2), CG1= 70.9(14), CG2=64.1(20.7)  Disability: IG1= 12.6(6); IG2=13(6.3), CG1= 15.1(2.7), CG2=12(3.6)  **Imme** IG1= 45.9(17.5); IG2=18.6(18.5), CG1= 58.4(16.9), CG2=54.6(20) Disability: IG1= 9.3(5.2); IG2=3.9(3.4), CG1= 12.8(2.1), CG2=11.3(3.3)  **Short term:**  Disability (combined): IG=10.9(6.6), CG=11.1(5) | **Outcome**  **instruments**:  **QoL/ well being**: NR  **Other**: NR  **Results:**  **Baseline**: NR  **immediate post tx**: NR  **1 month:**NR  **Short term**: NR  **intermediate**: NR  **Long term**: NR  **Harms**: NR | |
| **Nabeta, 2002 [**[**28**](#_ENREF_28)**]Japan** | **Trial Design:** RCT  **Tx duration**: 3 wks  **Fu duration** (last assessment): 1mon  N screened: NR  N randomized:34  N completed tx: 27  N attended last fu: NR  **Inclusion**:Pts with chronic pain/stiffness in neck and shoulder without arm symptoms  **Exclusion**:  NR | **Mean age (SD/range)**:  IG= 34.2 (10.8) yrs; CG= 30.8 (12) yrs  **% of female**: 70.6%  **Racial composition**: Asian  **Other socio-demographics**:  NR  **Co morbidities**: NR  **Prior episode of pain if acute**: Myofascial syndome  **Prior CAM intervention**: NR  **Prior surgery related to current complaint**:NR | **Cause of Pain**:  N-S chronic neck, shoulder pain  **Duration of Pain**:  NR  **Severity of pain (Grading)**:  NR  **Co-interventions**: NR | **Groups**  **IG** (n = 17) –Traditional acupuncture: needles inserted into the muacle to a depth of 20mm and the “sparrow pecking” technique was applied; when dull pain or acu sensation was felt, the manipulation was stransverse oscillatory rotped and the needle was retained for 5 more min; 3tx, 3wks  **Drop outs**: 2 (A-B)  **CG** (n = 17) –Sham: similar needles used but tips had been cut off and smoothed to prevent penetration of skin  **Drop outs**: 5(A-B) | **Outcomes:**  **Pain**: VAS (100mm),  based on ITT  **Disability**: NR  **Results-Baseline:** mean (SD)  Pain**:** IG= 60.5(15); CG= 48.8 (28)  **Immediate post tx**:  Pain**:** IG = 43.3 (19.7), CG = 46.8 (25.4)  **1 month:**  NR  **Short term**:  NR | **Outcome**  **instruments**:  **QoL/ well being**: NR  **Other**: NR  **Results:**  **Baseline**: NR  **immediate post tx**: NR  **1 month:**NR  **Short term**: NR  **intermediate**: NR  **Long term**: NR  **Harms**: author indicate that AEs were not the cause of drop out | |
| **Zhu, 2002 [**[**27**](#_ENREF_27)**] Australia** | **Trial Design:** RCT crossover  **Tx duration**: 3 wks  **Fu duration** (last assessment): immediate post-tx  N screened: NR  N randomized:29  N completed tx: 29  N attended last fu: 29  **Inclusion**:Pts with CNP, 31-71 yrs had neck complaints >=6mo, degenerative joint disease, osteoarthritis, cervical spondylitis, soft tissue injuries, cervical sprain or whiplash injury, pain felt in the neck and radiating to the occiput or shoulders limiting neck movement  **Exclusion**: cancer, decreased or sbsent deep tendon reflexes, depression, fibromyalgia syndrome, pregnancy, previous cervical spine surgery, acu tx, hypertension, thyroid problem, or diabetes | **Mean age (SD/range)**:  IG= 50(10.6) yrs; CG= 48.9 (10.1) yrs  **% of female**: IG=36%, CG=60%  **Racial composition**: Asian  **Other socio-demographics**:  NR  **Co morbidities**: NR  **Prior episode of pain if acute**: neck injury, n=17  **Prior CAM intervention**: NR  **Prior surgery related to current complaint**:NR | **Cause of Pain**:  N-S NP  **Duration of Pain**:  IG=79.8(60)mo; CG=59.7(104.9)mo  **Severity of pain (Grading)**:  NR  **Co-interventions**: NR | **Groups**  **IG** (n = 14) –Acu: Chinese acu dry needling both on two local and 2 distal points; 9sessions; 3wks  **Drop outs**: NR  **CG** (n = 15) –Sham: sham acu points located 2-3cm lateral to the real points; short needles used; weak electro-stimulation once/min was applied for two distal points; other the same as IG  **Drop outs**: NR | **Outcomes:**  **Pain**: VAS (10cm),  based on ITT  **Disability**: NDI  **Results-Baseline:** mean (SD)  Pain**:** IG = 5.18±1.92, CG = 4.03±1.65  Disability: IG = 10.2±4.7, CG = 8.2±3.6  **Immediate post tx**:  Pain**:** IG = 2.89±2.8, CG = 2.11±2.2  Disability: IG = 6±4.5, CG = 5.7±3.1  **1 month:**  NR  **Short term**:  NR | **Outcome**  **instruments**:  **QoL/ well being**: NR  **Other**: NR  **Results:**  **Baseline**: NR  **immediate post tx**: NR  **1 month:**NR  **Short term**: NR  **intermediate**: NR  **Long term**: NR  **Harms**: NR | |
| **Birch, 1998[**[**30**](#_ENREF_30)**] USA** | **Trial Design:** RCT  **Tx duration**: 12 wks  **Fu duration** (last assessment): 6mo  N screened: 59  N randomized:46  N completed tx: 46  N attended last fu: 36  **Inclusion**:Chronic myofascial NP (>6 mo), identifiable painful area with heightened sensitivity to moderate touch; unsuccessful response to physical therapy (traction, heat, US, massage)  **Exclusion**: disc herniation, cervical osteoarthritis, infection, malignancy, collapsed vertebra, collagen-vascular disease, brachial plexopathy, schizophrenia, delusional psychotic, or bipolar disorder | **Mean age (SD/range)**:  IG1= 40.9 ;IG2= 38, CG= 38.6 yrs  **% of female**: IG1=75.7%, IG2=77%, CG=75.7%  **Racial composition**: NR  **Other socio-demographics**: 33.6% married  **Co morbidities**: NR  **Prior episode of pain if acute**: NR  **Prior CAM intervention**: NR  **Prior surgery related to current complaint**:NR | **Cause of Pain**:  S, NP  **Duration of Pain**:  IG1= 81.9, IG2= 92.2;CG=91.1mo  **Severity of pain (Grading)**:  NR  **Co-interventions**: 500 mg/d NSAIDs | **Groups**  **IG1** (n = 15) –acupuncture: needles inserted into points 9SI3, BL62, GB41, TW5 with a depth of 2-3 mm, needles connected to IP cords and left in place for 10min or until “deqi” sensation; 14 tx, 12wks  **Drop outs**: D= 4  IG (n=16)-Sham Acu: shallow needling with gauge 2 (0.18 mm) bilaterally to 2-3 mm depth in hands and feet at LI5, GB42, TW8, ST41, needles connected by cords as like IG1, left for 10 min, points BL16, SI9, LI15 needled bilaterally by 6 needles to 2-3 mm depth; same as IG1  **Drop outs**: D = 2  **CG1**(n = 15)-Medication: NSAID; 12 wks  **Drop outs**: D = 3 | **Outcomes:**  **Pain**: VAS (0-10 cm), based on ITT  **Disability**: NR  **Results-Baseline:** mean (SD)  Pain**:** IG1= 4.8; IG2=4.7, CG= 4.9  **Immediate post tx**: IG1= 1.87(1.9); IG2=3.37(2.14), CG= 4.76(2.05)  **Short term: NR** | **Outcome**  **instruments**:  **QoL/ well being**: NR  **Other**: NR  **Results:**  **Baseline**: NR  **immediate post tx**: NR  **1 month:**NR  **Short term**: NR  **intermediate**: NR  **Long term**: NR  **Harms**: NR | |
| **Vas, 2006 [**[**25**](#_ENREF_25)**] Spain** | **Trial Design:** RCT  **Tx duration**: 3 wks  **Fu duration** (last assessment): 6 mo  N screened: 149  N randomized:123  N completed tx: 123  N attended last fu: 85  **Inclusion**: >= 17yrs with uncomplicated NP (>3mo), symptomatic at examination, motion-related NP (>=3 on VAS10), no tx during past 1 wk  **Exclusion**: previous acu tx, neuropathology, infections, inflammation, tumor, endocrine, metabolic, fracture, trauma, severe psychiatric illness, severe disorder of overall health state, pregnancy | **Mean age (SD/range)**:  IG= 46(13.7) yrs; CG= 47.4 (12.8) yrs  **% of female**: IG=75.4%, CG=88.7%  **Racial composition**: NR  **Other socio-demographics**: 28.4% sedentary  **Co morbidities**: NR  **Prior episode of pain if acute**: NR  **Prior CAM intervention**: NR  **Prior surgery related to current complaint**:NR | **Cause of Pain**:  S (86.15%), NP  **Duration of Pain**:  IG=47.4(60.3)mo; CG=43(40.8)mo  **Severity of pain (Grading)**: >=3 VAS  **Co-interventions**: 50mg diclophenac; 50mg tetrazepam | **Groups**  **IG** (n = 61) –Acu: bilateral points with needle manually stimulated every 10 min; “deqi” sensation, needle retention 30 min, vaccaria seeds taped in ear auricle after sterilizing skin after removing needles; pts instructed to apply pressure to each ear points 10 repeats 3 times/d; 5 sessions; 3wks  **Drop outs**: B=3, C=13  **CG** (n = 62) –Placebo TENS: electrodes at GB21 bilateral points with pt in prone position; nerve stimulation unit in front of pt for 30 min with visible and audible flashing diode  **Drop outs**: B =5, C= 17 | **Outcomes:**  **Pain**: VAS (10cm),  based on ITT  **Disability**: NPQ (100)  **Results:** mean (SD)  **Immediate post tx**:  Pain**:** IG = 4.21±2.11, CG = 1.4±1.57  Disability: IG = 30.2±13.6, CG = 12.7±14.9  **Short term**:  Pain**:** IG = 4.11±2.69, CG = 2.68±2.59 | **Outcome**  **instruments**:  **QoL/ well being**: SF-36  **Other**: NR  **Results:**  **Baseline**: NR  **immediate post tx**: NR  **1 month:**NR  **Short term**: NR  **intermediate**: NR  **Long term**: NR  **Harms**: mild AEs similar rated in IG and CG (Acu: 4 AEs swelling of hands, bruising, pain and ulcer of the ear vs. placebo 2 Aes cepalea, and aggrevation of symptoms) | |
| **White, 2004[**[**26**](#_ENREF_26)**] UK** | **Trial Design:** RCT  **Tx duration**: 4 wks  **Fu duration** (last assessment): 12 mo  N screened: 202  N randomized:135  N completed tx: 135  N attended last fu: 106  **Inclusion**: aged 18-80yrs with chronic mechanical NP (>2mo), NP (>=3 on VAS10), no tx during past 5-7 d  **Exclusion**: pregnancy, history of fracture, surgery of the neck, cervical congenital abnormality, uncontrolled LBP, contraindication to acetaminophen, systemic illness, recent or current manual neck tx or steroid use | **Mean age (SD/range)**:  IG= 53.9(15.71) yrs; CG= 52.8 (15.6) yrs  **% of female**: IG=65.8%, CG=63.1%  **Racial composition**: NR  **Other socio-demographics**: NR  **Co morbidities**: NR  **Prior episode of pain if acute**: NR  **Prior CAM intervention**: NR  **Prior surgery related to current complaint**:None | **Cause of Pain**:  N-S, NP  **Duration of Pain**:  IG=4.81(7.03); CG=7.71(11.4)yrs  **Severity of pain (Grading)**: >=3 VAS 10  **Co-interventions**: acetaminophen | **Groups**  **IG** (n = 70) –Acu: needles without guide tubes, points selection based on individualized western theory; points determined by pain distribution, palpation of the neck and thorax to find ah-shi points/ local tender points. At least one distal point was used; 6 points on avg/side, 20 min, 8 sessions; 4wks  **Drop outs**: D=16  **CG** (n = 65) –Placebo TENS: the cables were severed at the output plug and no current was delivered to the pt; examination and point selection were the same as in IG; same as IG  **Drop outs**: D=12 | **Outcomes:**  **Pain**: VAS (10cm),  based on ITT  **Disability**: NDI (100)  **Results:** mean (SD)  **Immediate post tx**:  Pain**:** IG = 2.04±2.03, CG = 3.07±2.2  Disability: IG = 11.78±6.59, CG = 12.34±7.35  **Short term**:  Pain**:** IG = 1.73±1.9, CG = 2.32±2.09  Disability: IG = 10.98±6.27, CG = 12.68±7.79 | **Outcome**  **instruments**:  **QoL/ well being**: SF-36  **Other**: NR  **Results:**  **Baseline**: NR  **immediate post tx**: NR  **1 month:**NR  **Short term**: NR  **intermediate**: NR  **Long term**: NR  **Harms**: increase in symptoms after tx (n=1), faintness (n=3), mild headache (n=2), dizziness (n=2), tiredness (n=1), thumb tingling (n=1), cold feeling (n=1), nausea (n=1), discomfort (n=1), hand swelling (n=1), bruise at LI4 (n=1), euphoria and enhanced vision (n=1) | |
| **Petrie, 1986[**[**31**](#_ENREF_31)**] UK** | **Trial Design:** RCT  **Tx duration**: 4 wks  **Fu duration** (last assessment): 3 mo  N screened: 27  N randomized:25  N completed tx: 24  N attended last fu: 24  **Inclusion**: CNP (>6mo)  **Exclusion**: peripheral synovitis or malignancy | **Mean age (SD/range)**:  IG= 52.9(9.8) yrs; CG= 48.1 (12.8) yrs  **% of female**: IG=69%, CG=58%  **Racial composition**: NR  **Other socio-demographics**: NR  **Co morbidities**: NR  **Prior episode of pain if acute**: NR  **Prior CAM intervention**: NR  **Prior surgery related to current complaint**:NR | **Cause of Pain**:  N-S, NP  **Duration of Pain**:  IG=18(11.2); CG=26.5(26.4)mo  **Severity of pain (Grading)**: NR  **Co-interventions**: NR | **Groups**  **IG** (n = 13) –Acu: Five standard needles inserted at points GB10 and GB21 bilaterally and DU14 in the mid-line. “deqi” by ME on insertion and at 5 min intervals for 20 min, 8 sessions; 4wks  **Drop outs**: B=0, C=0  **CG** (n = 12) –Placebo TENS: the cables were severed at the output plug and no current was delivered to the pt; examination and point selection were the same as in IG; same as IG  **Drop outs**: B=1, C=0 | **Outcomes:**  **Pain**: VAS (10cm),  based on ITT  **Disability**: VAS (100)  **Results:** mean (SD)  **Immediate post tx**:  Pain**:** IG = 3.66±2.3, CG = 3.29±1.86  Disability: IG = 25.98±23.67, CG = 25.85±20.27  **Short term**:  Pain**:** IG = 3.18±2.41, CG = 2.47±2.06  Disability: IG = 24.74±25.44, CG = 22.67±23.85 | **Outcome**  **instruments**:  **QoL/ well being**: NR  **Other**: NR  **Results:**  **Baseline**: NR  **immediate post tx**: NR  **1 month:**NR  **Short term**: NR  **intermediate**: NR  **Long term**: NR  **Harms**: one pt in placebo experiences negative effects | |
| **Irnich, 2001[**[**29**](#_ENREF_29)**] Germany** | **Trial Design:** RCT  **Tx duration**: 3 wks  **Fu duration** (last assessment): 6mo  N screened: 182  N randomized:177  N completed tx: 177  N attended last fu: 165  **Inclusion**: NP (>1 mo), painful restriction of cervical spine mobility who had not received any tx 2wks before  **Exclusion**: dislocation, had surgery, fracture, neurological deficits, systemic disorders, tx contraindications | **Mean age (SD/range)**:  IG1= 52.3(13.3) ;IG2= 52.7(11.5), CG= 52.2(13.2) yrs  **% of female**: 66.1% total  **Racial composition**: NR  **Other socio-demographics**: NR  **Co morbidities**: NR  **Prior episode of pain if acute**: (n) whiplash =56, myofascial pain= 129  **Prior CAM intervention**: NR  **Prior surgery related to current complaint**: None | **Cause of Pain**:  S (whiplash, myofascial pain), NP  **Duration of Pain**:  NR  **Severity of pain (Grading)**:  NR  **Co-interventions**: None | **Groups**  **IG1** (n = 56) –acupuncture: traditional Chinese medicine theory, local MTPs treated with dry needling to elicit local twitch, common points S13, UB10, UB60, Liv3, GB20, GB34, TE5, and the ear point cervical spine; 5 tx, 3 wks  **Drop outs**: D= 7  **IG2** (n=60)-Massage: Conventional western massage; same as IG1  **Drop outs**: D = 1  **CG** (n = 61)-Sham laser: inactivated laser pen, every point treated for 2min at 0.5-1cm distance from the skin; same as IG1  **Drop outs**: D = 4 | **Outcomes:**  **Pain**: VAS (0-10 cm), based on ITT  **Disability**: NR  **Results:** mean (SD)  **Immediate post tx**: IG1= -2.42(2.8); IG2=-0.79(2.75), CG= -1.73(2.81)  **Short term: NR** | **Outcome**  **instruments**:  **QoL/ well being**: SF-36  **Other**: NR  **Results:**  **Baseline**: NR  **immediate post tx**: NR  **1 month:**NR  **Short term**: NR  **intermediate**: NR  **Long term**: NR  **Harms**: mild reactions n=17 (33%) in IG1, 4 (7%) in IG2, and 12 (21%) in CG | |
| **Zhang, 2003[**[**36**](#_ENREF_36)**]China** | **Trial Design:** RCT  **Tx duration**: 45d  **Fu duration** (last assessment): NR  N screened: NR  N randomized:120  N completed tx: 120  N attended last fu: NR  **Inclusion**: cervical spondylosis  **Exclusion**: acute external injury cause, not compliant | **Mean age (SD/range)**:  NR  **% of female**: IG=66.7%, CG=45%  **Racial composition**: Asian  **Other socio-demographics**: NR  **Co morbidities**: NR  **Prior episode of pain if acute**: NR  **Prior CAM intervention**: NR  **Prior surgery related to current complaint**:NR | **Cause of Pain**: Spondylosis, NP  **Duration of Pain**: NR  **Severity of pain (Grading)**: NR  **Co-interventions**: NR | **Groups**  **IG** (n = 60) –Electro-Acu: tianzhu, jinbailao and dashu (bilaterally) for major acu points, frequency 120-250/min, 1 tx/d, 15tx/course, 3 courses, 2d interval between courses  **Drop outs**: B=0  **CG** (n = 60) –Traction:30 min, average traction weight =7.5kg; same as IG  **Drop outs**: B=0 | **Outcomes:**  **Pain**: VAS (10cm),  based on ITT  **Disability**: NR  **Results:** mean (SD)  **Immediate post tx**:  Pain**:** IG = 3.66±2.3, CG = 3.29±1.86  Disability: IG = 25.98±23.67, CG = 25.85±20.27  **Short term**:  NR | **Outcome**  **instruments**:  **QoL/ well being**: NR  **Other**: NR  **Results:**  **Baseline**: NR  **immediate post tx**: NR  **1 month:**NR  **Short term**: NR  **intermediate**: NR  **Long term**: NR  **Harms**: IG in therapeutic effect and improvement of pain for cervical spondylosis is better than the CG. | |
| **Thomas, 1991[**[**34**](#_ENREF_34)**] Sweden** | **Trial Design:** RCT  **Tx duration**: unclear  **Fu duration** (last assessment): immediate post-tx  N screened: NR  N randomized:44  N completed tx: NR  N attended last fu: NR  **Inclusion**: chronic cervical osteoarthritis, pain (>=6mo), pain more severe when joints are in movement than at rest  **Exclusion**: NR | **Mean age (SD/range)**:  42-77 yrs  **% of female**: NR  **Racial composition**: NR  **Other socio-demographics**: NR  **Co morbidities**: NR  **Prior episode of pain if acute**: NR  **Prior CAM intervention**: NR  **Prior surgery related to current complaint**: NR | **Cause of Pain**:  Cervical osteoarthritis, NP  **Duration of Pain**:  NR  **Severity of pain (Grading)**:  NR  **Co-interventions**: None | **Groups**  **IG1** (n = 11) –acupuncture: needles insertion with a depth 0.6-1.3 cm. Stimulation manually with ‘deqi’ for 40 min, repeated 10second/5min by further rots, 3-5d between trials  **Drop outs**: NR  CG1 (n=11)-Sham-Acu: Needles inserted superficially and left without eliciting further sensation; 3-5 d between trials  **Drop outs**: NR  **IG2** (n = 11)-**medication**: 5mg diazepam orally; same as IG1  **Drop outs**: NR | **Outcomes:**  **Pain**: VAS (0-10 cm), based on ITT  **Disability**: NR  **Results:** mean (SD)  Baseline: IG1=3.5(1.2), CG1=3.1(1.1), IG2=3.0(0.8)  **Immediate post tx**: IG1=2.3(1.5); CG1=-2.4(1.2), CG2= 2.2(1)  **Short term: NR** | **Outcome**  **instruments**:  **QoL/ well being**: SF-36  **Other**: NR  **Results:**  **Baseline**: NR  **immediate post tx**: NR  **1 month:** NR  **Short term**: NR  **intermediate**: NR  **Long term**: NR  **Harms**: NR | |
| **Giles, 2003[**[**32**](#_ENREF_32)**] Australia** | **Trial Design:** RCT  **Tx duration**: 9 wks  **Fu duration** (last assessment): 12 mo  N screened: 109  N randomized:109  N completed tx: 109  N attended last fu: 62  **Inclusion**: age >=17yrs with uncomplicated mechanical spinal pain for minimum of 13 wks  **Exclusion**: nerve root involvement, spinal anomalies, pathology other than mild-moderate osteoarthritis, leg length inequality >9mm with postural scoliosis | **Mean age (SD/range)**:  IG1= 23.8(4.8) ;IG2= 25(8.1), CG= 29.5(2.07) yrs  **% of female**: 45% total  **Racial composition**: NR  **Other socio-demographics**: NR  **Co morbidities**: NR  **Prior episode of pain if acute**: NR  **Prior CAM intervention**: NR  **Prior surgery related to current complaint**: None | **Cause of Pain**:  N-S, NP, LBP, thorax  **Duration of Pain**:  Chronic (>13 wks)  **Severity of pain (Grading)**:  NR  **Co-interventions**: None | **Groups**  **IG1** (n = 34) –Acu: near and far techniques as chosen by clinician; 2 tx/wk, max. of 9 wks  **Drop outs**: B= 12  **IG2** (n=35)-Spinal manipulation: 20 min appointments. High-velocity, low-amplitude thrust SM to a joint; same as IG1  **Drop outs**: B=10  **CG** (n = 40)-Medication: celecoxib/celebrx (200-400 mg/d); rofecoxib/vioxx (12.5-25 mg/d); NR  **Drop outs**: B=18 | **Outcomes:**  **Pain**: VAS (0-100 mm), based on ITT  **Disability**: ODI  **Results:** mean (SD)  Baseline:  Pain: IG1= 6(2.2); IG2= 6(2.9), CG= 5(3.7)  Dsiability: IG1= 30(17.03); IG2=-22(22.96), CG= 32(19.3)  **Immediate post tx**: Pain: IG1= 4(4.4); IG2= 5(3.7), CG= 6(4.4)  Dsiability: IG1= 26(20.74); IG2= 14(24.4), CG= 32(23.7)  **Short term: NR** | **Outcome**  **instruments**:  **QoL/ well being**: SF-36  **Other**: NR  **Results:**  **Baseline**: NR  **immediate post tx**: NR  **1 month:**NR  **Short term**: NR  **intermediate**: NR  **Long term**: NR  **Harms**: NR | |
| **Giles, 1999[**[**33**](#_ENREF_33)**] Australia** | **Trial Design:** RCT  **Tx duration**: 3-4 wks  **Fu duration** (last assessment): immediate post-tx  N screened: 875  N randomized:40  N completed tx: 40  N attended last fu: 40  **Inclusion**: age >=18yrs with uncomplicated mechanical spinal pain for minimum of 13 wks  **Exclusion**: nerve root involvement, spinal anomalies, pathology other than mild-moderate osteoarthritis, leg length inequality >9mm with postural scoliosis | **Mean age (SD/range)**:  IG1= 46.5(9.6) ;IG2= 42.5(9.6), CG= 35(14.1) yrs  **% of female**: 64.3% total  **Racial composition**: NR  **Other socio-demographics**: NR  **Co morbidities**: NR  **Prior episode of pain if acute**: NR  **Prior CAM intervention**: NR  **Prior surgery related to current complaint**: None | **Cause of Pain**:  N-S, NP  **Duration of Pain**:  Chronic (>13 wks)  **Severity of pain (Grading)**:  NR  **Co-interventions**: None | **Groups**  **IG1** (n = 34) –Acu: near and far techniques as chosen by clinician; 2 tx/wk, 3-4 wks  **Drop outs**: NR  **IG2** (n=35)-Spinal manipulation: 20 min appointments. High-velocity, low-amplitude thrust SM to a joint; same as IG1  **Drop outs**: NR  **CG** (n = 40)-Medication: tenoxican (20 mg/d) and ranitidine (50 mg twice/d); 15-20 min/appointment, 3-4 wks  **Drop outs**: NR | **Outcomes:**  **Pain**: VAS (0-100 mm), based on ITT  **Disability**: ODI  **Results:** mean (SD)  Baseline:  Pain: IG1= 40(31.8); IG2= 32(14.8), CG= 28(21.9)  Dsiability: IG1= 3.5(5.5); IG2=-5(3.5), CG= 2.7(4.8)  **Immediate post tx**: Pain mean change: IG1= -6(14.4); IG2= -10(10.4), CG= 0(10.7)  Dsiability: IG1= -0.5(4.8); IG2= -2.3(4.8), CG= -1(1.3)  **Short term: NR** | **Outcome**  **instruments**:  **QoL/ well being**: NR  **Other**: NR  **Results:**  **Baseline**: NR  **immediate post tx**: NR  **1 month:**NR  **Short term**: NR  **intermediate**: NR  **Long term**: NR  **Harms**: NR | |
| **Li, 2006[**[**35**](#_ENREF_35)**] China** | **Trial Design:** RCT  **Tx duration**: 2-4 wks  **Fu duration** (last assessment): 6 mo  N screened: 150  N randomized:150  N completed tx: 150  N attended last fu: 150  **Inclusion**: spinal stenosis of neck; age <69 yrs; disease course< 2yrs; diagnosed by CT or MRI; related signs are positive  **Exclusion**: spinal trauma in 4 mo; systemic infection and fever; cervical tumor | **Mean age (SD/range)**:  49 yrs total  **% of female**: 46%  **Racial composition**:  **Other socio-demographics**: NR  **Co morbidities**: NR  **Prior episode of pain if acute**: NR  **Prior CAM intervention**: NR  **Prior surgery related to current complaint**: NR | **Cause of Pain**:  NP  **Duration of Pain**:  Chronic (3 mo- 2yrs)  **Severity of pain (Grading)**:  NR  **Co-interventions**: None | **Groups**  **IG1** (n = 50) –acupuncture: acupuncture at ashi points and then warm needle; 15 min/2 wks  **Drop outs**: C=0  CG (n=50)-Spinal manipulation: NR; 1tx/wk, 3-4wks  **Drop outs**: C=0 | **Outcomes:**  **Pain**: VAS (0-10 cm), based on ITT  **Disability**: NR  **Results:** mean (SD)  Baseline: IG=8.84(1.81), CG=8.81(1.82)  **Immediate post tx**: NR  **Short term:** IG=4.46(3.11), CG=4.43(2.51) | **Outcome**  **instruments**:  **QoL/ well being**: SF-36  **Other**: NR  **Results:**  **Baseline**: NR  **immediate post tx**: NR  **1 month:** NR  **Short term**: NR  **intermediate**: NR  **Long term**: NR  **Harms**: NR | |
| **Coan, 1981[**[**37**](#_ENREF_37)**] USA** | **Trial Design:** RCT  **Tx duration**: >3 wks  **Fu duration** (last assessment): immediate post-tx  N screened: NR  N randomized:30  N completed tx: 30  N attended last fu: 30  **Inclusion**: NP (>=6mo), naive acu tx, no diabetes, infection or cancer  **Exclusion**: NR | **Mean age (SD/range)**:  Range27-74, 49.3 yrs total  **% of female**: 73.3%  **Racial composition**:  **Other socio-demographics**: NR  **Co morbidities**: NR  **Prior episode of pain if acute**: 11.5 hrs/d  **Prior CAM intervention**: naive acu tx  **Prior surgery related to current complaint**: not more than 2 previous neck surgeries | **Cause of Pain**:  Chronic, NP  **Duration of Pain**:  >=6 mo; mean (8.05yrs)  **Severity of pain (Grading)**:  5.7 on VAS 10  **Co-interventions**: None | **Groups**  **IG1** (n = 15) –acupuncture: acupoints selection varied from pts to pts, and d to d in the same pt. E-acu and moxibustion were used in some pts; 3-4tx/wk  **Drop outs**: 0  CG (n=15)-waitlist: no treatment  **Drop outs**: 0 | **Outcomes:**  **Pain**: VAS (0-10 cm)  **Disability**: NR  **Results:**  **Immediate post tx**: 12 of 15 of the tx group felt improved; 2 of 15 of the control got slight improvement  **Short term: NR** | **Outcome**  **instruments**:  **QoL/ well being**: NR  **Other**: NR  **Results:**  **Baseline**: NR  **immediate post tx**: NR  **Short term**: NR  **intermediate**: NR  **Long term**: NR  **Harms**: NR | |
| **Acupuncture for LBP** |  |  |  |  |  |  | |
| **Miyazaki, 2009 [**[**39**](#_ENREF_39)**] USA** | **Trial Design:** RCT  **Tx duration**: 21 days  **Fu duration** (last assessment): 3 mons  N screened: NR  N randomized:160  N completed tx: 156  N attended last fu: 143  **Inclusion**: chronic low back pain with duration >= 6 months and age 25–75 years;  **Exclusion**:  contraindications to acupuncture; such as anticoagulation with phenprocoumon or warfarin; coagulation disorders or thrombocytopenia; poor ﬂuency in German language; insufﬁcient adher- ence; recent surgical treatment; and herniated vertebral discs, either minor herniations of less than 6 months’ duration or major herniations of any duration. | **Mean age (SD/range)**:  50.7 yrs, (range, 31–73 years)  **% of female**: 33%  **Racial composition**: NR  **Other socio-demographics**:  Employment, marriage, unable to work  **Co morbidities**: NR  **Prior episode of pain if acute**: NR  **Prior CAM intervention**: NR  **Prior surgery related to current complaint**: NR | **Cause of Pain**:  Non specific  **Duration of Pain**:  11.3 ( 8.4) mons  **Severity of pain (Grading)**:  **Co-interventions**: NR | **Groups**  **IG** (n = 80–acupuncture + rehabilitation: received acupuncture twice weekly, 10 sessions (in 21 days) as one therapeutic course, each session varied between 30 and 40 minutes. standardized 21-day inpatient rehabilitation program according to current German guidelines.  **Drop outs**: A = 1, B =0,C=5  **CG** (n = 80–rehabilitation: a standardized 21-day inpatient rehabilitation program according to current German guidelines.  **Drop outs**: A = 3, B =0,C=8 | **Outcomes:**  **Pain**: VAS (0-100mm),  Not based on ITT  **Disability**: NR  Not based on ITT  **Results-Baseline:** mean (SD)  Pain**:** NR  Disability: NR  **Immediate post tx**:  Pain**:** NR  Disability:NR  **Short term**:  Pain**:**NR  Disability: NR | **Outcome**  **instruments**:  **QoL/ well being**: SF-36  **Other**: NR  **Results:**  **Baseline**: NR  **immediate post tx**: NR  **Short term**: NR  **intermediate**: NR  **Long term**: NR  **Harms**: NR | |
| **Cherkin, 2009[**[**40**](#_ENREF_40)**] USA** | **Trial Design:** RCT  **Tx duration**: 8 wks  **Fu duration** (last assessment): 44 wks  N screened: 2605  N randomized:638  N completed tx: 638  N attended last fu: 606  **Inclusion**: ages of 18-70 years, with CLBP for >= 3 months  **Exclusion**: specific cause of pain | **Mean age (SD/range)**:  18-70 (mean±SD 47±13)  yrs  **% of female**: 62% totally  **Racial composition**: NR  **Other socio-demographics**:  University Education (53%), Married (59%)  **Co morbidities**: NR  **Prior episode of pain if acute**: NR  **Prior CAM intervention**: no acu tx  **Prior surgery related to current complaint**: NR | **Cause of Pain**:  Non specific  **Duration of Pain**:  >=3mths  **Severity of pain (Grading)**:  NR  **Co-interventions**: a self-care book with information on managing flare-ups, exercise, and life-style modifications. | **Groups**  **IG 1**(n = 157–Individualized acupuncture: 74 distinct points were used, half on the “Bladder meridian” that includes points on the back and legs, average 10.8 (5-20) points choosed; with a depth of 30mm; 18min/10 x/7 wks(2 x/wk for 3 wks followed 1 x/wk for 4 wks)/De Qi  **Drop outs**: C=10, D=16  **IG2** (n =158–Standardized acupuncture: included 8 acupoints commonly used for CLBP (Du 3, Bladder 23- bilateral, low back ashi point, Bladder 40-bilateral, Kidney 3-bilateral) on the low back and leg; other same as IG1  **Drop outs**: C=6,D=11  **CG1** (n = 162–[Sham] a toothpick in a needle guidetube, points the same, non-penetration; no deqi, other same as IG1  **Drop outs**: C=3,D=10 | **Outcomes:**  **Pain**: NR  **Disability**: RMQ24  **Results:** mean (SD)  **Immediate post tx**:  Disability: IG1+IG2 = 6.35(5.5), CG1 = 5.4 (4.9)  **Short term:** Disability: IG1+IG2 = 6.75(5.65), CG1 = 6.4 (6)  **Intermediate term**: Pain**:** Disability: IG1+IG2 = 6(5.6), CG1 = 6.2 (5.8) | **Outcome**  **instruments**:  **QoL/ well being**: NR  **Other**: NR  **Results:**  **Baseline**:  **immediate post tx**: NR  **Short term**: NR  **intermediate**: NR  **Long term**: NR  **Harms**: 11 pts had mild side-effects | |
| **Haake, 2007[**[**41**](#_ENREF_41)**] Germany** | **Trial Design:** RCT  **Tx duration**: 7 wks  **Fu duration** (last assessment): 20 wks  N screened: 1802  N randomized:1162  N completed tx: 1117  N attended last fu: NR  **Inclusion**: ages >=18 years, with LBP for >= 6 months  **Exclusion**: trauma or systemic disease, spinal surgery, infections or tumor, other ongoing tx, not naive acu tx | **Mean age (SD/range)**:  18-86 (mean±SD 50±15) yrs  **% of female**: 50.3%  **Racial composition**: NR  **Other socio-demographics**: **NR**  **Co morbidities**: NR  **Prior episode of pain if acute**: NR  **Prior CAM intervention**: NR  **Prior surgery related to current complaint**: NR | **Cause of Pain**:  N-S, chronic, LBP  **Duration of Pain**:  >=6mths (mean±SD 57.24±60 mths), chronic,  **Severity of pain (Grading)**:  (67.77±13.91) on CPGS 100  **Co-interventions**: NASAID (<=2days/wk ) | **Groups**  **IG** (n =387–acupuncture: 14-20 needles, 0.25mm×40mm, depth of 5-40mm, “deqi”, 30min, 10tx/5wks  **Drop outs**: C = 10  **CG** (n = 387–sham acu: depth 1-3mm, no “deqi”,. avoiding all known verum points or meridians; needles, superficial, points not the same Other the same as IG.  **Drop outs**: C = 11 | **Outcomes:**  **Pain**: CPGS 100  **Disability:** HFAQ (lower better) 100  Results: mean (SD)  **Immediate post tx**:  Pain**:** IG = 48.6(18.5), CG = 51(18.7)  Disability**:** IG = 64(21.1), CG = 61.3(20.8)  **Short term:** Pain**:** IG = 45.4(19.4), CG = 48.5(19.5)  Disability**:** IG = 65.4(22.9), CG = 61.3(22.7)  **Intermediate term**: Pain**:** IG = 40.2(22.5), CG = 43.3(23)  Disability**:** IG = 66.8(23.1), CG = 62.2(23) | **Outcome**  **instruments**:  **QoL/ well being**: NR  **Other**: NR  **Results:**  **Baseline**:  **immediate post tx**: NR  **Short term**: NR  **intermediate**: NR  **Harms**: NR | |
| **Itoh, 2006[**[**42**](#_ENREF_42)**] Japan** | **Trial Design:** RCT  **Tx duration**: 3 wks  **Fu duration** (last assessment): 0.5 mo  N screened: 26  N randomized:26  N completed tx: 19  N attended last fu: 19  **Inclusion**: ages >=65 years, with LBP (>=6mo); leg pain if minor severity in comparison to back pain  **Exclusion**: trauma or systemic disease, spinal surgery, infections or tumor, other ongoing tx, not naive acu tx | **Mean age (SD/range)**:  65-91 (mean±SD 76.01±8.37) yrs  **% of female**: 65.4%  **Racial composition**: NR  **Other socio-demographics**: **NR**  **Co morbidities**: NR  **Prior episode of pain if acute**: NR  **Prior CAM intervention**: NR  **Prior surgery related to current complaint**: NR | **Cause of Pain**:  S, chronic, LBP  **Duration of Pain**:  >=6mths (mean±SD 57.24±60 mths), chronic,  **Severity of pain (Grading)**:  (6.69±1.30) on VAS 10cm  **Co-interventions**: Medication (as usual) | **Groups**  **IG** (n =13–acupuncture: This was identified in accessible muscles ideally by the presence of a tender taut band, patient recognition of pain, and local twitch response.; bilaterally, 3.6 needles, 0.2mm×50mm, depth of 10-40 mm, “deqi”, 10min, 1tx/wk for 3wks  **Drop outs**: 3  **CG** (n = 13–sham acu: The other points, blunt needles, non-penetration; no “deqi”, Other the same as IG.  **Drop outs**: 4 | **Outcomes:**  **Pain**: VAS 100mm  **Disability:** RMQ  **Results: mean (SD)**  **Immediate post tx**:  IG = 27.3(13.5), CG = 69.6(10.9)  Disability**:** IG = 3.3(1.5), CG = 8.6(3.1)  **Short term:** IG = 49.5(18.8), CG = 68.3(11.4)  **Intermediate term**: NR | **Outcome**  **instruments**:  **QoL/ well being**: NR  **Other**: NR  **Results:**  **immediate post tx**: NR  **Short term**: NR  **intermediate**: NR  **Harms**: NR | |
| **Inoue, 2006[**[**43**](#_ENREF_43)**] Japan** | **Trial Design:** RCT  **Tx duration**: single tx  **Fu duration** (last assessment): immediately post tx  N screened: NR  N randomized:31  N completed tx: 31  N attended last fu: NR  **Inclusion**: ages >=18 years, with LBP  **Exclusion**: trauma or systemic disease, spinal surgery, infections or tumor, other ongoing tx, not naive acu tx | **Mean age (SD/range)**:  69.03±7.17 yrs  **% of female**: 32.6%  **Racial composition**: NR  **Other socio-demographics**: **NR**  **Co morbidities**: NR  **Prior episode of pain if acute**: NR  **Prior CAM intervention**: NR  **Prior surgery related to current complaint**: NR | **Cause of Pain**:  S, chronic, LBP, lumbar vertebral arthritis  **Duration of Pain**:  (mean±SD 83.51±42.76 mths), chronic,  **Severity of pain (Grading)**:  (6.1±1.1) on VAS 10cm  **Co-interventions**: NR | **Groups**  **IG** (n =15–acupuncture: The point most painful, only LBP in a limited area, which was exacerbated in particular postures ; 1needles, 0.18mm×40mm, depth of 20 mm, “deqi”, 1tx totally  **Drop outs**: 0  **CG** (n = 16–sham acu: The same point, tube without needle, non-penetration; no “deqi”, Other the same as IG.  **Drop outs**: 0 | **Outcomes:**  **Pain**: VAS 100mm  **Disability:** NR  **Results: mean (SD)**  **Immediate post tx**:  Pain**:** IG = 47(7), CG = 55(13)  **Short term:** NR  **Intermediate term**: NR | **Outcome**  **instruments**:  **QoL/ well being**: NR  **Other**: NR  **Results:**  **immediate post tx**: NR  **Short term**: NR  **intermediate**: NR  **Harms**: NR | |
| **Brinkaus, 2006[**[**44**](#_ENREF_44)**] Germany** | **Trial Design:** RCT  **Tx duration**: 8 wks  **Fu duration** (last assessment): 44 wks  N screened: 301  N randomized:297  N completed tx: 297  N attended last fu: NR  **Inclusion**: ages of 40 - 75 years, with CLBP for >= 6 months, pain (>=4 on VAS 10cm)  **Exclusion**: protrusion or prolapsed of 1 or more intervertebral disc with concurrent neurological symptoms, radicular pain, prior vertebral surgery, other specific cause of pain | **Mean age (SD/range)**:  40-75 (mean±SD 59±9)yrs  **% of female**: 67.8% totally  **Racial composition**: NR  **Other socio-demographics**:  marital status, education  **Co morbidities**: NR  **Prior episode of pain if acute**: NR  **Prior CAM intervention**: no acu tx  **Prior surgery related to current complaint**: NR | **Cause of Pain**:  Non specific  **Duration of Pain**:  >=6mths (mean±SD 176.4±133.2 mths)  **Severity of pain (Grading)**:  >=4 (6.48±1.4) on VAS 10cm  **Co-interventions**: NSAID, other additional therapies were prohibited | **Groups**  **IG** (n = 146–acupuncture: >=4 local points: bladder 20 to 34; bladder 50 to 54; gallbladder 30; governing vessel 3, 4, 5, and 6; and extraordinary points Huatojiaji and Shiqizhuixia. at least 2 distant points: small intestine 3; bladder 40, 60, and 62; kidney 3 and 7; gallbladder 31, 34, and 41; liver 3; and governing vessel 14 and 20. Ear and trigger points ; 30 min/12 x/8 wks (2x/wk for first 4wks followed by 1x/wk in 4wks)/De Qi  **Drop outs**: C=10  **CG1** (n =73–Sham acupuncture: At least 6 of 10 predefined non-acupuncture points, Superficial insertion., other the same as IG  **Drop outs**: C=7  **CG2** (n = 79–waitlist: no tx  **Drop outs**: C=5 | **Outcomes:**  **Pain**: VAS (100mm),  **Disability**: PDI  **Results:** mean (SD)  **Immediate post tx**:  Pain**:** IG = 34.5(28.5), CG1 = 43.7(29.8), CG2 = 58.6 (25.1)  Disability: IG = 18.8 (13.1), CG1 = 21.5 (13.2), CG2 = 27.1(14.1)  **Short term:** Pain**:** IG = 34.4(29.8), CG1 = 42.1(30.3), Disability: IG = 19.3(13.9), CG1 = 21.4 (15.6)  **Intermediate term**: Pain**:** IG = 39.2 (29.2), CG1 = 44.9 (30.4),  Disability: IG = 19(13.4), CG1 = 23 (15) | **Outcome**  **instruments**:  **QoL/ well being**: SF-36 (physical health), (mental health)  **Other**: NR  **Results:**  **Baseline**:  **immediate post tx**: NR  **Short term**: NR  **intermediate**: NR  **Long term**: NR  **Harms**: NR | |
| **Itoh, 2004[**[**45**](#_ENREF_45)**] Japan** | **Trial Design:** RCT  **Tx duration**: 6 wks  **Fu duration** (last assessment): 3 mo  N screened: NR  N randomized:35  N completed tx: 27  N attended last fu: NR  **Inclusion**: ages of 20 - 45 years, with LBP for >= 6 months, no radiation, pain (>=5 on VAS10)  **Exclusion**: Major trauma or systemic disease, other ongoing tx | **Mean age (SD/range)**:  70.1-73.8 yrs  **% of female**: 28.6%  **Racial composition**: NR  **Other socio-demographics**: **NR**  **Co morbidities**: spondylosis (n=8), osteoporosis (n=5), compression fracture(n=3)  **Prior episode of pain if acute**: NR  **Prior CAM intervention**: NR  **Prior surgery related to current complaint**: NR | **Cause of Pain**:  LBP  **Duration of Pain**:  >= 6 mons, chronic,  **Severity of pain (Grading)**:  VAS (0-10)，  >=5  **Co-interventions**: Analgesic | **Groups**  **IG** (n =10–deep acupuncture: needles 0.2mm×50mm, depth of 20mm, “deqi”, 10min, 2tx/3wks, 2wks interval  **Drop outs**: A = 1  **CG** (n = 12–superficial acu: depth 3mm, no “deqi”,. Other the same as IG.  **Drop outs**: A = 3 | **Outcomes:**  **Pain**: VAS (100mm),  Not based on ITT?  **Disability**: NR  **Results:** mean (SD)  **Immediate post tx**:  Pain**:** IG = 43.2(23.4), CG = 48.3(27.9)  **Short term:** Pain**:** IG = 56.8(25.1), CG = 50.1(32.5)  **Intermediate term**: NR | **Outcome**  **instruments**:  **QoL/ well being**: NR  **Other**: NR  **Results:**  **Baseline**:  **immediate post tx**: NR  **Short term**: NR  **Harms**: NR | |
| **Molsberger, 2002[**[**46**](#_ENREF_46)**] Germany** | **Trial Design:** RCT  **Tx duration**: 4 wks  **Fu duration** (last assessment): 3 mo  N screened: NR  N randomized:186  N completed tx: 174  N attended last fu: 186  **Inclusion**: ages of 20 - 60 years, with LBP for >= 6 wks, pain (>=5 on VAS 10cm)  **Exclusion**:  (1) speciﬁc causes of back pain (e.g., cancer, fractures, spinal stenosis, and infections); (2) complicated back problems (e.g., sciatica, chronic spondylitis, prior back surgery, medicolegal issues) | **Mean age (SD/range)**:  20-60 (mean±SD 50±7) yrs  **% of female**: 47.8%  **Racial composition**: NR  **Other socio-demographics**: NR  **Co morbidities**: NR  **Prior episode of pain if acute**: NR  **Prior CAM intervention**: NR  **Prior surgery related to current complaint**: NR | **Cause of Pain**:  Non specific  **Duration of Pain**:  >=6mths (mean±SD 118.8±93.6 mths)  **Severity of pain (Grading)**:  >=5 (6.6±1.5) on VAS 10cm  **Co-interventions**: Combination of conventional orthopedic therapy, NSAID | **Groups**  **IG** (n = 65–Verum acupuncture + orthopedic therapy: Lumbar region: urinary bladder 23, 25, and gallbladder 30; Lower extremity: were urinary bladder 40, 60 and gallbladder 34; TrPs: four points of maximum pain ‘Ahshi points’; bilaterally, 16 points, depth of 10-100 mm, "Deqi"; 30 min, 3tx/wk for 4 wks  **Drop outs**: 18  **CG1** (n = 61–sham acu + orthopedic therapy: non-acupuncture points, superficially, on back. Other the same as IG.  **Drop outs**: 20  **CG2** (n = 60–orthopedic therapy: exercise, back school, infrared heat therapy, 50 mg diclofenac on demand  **Drop outs**: 24 | **Outcomes:**  **Pain**: VAS (100mm),  **Disability**: NR  **Results-Baseline:** mean (SD)  Pain**:** IG = 68(17), CG1 = 64 (11), CG2 = 67 (14)  **Immediate post tx**:  Pain**:** IG = 26(21), CG1 = 36 (19), CG2 = 36 (19)  **Short term:** Pain**:** IG = 23 (20), CG1 = 43(23), CG2 = 43(23)  **Intermediate term**: NR | **Outcome**  **instruments**:  **QoL/ well being**: SF-36 (physical health), (mental health)  **Other**: NR  **Results:**  **Baseline**:  physical health**:** IG = 42 (11), CG1 = 40 (12), CG2 = 42 (11)  mental health**:** IG = 37 (11), CG1 = 38 (10), CG2 = 40 (10)  **immediate post tx**: NR  **Short term**: NR  **intermediate**: NR  **Long term**: NR  **Harms**: NR | |
| **Leibing, 2002[**[**47**](#_ENREF_47)**] Germany** | **Trial Design:** RCT  **Tx duration**: 12 wks  **Fu duration** (last assessment): 9 mo  N screened: 208  N randomized:131  N completed tx: 114  N attended last fu: 114  **Inclusion**: ages of 18-65 years, with LBP for >= 6 months, no radiation pain  **Exclusion**: abnormal neurological status, concomitant severe disease, psychiatric, current psychotherapy, rheumatic, inflammatic disease | **Mean age (SD/range)**:  18-65 (mean±SD 48.1±9.7) yrs  **% of female**: 58%  **Racial composition**: NR  **Other socio-demographics**: **NR**  **Co morbidities**: NR  **Prior episode of pain if acute**: NR  **Prior CAM intervention**: NR  **Prior surgery related to current complaint**: IG1=4, IG2=2, CG=4 | **Cause of Pain**:  N-S, LBP  **Duration of Pain**:  >=6mths (mean±SD 115.2±98.4 mths), chronic  **Severity of pain (Grading)**:  (5.2±1.9) on VAS 10cm  **Co-interventions**: NSAID as usual | **Groups**  **IG1** (n =40–acu + physio: 21 fixed body acupoints (nine bilateral, two single points): GV3,4,BL23,25,31,32,40,60,GB34,SP6, Yautungdien；and 6 on the ear (alternately on one ear); needles 0.3mm×40mm, depth of 10-30mm, “deqi”, 30min, 5tx/wk for first 2wks followed by 1tx/wk for 10 wks  **Drop outs**: C = 7  **IG2** (n =45–physio: according to Bruggar concept, aim was to remove a muscle imbalance through special training of proper posture and motion; totally 26 sessions, 30min/tx for 12 wks  **Drop outs**: C = 14  **CG** (n = 45–sham acu + physio: inserted superficially, 10–20 mm distant to the verum-acupoints, outside the meridians, depth 3mm, no “deqi”,. Other the same as IG.  **Drop outs**: C = 14 | **Outcomes:**  **Pain**: VAS (100mm),  **Disability**: PDI  **Results:** mean (SD)  **Immediate post tx**:  Pain**:** IG1 = 21(24.5), IG2 = 32(22), CG = 32(24.5)  Disability**:** IG1 = 11.3(16.2), IG2 = 15.8(10.5), CG = 15.8(10.6)  **Short term:** NR  **Intermediate term**: Pain**:** IG1 = 31(18), IG2 = 35(22), CG = 35(24.5)  Disability**:** IG1 = 16.2(14.5), IG2 = 22.6(10), CG = 17(12) | **Outcome**  **instruments**:  **QoL/ well being**: NR  **Other**: NR  **Results:**  **Baseline**:  **immediate post tx**: NR  **Short term**: NR  **Harms**: painfulness of acu (2), problem with circulation (1) | |
| **Hasegawa, 2013[**[**48**](#_ENREF_48)**] Brazil** | **Trial Design:** RCT  **Tx duration**: 4 wks  **Fu duration** (last assessment): 3 mo  N screened: NR  N randomized:80  N completed tx: 80  N attended last fu: 80  **Inclusion**: ages of 18-65 yrs, with N-S LBP  **Exclusion**: systemic disaeases, contra-indications to acu, previous acu tx, conflicting or ongoing tx | **Mean age (SD/range)**:  18-65 (mean±SD 45.45±10.48) yrs  **% of female**: 63.8%  **Racial composition**: NR  **Other socio-demographics**:  NR  **Co morbidities**: NR  **Prior episode of pain if acute**: NR  **Prior CAM intervention**: NR  **Prior surgery related to current complaint**: NR | **Cause of Pain**:  N-S, acute  **Duration of Pain**:  <1mth (mean±SD 15.25±11.35 days)**Severity of pain (Grading)**:  4-8 points (6.61±1.42) on VAS 10cm **Co-interventions**: Medication (50 mg sodium diclofenac every 8 h) | **Groups**  **IG** (n = 40 Acu: basic points D, H and I and kidney, bladder and liver points of Yamamoto’s metho; Bilaterally/12 points /needles (0.20mm×13mm)/3-5mm; 30 min/5 x/4 wks(2x/wk for first wk followed 1x/wk for 3 wks)/ deqi NR  **Drop outs**: 0  **CG** (n = 40–sham acupuncture: The same points, non-penetration, just handle contact;. Other the same as IG.  **Drop outs**: 0 | **Outcomes:**  **Pain**: VAS (0-10cm),  **Disability**: RMQ  **Results:** mean (SD)  **Immediate post tx**:  Pain**:** IG = 1.74(2.07), CG = 3 (2.41)  **Short term:** NR | **Outcome**  **instruments**:  **QoL/ well being**: SF-36  **Other**: NR  **Results:**  **Baseline**:  **immediate post tx**: NR  **Short term**: NR  **intermediate**: NR  **Long term**: NR  **Harms**: NR | |
| **Su, 2010[**[**50**](#_ENREF_50)**] China** | **Trial Design:** RCT  **Tx duration**: 1 tx only  **Fu duration** (last assessment): immediate post-tx  N screened: NR  N randomized:60  N completed tx: 60  N attended last fu: 60  **Inclusion**: LBP (<1 mo), age >=18 yrs  **Exclusion**: tx with anticoagulants or corticosteroids, previous acu, severe concomitant disease | **Mean age (SD/range)**:  39.6yrs  **% of female**: 41.67%  **Racial composition**: NR  **Other socio-demographics**:  NR  **Co morbidities**: NR  **Prior episode of pain if acute**: NR  **Prior CAM intervention**: No acu tx  **Prior surgery related to current complaint**: NR | **Cause of Pain**:  acute  **Duration of Pain**:  Mean, 16d  **Severity of pain (Grading)**:  NR  **Co-interventions**: NR | **Groups**  **IG** (n = 30–Acu: wrist-ankle method; Xia5, Xia6; deqi was not elicited; 1 tx totally  **Drop outs**: 0  **CG** (n = 30–sham: non-penetration sham needling at identical acupuncture points as IG  **Drop outs**:0 | **Outcomes:**  **Pain**: VAS (100mm),  **Disability**: NR  **Results:** mean (SD)  **Immediate post tx**:  Pain**:** IG = 35.30(10.89), CG = 46.73 (9.26)  **Short term:** NR | **Outcome**  **instruments**:  **QoL/ well being**: NR  **Other**: NR  **Results:**  **Baseline**:  **immediate post tx**: NR  **Short term**: NR  **intermediate**: NR  **Long term**: NR  **Harms**: 3 pts reported mild hypodermal bleeding | |
| **Kennedy, 2008[**[**51**](#_ENREF_51)**] Ireland** | **Trial Design:** RCT  **Tx duration**: 4-6 wks  **Fu duration** (last assessment): 3 mo  N screened: 55  N randomized:48  N completed tx: 45  N attended last fu: 40  **Inclusion**: ages of 18-70 yrs, with N-S LBP  **Exclusion**: red flags (defined by CSAG), contra-indications to acu, previous acu tx, conflicting or ongoing tx | **Mean age (SD/range)**:  45.55±11.14 yrs  **% of female**: 52.1%  **Racial composition**: NR  **Other socio-demographics**:  NR  **Co morbidities**: NR  **Prior episode of pain if acute**: NR  **Prior CAM intervention**: NR  **Prior surgery related to current complaint**: NR | **Cause of Pain**:  N-S, acute  **Duration of Pain**:  <3mths  **Severity of pain (Grading)**:  VAS (0-10)，  5.94±0.59  **Co-interventions**: Medication, normal activities | **Groups**  **IG** (n = 24–Acu: GV3, GV4, BL23, BL25, GB29, GB30, GB31, GB34, BL36, BL37, BL40, BL56, BL60; Bilaterally/8-13 points /needles (0.25mm×40mm)/5-30mm; 30 min/3-12 x/4-6 wks(1-2x/wk)/De Qi  **Drop outs**: B =3  **CG** (n = 24–sham acupuncture: The same points, non-penetration;. Other the same as IG.  **Drop outs**: B =8 | **Outcomes:**  **Pain**: VAS (0-10cm),  Not based on ITT?  **Disability**: NR  **Results:** mean (SD)  **Immediate post tx**:  Pain**:** IG = 2.73(0.49), CG = 3.63 (0.61)  **Short term:** NR | **Outcome**  **instruments**:  **QoL/ well being**: SF-36  **Other**: NR  **Results:**  **Baseline**:  **immediate post tx**: NR  **Short term**: NR  **intermediate**: NR  **Long term**: NR  **Harms**: NR | |
| **Zaringhalam, 2010[**[**52**](#_ENREF_52)**] Iran** | **Trial Design:** RCT  **Tx duration**: 5 wks  **Fu duration** (last assessment): 5 wks  N screened: 125  N randomized: 84  N completed tx: 84  N attended last fu: 80  **Inclusion**: LBP (>=6 mo),men aged 50-60 yrs; no acupuncture tx in past 6 mths;  **Exclusion**: trauma or systemic disorders, conflicting or ongoing co-interventions, prior vertebral surgery, disc protrusion or prolapsed, infections, fracture | **Mean age (SD/range)**:  50-60; 54.45 yrs  **% of female**: 0%  **Racial composition**: NR  **Other socio-demographics**: Asian  **Co morbidities**: NR  **Prior episode of pain if acute**: NR  **Prior CAM intervention**: NR  **Prior surgery related to current complaint**: NR | **Cause of Pain**:  N-S, Chronic, LBP  **Duration of Pain**:  Mean=6.93yrs  **Severity of pain (Grading)**: NR  **Co-interventions**: exercise, analgesics | Groups  IG (n = 21–E-Acu: BL23, BL25, BL28, BL32, BL60, GB30, GB34; 4-6 Hz, 0.5 ms; 10-12 points, bilaterally; 20-25 min, 2 tx/wk for 5 wks, De Qi  Drop outs: 1  IG2 (n = 21–Baclofen: oral baclofen 30mg/d  Drop outs: 1  IG3 (n = 21–E-acu + baclofen: same as IG1 and IG2.  Drop outs: 1  CG (n = 21–notreatment:  Drop outs: 1 | **Outcomes:**  **Pain**: VAS (100mm),  **Disability**: RMQ  **Results:** mean (SD)  **Immediate post tx**:  Pain**:** IG1 = 47(19.1), IG2 = 61.9(22.3), IG3= 40.1(13.3), CG= 64.3(23.8)  Disability**:** IG1 = 6.4(2.9), IG2 = 8.8(3.8), IG3= 5.7(1.4), CG= 9.8(3.9)  **Short term:**  Pain**:** IG1 = 50.1(20.3), IG2 = 63.7(24.4), IG3= 47.3(14.1), CG= 64.2(25.5)  Disability**:** IG1 = 7.2(3.1), IG2 = 9.5(4.1), IG3= 5.8(1.4), CG= 9.9(4.6) | **Outcome**  **instruments**:  **QoL/ well being**: NR  **Other**: NR  **Results:**  **Baseline**:  **immediate post tx**: NR  **Short term**: NR  **intermediate**: NR  **Long term**: NR  **Harms**: NR | |
| **Witt, 2006[**[**53**](#_ENREF_53)**] Germany** | **Trial Design:** RCT  **Tx duration**: 3 mo  **Fu duration** (last assessment): immediate post-tx  N screened: 11630  N randomized: 2840  N completed tx: 2840  N attended last fu: 2518  **Inclusion**: ages >=18 years, with LBP for >= 6 mths  **Exclusion**: specific LBP, lumbar surgery, previously acu tx, other ongoing tx within past 3 mo | **Mean age (SD/range)**:  IG=53.1(13.5), CG=52.6(13.2) yrs  **% of female**: IG=57.7%, CG=56.9%  **Racial composition**: NR  **Other socio-demographics**: **NR**  **Co morbidities**: NR  **Prior episode of pain if acute**: NR  **Prior CAM intervention**: naive acu tx  **Prior surgery related to current complaint**: NR | **Cause of Pain**:  N-S, Chronic, LBP  **Duration of Pain**:  chronic, IG=7.2(8), CG=7.2(7.8) yrs  **Severity of pain (Grading)**:  NR  **Co-interventions**: usual care | **Groups**  **IG** (n =1451–acu: needles 0.25mm×40mm, depth of 20mm, “deqi”, 30min, 3 mo tx phase, max 15 tx, 74% received 5-10 tx, 21% received >10 tx, 5% received < 5tx  **Drop outs**: C=88, D=130  **CG** (n = 1390–no treatment: Other the same as IG.  **Drop outs**: C=130, D=193 | **Outcomes:**  **Pain**: BP score  **Disability**: NR  **Results:** mean (SD)  **Immediate post tx**:  Pain**:** IG = 17(12), CG = 24(13)  **Short term:** Pain**:** NR  **Intermediate term**: NR | **Outcome**  **instruments**:  **QoL/ well being**: NR  **Other**: NR  **Results:**  **Baseline**:  **immediate post tx**: NR  **Short term**: NR  **Harms**: NR | |
| **Coan, 1980[**[**55**](#_ENREF_55)**] USA** | **Trial Design:** RCT  **Tx duration**: NR  **Fu duration** (last assessment): immediate post-tx  N screened: NR  N randomized:50  N completed tx: NR  N attended last fu: NR  **Inclusion**: LBP (>=6 mo), no previous acu tx, no history of diabetes, infection or cancer, not more than 2 back surgeries  **Exclusion**: NR | **Mean age (SD/range)**:  47 yrs  **% of female**: IG=43.5%, CG=50%  **Racial composition**: NR  **Other socio-demographics**:  NR  **Co morbidities**: NR  **Prior episode of pain if acute**: NR  **Prior CAM intervention**: No acu tx  **Prior surgery related to current complaint**: NR | **Cause of Pain**:  NR, chronic  **Duration of Pain**:  IG=8.2, CG=12.6 yrs  **Severity of pain (Grading)**:  NR  **Co-interventions**: NR | **Groups**  **IG** (n = 25–Acu: performed according to the classical oriental meridian theory of promoting healing by stimulating the energy flow in the body. In some pts E-acu was used.  **Drop outs**: NR  **CG** (n = 25–waitlist: same as IG.  **Drop outs**: NR | **Outcomes:**  **Pain**: VAS (100mm),  **Disability**: NR  **Results:** mean (SD)  **Immediate post tx**:  Pain**:** IG = 28(20), CG = 47 (20)  **Short term:** NR | **Outcome**  **instruments**:  **QoL/ well being**: NR  **Other**: NR  **Results:**  **Baseline**:  **immediate post tx**: NR  **Short term**: NR  **intermediate**: NR  **Long term**: NR  **Harms**: NR | |
| **Itoh, 2009[**[**56**](#_ENREF_56)**] Japan** | **Trial Design:** RCT  **Tx duration**: 5 wks  **Fu duration** (last assessment): 10 wks  N screened: NR  N randomized:32  N completed tx: 25  N attended last fu: 26  **Inclusion**: LBP (>=6 mo), age>=60 yrs  **Exclusion**: if reveiving acu > 6mo, trauma or systemic disease; receiving conflicting or ongoing co-interventions | **Mean age (SD/range)**:  61-81 yrs  **% of female**: 37.5%  **Racial composition**: NR  **Other socio-demographics**:  NR  **Co morbidities**: NR  **Prior episode of pain if acute**: NR  **Prior CAM intervention**: NR  **Prior surgery related to current complaint**: NR | **Cause of Pain**:  N-S, chronic  **Duration of Pain**:  >6mo  **Severity of pain (Grading)**: NR  **Co-interventions**: no medications | Groups  IG (n = 8–Acu: BL23, BL25, BL32, BL40, BL60, GB30, GB34; bilaterally /needles (0.2mm×40mm)/ depth 10 mm; 30 min, 1 tx/wk for 5 wks, De Qi  Drop outs: 1  CG 1(n = 8–TENS: 15 min on most tender point and near side of point, same as IG.  Drop outs: 2  CG2(n = 8–Acu + TENS:  15 min of TENS plus 15 min TENS as IG, CG1  Drop outs: 2  CG3 (n = 8–medication: same as IG.  Drop outs: 1 | **Outcomes:**  **Pain**: VAS (10cm),  **Disability**: RMQ-24  **Results:** mean (SD)  **Immediate post tx**:  Pain**:** IG = 4.8(1.9), CG1 = 6.1(2.4), CG2= 3.66(0.8), CG3 = 5.8 (2.2)  **Short term: Immediate post tx**:  Pain**:** IG = 4.3(2.6), CG1 = 5.8 (2.3), , CG2= 4.92(1.03) | **Outcome**  **instruments**:  **QoL/ well being**: NR  **Other**: NR  **Results:**  **Baseline**:  **immediate post tx**: NR  **Short term**: NR  **intermediate**: NR  **Long term**: NR  **Harms**: NR | |
| **Grant, 1999[**[**57**](#_ENREF_57)**] UK** | **Trial Design:** RCT  **Tx duration**: 4 wks  **Fu duration** (last assessment): 3 mo  N screened: 81  N randomized:60  N completed tx: 60  N attended last fu: 60  **Inclusion**: LBP (>=6 mo), age >60 yrs  **Exclusion**: tx with anticoagulants or corticosteroids, previous acu ro TENS, severe concomitant disease | **Mean age (SD/range)**:  IG=75 rane(60-83), CG =72 range (60-90)yrs  **% of female**: IG=93.75%, CG=85.72%  **Racial composition**: NR  **Other socio-demographics**:  NR  **Co morbidities**: NR  **Prior episode of pain if acute**: NR  **Prior CAM intervention**: No acu tx  **Prior surgery related to current complaint**: NR | **Cause of Pain**:  N-S, chronic  **Duration of Pain**:  NR  **Severity of pain (Grading)**:  NR  **Co-interventions**: continue the analgesic as usual | **Groups**  **IG** (n = 32–Acu: using only points on the back, 6 (range 2-8) needles on average at each tx ; 2 tx of 20min/wk for 4 wks  **Drop outs**: 2  **CG** (n = 28–TENS: standard model using 50Hz with the intensity adjusted to suit the pts. Used at home; 30 min, max of 6 hrs/d, twice daily  **Drop outs**:1 | **Outcomes:**  **Pain**: VAS (10cm),  **Disability**: NR  **Results:** mean (SD)  **Immediate post tx**:  Pain**:** IG = 7.1(6.6), CG = 4.7 (4.4)  **Short term:** Pain**:** IG = 6(6.5), CG = 6.3 (6) | **Outcome**  **instruments**:  **QoL/ well being**: NR  **Other**: NR  **Results:**  **Baseline**:  **immediate post tx**: NR  **Short term**: NR  **intermediate**: NR  **Long term**: n=2: influenza and immobility  **Harms**: NR | |
| **Muller, 2005[**[**58**](#_ENREF_58)**] Australia** | **Trial Design:** RCT  **Tx duration**: 9 wks  **Fu duration** (last assessment): immediate post-tx  N screened: NR  N randomized:80  N completed tx: 73  N attended last fu: 44  **Inclusion**: ages >=17 yrs, with LBP pain (>13wks)  **Exclusion**: nerve root involvement, spinal anomalies, pathological conditions, previous spinal surgery | **Mean age (SD/range)**:  39yrs, range 29-46  **% of female**: 46.8%  **Racial composition**: NR  **Other socio-demographics**: socioeconomic  **Co morbidities**: NR  **Prior episode of pain if acute**: NR  **Prior CAM intervention**: NR  **Prior surgery related to current complaint**: NR | **Cause of Pain**:  Chronic mechanical LBP  **Duration of Pain**: > 13 wks  **Severity of pain (Grading)**:  NR  **Co-interventions**: NR | **Groups**  **IG** (n = 36–Acu: 8-10 needles local paraspinal intramuscular maximum pain areas, and 5 needles at distal meridians; needle (0.25 mm×50mm) with a depth of 20-50mm, 2 x/wk for 9 wks, De Qi  **Drop outs**: 14  **CG** (n =43–medication: celecoxib (200-400mg/d); or rofecoxib (12.5-25mg/d); or acetaminophen (500mg tablets 2-6 per d)  **Drop outs**: 21 | **Outcomes:**  **Pain**: VAS (0-10cm),  **Disability**: NDI  **Result:** mean (SD)  **Immediate post tx**:  Pain**:** IG = 3.9(3.23), CG = 3.9 (3.3)  Disabiltiy**:** IG = 19(24), CG = 20 (30)  **Short term:** NR | **Outcome**  **instruments**:  **QoL/ well being**: NR  **Other**: NR  **Results:**  **Baseline**:  **immediate post tx**: NR  **Harms**: mild side-effects | |
| **Wang, 2004[**[**59**](#_ENREF_59)**] China** | **Trial Design:** RCT  **Tx duration**: 5-7 d  **Fu duration** (last assessment): immediate post-tx  N screened: NR  N randomized:40  N completed tx: 37  N attended last fu: NR  **Inclusion**: ages >=18 yrs, with disc protrusion, with radiating pain (>2yrs)  **Exclusion**: NR | **Mean age (SD/range)**:  46yrs, range 20-59  **% of female**: 75%  **Racial composition**: NR  **Other socio-demographics**: **Asian**  **Co morbidities**: NR  **Prior episode of pain if acute**: NR  **Prior CAM intervention**: NR  **Prior surgery related to current complaint**: NR | **Cause of Pain**:  Chronic, mechanical conditions, disc prorusion  **Duration of Pain**: > 2yrs  **Severity of pain (Grading)**:  NR  **Co-interventions**: NR | **Groups**  **IG** (n = 23–E-Acu: huantiao, weizhong; G6805 type electric stimulator, bilaterally, 25 min, 1 tx/d for 7d De Qi  **Drop outs**: NR  **CG** (n =17–medication: Diclofenic 25mg/tablet; given post cibum at 50 mg tid for 5 d, orally  **Drop outs**: NR | **Outcomes:**  **Pain**: VAS (0-10cm),  **Disability**: NR  **Results-baseline:** mean (SD) Pain**:** IG = 4.95(1.4), CG = 5.03 (1.2)  **Immediate post tx**:  Pain**:** IG = 2.6(2.3), CG = 3.3 (2.5)  **Short term:** NR | **Outcome**  **instruments**:  **QoL/ well being**: NR  **Other**: NR  **Results:**  **Baseline**:  **immediate post tx**: NR  **1 week:**NR  **Short term**: NR  **intermediate**: NR  **Long term**: NR  **Harms**: NR | |
| **Giles, 2003[**[**32**](#_ENREF_32)**] Australia** | **Trial Design:** RCT  **Tx duration**: 9 wks  **Fu duration** (last assessment): immediate post-tx  N screened: 533  N randomized: 115  N completed tx: 69  N attended last fu: 62  **Inclusion**: ages >17 yrs, with uncomplicated mechanical spinal pain (>13 wks)  **Exclusion**: nerve root involvement, spinal abnormalies | **Mean age (SD/range)**:  25(8.1) yrs totally  **% of female**: 55.1% totally  **Racial composition**: NR  **Other socio-demographics**: **NR**  **Co morbidities**: NR  **Prior episode of pain if acute**: NR  **Prior CAM intervention**: NR  **Prior surgery related to current complaint**: NR | **Cause of Pain**:  N-S, chronic  **Duration of Pain**: > 13 wks  **Severity of pain (Grading)**:  NR  **Co-interventions**: NR | **Groups**  **IG** (n = 36–E-Acu: depth of 20-50mm, trigger points, De Qi  **Drop outs**: 14  **CG** (n =43–medication: anaesthetic had not been used previously  **Drop outs**: 12 | **Outcomes:**  **Pain**: VAS (0-10cm),  **Disability**: NR  **Results-baseline:** mean (SD) **Immediate post tx**:  Pain**:** IG = 7(5.2), CG = 5 (3.7)  **Short term:** NR | **Outcome**  **instruments**:  **QoL/ well being**: NR  **Other**: NR  **Results:**  **Baseline**:  **immediate post tx**: NR  **Harms**: mild side-effects | |
| **Giles, 1999[**[**33**](#_ENREF_33)**] Australia** | **Trial Design:** RCT  **Tx duration**: 3-4 wks  **Fu duration** (last assessment): immediate post-tx  N screened: NR  N randomized: 40  N completed tx: 36  N attended last fu: 36  **Inclusion**: age >=18yrs with uncomplicated mechanical spinal pain for minimum of 13 wks  **Exclusion**: nerve root involvement, spinal anomalies, pathology other than mild-moderate osteoarthritis, leg length inequality >9mm with postural scoliosis | **Mean age (SD/range)**:  IG= 46.5(9.6) ; CG= 35(14.1) yrs  **% of female**: 64.3% total **Racial composition**: NR  **Other socio-demographics**: **NR**  **Co morbidities**: NR  **Prior episode of pain if acute**: NR  **Prior CAM intervention**: NR  **Prior surgery related to current complaint**: None | **Cause of Pain**:  N-S, chronic  **Duration of Pain**: > 13 wks  **Severity of pain (Grading)**:  NR  **Co-interventions**: NR | **Groups**  **IG** (n = 20–E-Acu: near and far techniques as chosen by clinician; 2 tx/wk, 3-4 wks, De Qi  **Drop outs**: 4  **CG** (n =20–medication: enoxican (20 mg/d) and ranitidine (50 mg twice/d); 15-20 min/appointment, 3-4 wks  **Drop outs**: 0 | **Outcomes:**  **Pain**: VAS (0-10cm),  **Disability**: ODI  **Results-baseline:** mean (SD) **Immediate post tx**:  Pain**:** IG = 5.1(7.8), CG = 3.8 (4.8); Disability**:** IG = 24.5(26.6), CG = 20 (21.5)  **Short term:** NR | **Outcome**  **instruments**:  **QoL/ well being**: NR  **Other**: NR  **Results:**  **Baseline**:  **immediate post tx**: NR  **Harms**: mild side-effects | |
| **Yun, 2012 [**[**129**](#_ENREF_129)**]**  **China** | **Trial Design:** RCT  **Tx duration**: 7 weks  **Fu duration** (last assessment): 41 weks  N screened: 329  N randomized:187  N completed tx: 187  N attended last fu: 187  **Inclusion**: ages of 20 - 45 years, with LBP for >= 3 months  **Exclusion**:  (1) speciﬁc causes of back pain (e.g., cancer, fractures, spinal stenosis, and infections); (2) complicated back problems (e.g., sciatica, scoliologic > 40° curvature, chronic spondylitis, prior back surgery, medicolegal issues); (3) possible contraindications for acupuncture (4) conditions making treatment difﬁcult; (5) conditions that might confound treatment effects or interpretation of results (e.g., severe ﬁbromyalgia, rheumatoid arthritis, concurrent care from other providers); and (6) previous acupuncture treatment for any condition. | **Mean age (SD/range)**:  34 (11) yrs  **% of female**: 23%  **Racial composition**: NR  **Other socio-demographics**:  marital status, education  **Co morbidities**: NR  **Prior episode of pain if acute**: NR  **Prior CAM intervention**: 41% had used the medication in the past week  **Prior surgery related to current complaint**: NR | **Cause of Pain**:  Non specific  **Duration of Pain**:  >= 3 mons, Most (71%) pts reported at least 1 year of pain  **Severity of pain (Grading)**:  VAS (0-10)，  6.2  **Co-interventions**: None | **Groups**  **IG** (n = 64–Hegu acupuncture: 18 sessions (every other day for 3 wks and then twice weekly for 4 ks.), 20 mins, inserted into the muscle (to a depth of 20-30mm), true acupoints. operated to get the due sensation (called Diqi)  "Deqi". Hegu acupoint  **Drop outs**: A = 0, B =0,E=0  **CG1** (n = 60–standard acupuncture: eight acupuncture points that are commonly used for CLBP. Other the same as IG.  **Drop outs**: A = 0, B =0,E=0  **CG2** (n = 63–usual care:  participants received no study- related care—just the care, if any, that they and their physicians chose: mostly massage and physical therapy visits and continued use of medications (mostly nonsteroidal anti- inﬂammatory drugs). received a self-care book  **Drop outs**: A = 0, B =0,E=0 | **Outcomes:**  **Pain**: VAS (0-10cm),  Not based on ITT?  **Disability**: RMDQ, (0-24)  Not based on ITT?  **Results-Baseline:** mean (SD)  Pain**:** IG = 6.1 (1.8), CG1 = 6.3 (2.0), CG2 = 6.1 (1.7)  Disability: IG = 11.0 (3.0), CG1 = 10.7 (3.0), CG2 = 10.9 (3.0)  **Immediate post tx**:  NR  **1 weeks:**  Pain**:** IG = 4.6 (1.3), CG1 = 5.0 (1.4), CG2 = 5.6 (1.6)  Disability: IG = 5.7 (1.7), CG1 = 6.6 (1.5), CG2 = 8.8 (2.4)  **Short term:**NR  **Intermediate term**: NR  **10 Months (Long term):**  Pain**:** IG = 3.5 (1.0), CG1 = 3.9 (1.1), CG2 = 4.5 (1.2)  Disability: IG = 5.3 (1.6), CG1 = 6.5 (1.7), CG2 = 7.6 (2.2) | **Outcome**  **instruments**:  **QoL/ well being**: SF-36 (physical health), (mental health)  **Other**: NR  **Results:**  **Baseline**:  physical health**:** IG = 42 (11), CG1 = 40 (12), CG2 = 42 (11)  mental health**:** IG = 37 (11), CG1 = 38 (10), CG2 = 40 (10)  **immediate post tx**: NR  **1 week:**NR  **Short term**: NR  **intermediate**: NR  **Long term**: NR  **Harms**: NR | |
| **Shankar, 2011[**[**61**](#_ENREF_61)**] India** | **Trial Design:** RCT  **Tx duration**: 3 wks  **Fu duration** (last assessment): immediate post-tx  N screened: NR  N randomized: 60  N completed tx: 60  N attended last fu: 60  **Inclusion**: non-radiating LBP (>=6 mo), age 30-50 yrs, without apparent neurological dficit or any prior history of acu therapy  **Exclusion**: NR | **Mean age (SD/range)**:  Range 30-50, 35.5(5.24) yrs  **% of female**: 63%  **Racial composition**: NR  **Other socio-demographics**: Asian  **Co morbidities**: NR  **Prior episode of pain if acute**: NR  **Prior CAM intervention**: naive acupuncture  **Prior surgery related to current complaint**: NR | **Cause of Pain**:  N-S, Chronic, LBP  **Duration of Pain**:  >6mo; 22.33(13.88) mo  **Severity of pain (Grading)**: NR  **Co-interventions**: NR | Groups  IG (n = 30–E-Acu: UB23, UB24, UB36, UB37, UB40, UB57, UB60, GB30, GB34, GV4;10-20 Hz, 10 points, bilaterally; 20 min, 10 tx delivered on alternate d for 3 wks, De Qi  Drop outs:0  CG (n = 30–usual care: valdecoxib 20mg bid for 10 d; together with supervised physiotherapy for 3 wks  Drop outs: 0 | **Outcomes:**  **Pain**: VAS (10cm),  **Disability**: NR  **Results:** mean (SD)  **Immediate post tx**:  Pain**:** IG = 3.3(1.58), CG = 4.2(1.8)  **Short term:**  NR | **Outcome**  **instruments**:  **QoL/ well being**: NR  **Other**: NR  **Results:**  **Baseline**:  **immediate post tx**: NR  **Short term**: NR  **intermediate**: NR  **Long term**: NR  **Harms**: NR | |
| **Tsui, 2004[**[**62**](#_ENREF_62)**] China** | **Trial Design:** RCT  **Tx duration**: 4 wks  **Fu duration** (last assessment): 3 mo  N screened: NR  N randomized: 42  N completed tx: 42  N attended last fu: 42  **Inclusion**: LBP (>=3 mo), age 20-55 yrs  **Exclusion**: previous hip/back surgery, spinal stenosis with claudication, spine fracture, systemic arthritis, osteoporosis | **Mean age (SD/range)**:  40 yrs  **% of female**: 70%  **Racial composition**: NR  **Other socio-demographics**: Asian  **Co morbidities**: NR  **Prior episode of pain if acute**: NR  **Prior CAM intervention**: NR  **Prior surgery related to current complaint**: NR | **Cause of Pain**:  NR  **Duration of Pain**:  >3mo  **Severity of pain (Grading)**: NR  **Co-interventions**: exercise, analgesics | Groups  IG (n = 14–E-Acu: BL26, GB30;1 Hz, 6 points, bilaterally; 20 min, 2 tx/wk for 4 wks, De Qi  Drop outs: 3  CG (n = 14–exercise: back mob and abdominal stabilization; same as IG.  Drop outs: 1 | **Outcomes:**  **Pain**: VAS (10cm),  **Disability**: RMQ-24  **Results:** mean (SD)  **Immediate post tx**:  Pain**:** IG = 3.07(1.9), CG = 5.5(1.8)  **Short term:**  Pain**:** IG = 2.43(1.87), CG = 5.21 (1.88) | **Outcome**  **instruments**:  **QoL/ well being**: NR  **Other**: NR  **Results:**  **Baseline**:  **immediate post tx**: NR  **Short term**: NR  **intermediate**: NR  **Long term**: NR  **Harms**: NR | |
| **Sator, 2004[**[**63**](#_ENREF_63)**] Austria** | **Trial Design:** RCT  **Tx duration**: 6 wks  **Fu duration** (last assessment): 3 mo  N screened: 87  N randomized: 61  N completed tx: 55  N attended last fu: NR  **Inclusion**: ages >=18 yrs, with LBP for >= 6 mths , pain>5 on VAS 10cm  **Exclusion**: concomitant use of TENS or pacemaker, allergy against lornoxicam or tramadol, history of acu | **Mean age (SD/range)**:  IG=54.1(12.3), CG=53.1(12.1) yrs  **% of female**: 99.3%,  **Racial composition**: NR  **Other socio-demographics**: **NR**  **Co morbidities**: NR  **Prior episode of pain if acute**: NR  **Prior CAM intervention**: naive acu tx  **Prior surgery related to current complaint**: NR | **Cause of Pain**:  Chronic, LBP  **Duration of Pain**:  chronic,4.6(1) yrs  **Severity of pain (Grading)**:  >=5 on VAS 10  **Co-interventions**: NR | **Groups**  **IG** (n =31–electro-acupuncture:1Hz, 10ms, needles 0.2mm×50mm, depth of 20mm, “deqi”, 20min, 1tx/wks for 6wks  **Drop outs**: 2  **CG** (n = 30–usual care: NSAIDs, analgesics, acupuncture, exercises, Other the same as IG.  **Drop outs**: 4 | **Outcomes:**  **Pain**: VAS (10cm),  **Disability**: NR  **Results:** mean (SD)  **Immediate post tx**:  Pain**:** IG = 2(1), CG = 7(1.5)  **Short term:** Pain**:** IG = 1.75(1), CG = 4.75(0.75)  **Intermediate term**: NR | **Outcome**  **instruments**:  **QoL/ well being**: NR  **Other**: NR  **Results:**  **Baseline**:  **immediate post tx**: NR  **Short term**: NR  **Harms**: NR | |
| **Yeung, 2003[**[**64**](#_ENREF_64)**] China** | **Trial Design:** RCT  **Tx duration**: 4 wks  **Fu duration** (last assessment): 3 mo  N screened: NR  N randomized: 52  N completed tx: 52  N attended last fu: 49  **Inclusion**: ages 18-75 years, with LBP for >= 6 mths  **Exclusion**: specific LBP, lumbar surgery, previously acu tx, other ongoing tx within past 3 mo | **Mean age (SD/range)**:  IG=50.4(16.3), CG=55.6(10.4) yrs  **% of female**: IG=84.6%, CG=80.8%  **Racial composition**: Asian  **Other socio-demographics**: **NR**  **Co morbidities**: NR  **Prior episode of pain if acute**: NR  **Prior CAM intervention**: naive acu tx  **Prior surgery related to current complaint**: NR | **Cause of Pain**:  N-S, Chronic, LBP  **Duration of Pain**:  chronic, >=6mths  **Severity of pain (Grading)**:  NR  **Co-interventions**: non- analgesic and NSAID, physical therapy | **Groups**  **IG** (n =26–E-acupuncture: BL23, BL40, SP6. needles 0.3mm×40mm, depth of 20mm, “deqi”, 30min, 2Hz, 3tx/wk for 4 wks  **Drop outs**: C=2  **CG** (n = 26–usual care: NSAIDs, analgesics, exercises 1hr/tx, once a wk for 4 wks, Other the same as IG.  **Drop outs**: C=1 | **Outcomes:**  **Pain**: NRS (10cm),  **Disability**: NR  **Results:** mean (SD)  **Immediate post tx**:  Pain**:** IG = 3.18(2.1), CG = 5.1(2..2)  **Short term:** Pain**:** IG = 3.77(2.12), CG = 5.19(2.47)  **Intermediate term**: NR | **Outcome**  **instruments**:  **QoL/ well being**: NR  **Other**: NR  **Results:**  **Baseline**:  **immediate post tx**: NR  **Short term**: NR  **Harms**: 1 pt got stroke before 3 mo fu | |
| **Meng, 2003[**[**65**](#_ENREF_65)**] USA** | **Trial Design:** RCT  **Tx duration**: 2 wks  **Fu duration** (last assessment): 3 mo  N screened: 250  N randomized: 55  N completed tx: 55  N attended last fu: 55  **Inclusion**: ages >=60 years, with LBP for >= 3 mths  **Exclusion**: specific LBP, lumbar surgery, previously acu tx, other ongoing tx within past 3 mo | **Mean age (SD/range)**:  IG=72(5), CG=70(6) yrs  **% of female**: IG=58%, CG=62.5%  **Racial composition**: 84.7% caucasian  **Other socio-demographics**: **NR**  **Co morbidities**: NR  **Prior episode of pain if acute**: NR  **Prior CAM intervention**: naive acu tx  **Prior surgery related to current complaint**: NR | **Cause of Pain**:  N-S, Chronic, LBP  **Duration of Pain**:  chronic, IG=12(16), CG=12(14) yrs  **Severity of pain (Grading)**:  NR  **Co-interventions**: non- analgesic and NSAID | **Groups**  **IG** (n =31–electro-acupuncture: needles 0.2mm×50mm, depth of 20mm, “deqi”, 20min, 5tx/2wks, 10 tx totally  **Drop outs**: NR  **CG** (n = 24–usual care: NSAIDs, analgesics, exercises, Other the same as IG.  **Drop outs**: NR | **Outcomes:**  **Pain**: VAS (10cm),  **Disability**: NR  **Results:** mean (SD)  **Immediate post tx**:  Pain**:** IG = 1.6(1.1), CG = 2.3(1..2)  **Short term:** Pain**:** IG = 1.4(1.3), CG = 2.4(1.1)  **Intermediate term**: NR | **Outcome**  **instruments**:  **QoL/ well being**: NR  **Other**: NR  **Results:**  **Baseline**:  **immediate post tx**: NR  **Short term**: NR  **Harms**: NR | |
| **Hunter, 2012 [**[**66**](#_ENREF_66)**] Northern Ireland** | **Trial Design:** RCT  **Tx duration**: 12 weks  **Fu duration** (last assessment): 3mons  N screened: 119  N randomized:52  N completed tx: 45  N attended last fu: 44  **Inclusion**: Male/female 18 to 65 y, with chronic (>=3mo) or recurrent (>=3 episodes in previous 12mo) LBP of mechanical origin with/without radiation, Fluency in English (verbal and written).  **Exclusion**:  Currently or having received treatment for CLBP within the previous 3mo. Red ﬂags indicating serious spinal pathology, for example, cancer, cauda equina lesion. Radicular pain indicative of nerve root compression. History of systemic/inﬂammatory disease. Concomitant medical condition that contraindicates acupuncture. Previously received auricular acupuncture. psychological or psychiatric illness. | **Mean age (SD/range)**:  42.8±12.4yrs  **% of female**: 63%  **Racial composition**: NR  **Other socio-demographics**:  Employment, sick leave  **Co morbidities**: NR  **Prior episode of pain if acute**: Episodes of LBP>48 h in past 6mo; IG = 4.33 (1.97)  CG = 4.26 (2.12)  **Prior CAM intervention**: no auricular acupuncture previously  **Prior surgery related to current complaint**: NR | **Cause of Pain**:  Non specific  **Duration of Pain**:  9.9±9 yrs, >= 3mo  **Severity of pain (Grading)**:  mean (95%CI)  VAS (0-10)，  IG= 4.23 (3.17, 5.29); CG=4.93 (3.98, 5.87)  **Co-interventions**: NR | **Groups**  **IG** (n = 27–auricular acupuncture + exercise: o manual AA for the ﬁrst 6 weeks of the trial. at 3 speciﬁc AA points (Shen Men, Lumbar Spine, and Cushion),  **Drop outs**: A = 1, B =4,C=0  **CG** (n = 24–exercise: 12-week intervention program consisting of 6 weeks supervised exercise followed by 6 weeks unsupervised exercise with telephone support, similar to the “Back to Fitness” program  **Drop outs**: A = 0, B =2,C=2 | **Outcomes:**  **Pain**: VAS (0-10cm),  N based on ITT  **Disability**: ODQ (0-100)  N based on ITT  **Results-Baseline:** mean (95%CI)  Pain**:**; IG= 4.23 (3.17, 5.29)(SD 2.68 ); CG=4.93 (3.98, 5.87)(SD 2.24)  Disability:, IG = 25.51 (21.75, 29.27)(SD 9.5); CG = 22.93 (18.22, 27.64)(SD 11.15)  **1 week post tx**: (change from baseline)  Pain**:** CG = -2.12 (-3.23, -1.01)(SD 2.63), IG = -0.93 (-1.98, 0.12)(SD 2.65)  Disability: IG = -6.10 (-9.83, -2.36)(SD 9.44); CG = -7.46 (-11.92, -3.00)(SD 10.56 ),  **Short term**: (change from baseline)  Pain**:** IG = -2.08 (-3.04, -1.13)(SD 2.41); CG = -1.79 (-3.05,-0.53)(SD 2.98),  Disability: IG = -10.67 (-15.36, -5.97)(SD 11.87); CG = -6.67 (-11.44, -1.90)(SD 11.30),  **intermediate**: NR  **Long term**: NR | **Outcome**  **instruments**:  **QoL/ well being**: NR  **Other**: EQ-5D  **Results:**  **Baseline**: mean (95%CI)  IG = 0.68 (0.60, 0.76); CG = 0.67 (0.57, 0.76),  **1 week post tx**: (change from baseline)  IG = 0.05 (-0.02, 0.13); CG = 0.11 (0.04, 0.18),  **Short term**: (change from baseline)  IG = 0.18 (0.12, 0.25); CG = 0.07 (-0.02, 0.16),  **intermediate**: NR  **Long term**: NR  **Harms**: pain (14%), redness (2%), and minor bleeding (1%) at the site of insertion. | |
| **Weiss, 2013 [**[**130**](#_ENREF_130)**] Germany** | **Trial Design:** RCT  **Tx duration**: 21 days  **Fu duration** (last assessment): 3 mons  N screened: NR  N randomized:160  N completed tx: 156  N attended last fu: 143  **Inclusion**: chronic low back pain with duration >= 6 months and age 25–75 years;  **Exclusion**:  contraindications to acupuncture; such as anticoagulation with phenprocoumon or warfarin; coagulation disorders or thrombocytopenia; poor ﬂuency in German language; insufﬁcient adher- ence; recent surgical treatment; and herniated vertebral discs, either minor herniations of less than 6 months’ duration or major herniations of any duration. | **Mean age (SD/range)**:  50.7 yrs, (range, 31–73 years)  **% of female**: 33%  **Racial composition**: NR  **Other socio-demographics**:  Employment, marriage, unable to work  **Co morbidities**: NR  **Prior episode of pain if acute**: NR  **Prior CAM intervention**: NR  **Prior surgery related to current complaint**: NR | **Cause of Pain**:  Non specific  **Duration of Pain**:  11.3 ( 8.4) mons  **Severity of pain (Grading)**:  **Co-interventions**: NR | **Groups**  **IG** (n = 80–acupuncture + rehabilitation: received acupuncture twice weekly, 10 sessions (in 21 days) as one therapeutic course, each session varied between 30 and 40 minutes. standardized 21-day inpatient rehabilitation program according to current German guidelines.  **Drop outs**: A = 1, B =0,C=5  **CG** (n = 80–rehabilitation: a standardized 21-day inpatient rehabilitation program according to current German guidelines.  **Drop outs**: A = 3, B =0,C=8 | **Outcomes:**  **Pain**: VAS (0-100mm),  Not based on ITT  **Disability**: NR  Not based on ITT  **Results-Baseline:** mean (SD)  Pain**:** NR  Disability: NR  **Immediate post tx**:  Pain**:** NR  Disability:NR  **Short term**:  Pain**:**NR  Disability: NR | **Outcome**  **instruments**:  **QoL/ well being**: SF-36  **Other**: NR  **Results:**  **Baseline**: NR  **immediate post tx**: NR  **Short term**: NR  **intermediate**: NR  **Long term**: NR  **Harms**: NR | |
| **Cho, 2013 [**[**38**](#_ENREF_38)**] Republic of Korea** | **Trial Design:** RCT  **Tx duration**: 6 wks  **Fu duration** (last assessment): 6mons  N screened: 142  N randomized:130  N completed tx: 130  N attended last fu: 116  **Inclusion**: cLBP lasting for at least the last 3 months, 10-cm visual analogue scale (VAS) for bothersomeness of LBP exceeding 5, and nonspeciﬁc, uncomplicated LBP that was intact on neurological examination.  **Exclusion**:  sciatic pain, pain mainly below the knee; serious spinal disorders including malignancy, vertebral fracture, spinal infection, inﬂ ammatory spondylitis, and cauda equine compression; history of previous spinal surgery; acupuncture treatment of LBP during the previous month; conditions that could com- promise the safety of acupuncture; severe psychiatric or psychological | **Mean age (SD/range)**:  42.06 ± 14.04 yrs  **% of female**: 84.5%  **Racial composition**: NR  **Other socio-demographics**:  marital status, education, and smoking status  **Co morbidities**: NR  **Prior episode of pain if acute**: NR  **Prior CAM intervention**: NR  **Prior surgery related to current complaint**: NR | **Cause of Pain**:  Non specific  **Duration of Pain**:  >= 3 mons  **Severity of pain (Grading)**:  VAS (0-10)，  IG= 6.52 ± 1.41  CG= 6.37 ± 1.18  **Co-interventions**: None | **Groups**  **IG** (n = 65–Normal acupuncture: 12 sessions (2 times a week for 6 wk), 15 to 20 mins, inserted into the muscle (to a depth of 20mm), true acupoints. operated to get the due sensation (called Diqi)  "Deqi"  **Drop outs**: A = 0, B =7,C=0  **CG** (n = 65–Sham acupuncture: scarried out using the same technique and protocol as real acupuncture, except for the use of a semi- blunt needle on nonacupuncture points without penetration. Eight predeﬁ ned points at the lower back unrelated to traditional acupuncture points were used Other the same as IG.  **Drop outs**: A = 0, B =6,C=1 | **Outcomes:**  **Pain**: VAS (0-10cm),  Not based on ITT  **Disability**: ODI,  Proportion of Outcome Measurements Improvement  Not based on ITT  **Results-Baseline:** mean (SD)  Pain**:** IG= 6.52 ± 1.41; CG= 6.37 ± 1.18  Disability: NR  **Immediate post tx**:  Pain**:** IG = 2.96 ± 2.39, CG = 4.28 ± 1.83  Disability: NR  **2 weeks:**  Pain**:** IG = 3.00 ± 2.41, CG = 4.10 ± 1.85  **Short term:**  Pain**:** IG = 2.78 ± 2.32, CG = 4.06 ± 2.19  Disability:NR  **Intermediate term**:  Pain**:** IG = 2.79 ± 2.44, CG = 3.52 ± 2.53  Disability:NR | **Outcome**  **instruments**:  **QoL/ well being**: SF-36  **Other**: NR  **Results:**  **Baseline**: NR  **immediate post tx**: NR  **Short term**: NR  **intermediate**: NR  **Long term**: NR  **Harms**: Total 10 persons reported: Temporarily worsened LBP (4), Pain at acupunctured site (2), Bruise of acupunctured site (1), Pain, numbness, or other bothersomeness in leg (including knee) (1), Shoulder pain (2). | |
| **Vas, 2012[**[**49**](#_ENREF_49)**] Spain** | **Trial Design:** RCT  **Tx duration**: 2 weks  **Fu duration** (last assessment): 46 wks  N screened: 381  N randomized:275  N completed tx: 261  N attended last fu: 210  **Inclusion**: new episode of N-S acute LBP (<2wks) with or without irradiation; working age; naive acu tx;  **Exclusion**: More than one absence from work because of back pain within a period of 6 months; systemic or specific disease (e.g. tumor, fracture, inflammation);contraindications for acu. | **Mean age (SD/range)**:  18-65 (mean±SD 42.67±11.11) yrs  **% of female**: 58.5%  **Racial composition**: NR  **Other socio-demographics**:  12% college education  **Co morbidities**: NR  **Prior episode of pain if acute**: NR  **Prior CAM intervention**: naiv acu tx  **Prior surgery related to current complaint**: NR | **Cause of Pain**:  Acute, N-S, LBP  **Duration of Pain**:  <0.5 mth (mean±SD 6.06±3.72 days)  **Severity of pain (Grading)**:  (7.04±1.78) on VAS 10cm  **Co-interventions**: Medications (NSAID, analgesics), posture recommendations | **Groups**  **IG** (n = 68–real acu: N-UE-19, SI3, BL62, GV26, SI6, BL60, BL2, TE5, GB41, GB34; needle 0.25mm×25 or 40mm, with a depth of 0.5-1 cun;20min, 5tx/2wks, "Deqi"  **Drop outs**: A = 4, B =17,C=17  **CG1** (n = 68–Sham acu: LU6, LU10, LI12, SP5, PC6, PC5; penetration with a depth of 0.5-1 cun; Other the same as IG.  **Drop outs**: A = 3, B =10,C=15  **CG2** (n = 69–placebo acu: 1 cun from L1-L4 spinous apophysis, blunt needles, non-penetration, at the same points as IG, no deqi; Other the same as IG.  **Drop outs**: A = 5, B =18,C=20  **CG3** (n = 70–conventional tx: avoid remaining in bed, drug prescribed (paracetamol, ibuprofene, diclophenac, ciclobenzaprin). In pts referral form,  **Drop outs**: A = 2, B =10,C=13 | **Outcomes:**  **Pain**: VAS (0-10cm),  **Disability**: RMQ  **Immediate post tx**:  Pain**:** IG = 50pts improved , CG1 = 51 pts improved  Disability: NR  **Short term:**  NR  **Intermediate term**:  NR | **Outcome**  **instruments**:  **QoL/ well being**: NR  **Other**: NR  **Results:**  **Baseline**: NR  **immediate post tx**: NR  **Short term**: NR  **intermediate**: NR  **Long term**: NR  **Harms**: No serious adverse reaction was recorded; With respect to adverse effects provoked by all classes of acupuncture treatment, 8 patients (3.9%) reported increased pain after the treatment session, 3 in the IG group, 3 in the CG1 group, and 2 in the CG2 group. | |
| **Acupressure for NP** | | | | | | | |
| **Yip, 2006 [**[**70**](#_ENREF_70)**]** | **Trial Design:** RCT  **Tx duration**: 3 wks  **Fu duration**  (last assessment): 1mon(s)  N screened: NR  N randomized: 32  N completed tx: 32  N attended last fu: 28  **Inclusion**: (1) (1) aged 18 or above with N-S sub-acute NP for most days in the past 2 weeks; (2) who had not received acupuncture, physiotherapy or manipulative therapy in the last 2 weeks; (3) who could understand the explanation of the study, complete the interview.  **Exclusion**: (1) had NP caused by speciﬁc entities, such as infection, metastases, neoplasm osteoporosis, fractures, radicular cervical syndrome, segmental instability, or spine deformity; (2) pregnant; (3) allergic to oil; (4) had a wound at acupoints at the neck or the shoulder; (5) had a surgical intervention within the last 3 months. | **Mean age**  **(SD/range)**: 51.16 (7.56) yrs  **% of female**: 81%  **Racial composition**: NR  **Other socio-demographics**:  marital status, level of education, occupation  **Co morbidities**: NR  **Prior episode of**  **pain if acute**: NR  **Prior CAM intervention**: NR  **Prior surgery related to current complaint**: None | **Cause of Pain**:  non-speciﬁc  **Duration of Pain**:  Subacute or chronic,＞2 wk(s).  2wks-1mon(s): IG= 11 (78.6%), CG= 12 (66.7%)  **Severity of pain (Grading)**: VAS(0-10), IG= 5.12(2.18); CG= 4.91(1.87)  **Co-interventions**: NR | **Groups**  **IG** (n = 14)–acupoint stimulation + acupressure + conventional treatment: an 8-session (35–40min) over 3 wks for relaxation with electrode pads followed by an acupressure massage with natural aromatic lavender oil (3% lavender oil) and conventional treatment.  **Drop outs**: A =0, B =0,C=3  **CG** (n = 18) –conventional treatment: received conventional treatment alone, which was not recorded, and were offered acupressure treatment after the completion of the study.  **Drop outs**: A =0, B =0,C=1 | **Outcomes:**  **Pain**: VAS (0-10cm)  N based on ITT  **Disability**: NPAD (0-100)  N based on ITT  **Results-Baseline:** Mean (SD)  Pain**:** IG = 5.12(2.18), CG = 4.91(1.87)  Disability: IG = 31.58(13.73), CG = 35.33(13.63)  **1 week**:  Pain: IG = 3.89(2.41), CG = 4.61(2.16)  Disability: IG = 20.30(11.98), CG = 24.48(14.09)  **1 month**:  Pain: IG = 3.26(1.93), CG = 4.29(1.96)  Disability: IG = 14.44(10.70), CG = 25.54(13.89)  **Intermediate**: NR  **Long term**: NR | | **Outcome**  **instruments**:  **QoL/ well being**: NR  **Other**: ROM of lateral spine ﬂexion  **Results:**  **Baseline**: IG= 8.21±5.74; CG= 8.61±6.36  **1 week**: IG= 9.36±6.20; CG= 8.36±5.66  **1 month**: IG= 6.93±3.08; CG= 5.19±3.02  **Intermediate**: NR  **Long term**: NR  **Harms**: no side effect was reported. |
| **Acupressure for LBP** | | | | | | | |
| **Hsieh, 2004 [**[**67**](#_ENREF_67)**] China** | **Trial Design:** RCT  **Tx duration**: 1 mo(s)  **Fu duration**  (last assessment): 6mo(s)  N screened: 250  N randomized: 146  N completed tx: 137  N attended last fu: 121  **Inclusion**: (1) LBP not caused by severe systematic diseases, that is, SLE or rheumatic disease; (2) no contraindications to acupressure and physical therapy, that is, no open wound, an absence of cancer, or psychiatric disease with the presence of overt clinical symptoms before participation; (3) no severe pain, that is, pain score >90%; and (4) no surgical operation prescribed by a physician as treatment for his or her LBP.  **Exclusion**: NR. | **Mean age (SD/range)**:  IG = 47.6 (13.6) vs. CG = 47.6 (14.9)yrs  **% of female**: 52.1%  **Racial composition**: NR  **Other socio-demographics**:  marital status, level of education, occupation  **Co morbidities**: NR  **Prior episode of**  **pain if acute**:NR  **Prior CAM intervention**: NR  **Prior surgery related to current complaint**: None | **Cause of Pain**:  NR  **Duration of Pain**:  pts had a history of  LBP lasting 1mon-10 yrs and 67% pts ＞6 mon(s).  **Severity of pain (Grading)**:  VAS（0-5）  VAS, IG= 1.95 (0–4.0); CG= 2.23 (0.4–5)  **Co-interventions**: NR | **Groups**  **IG** (n = 69)– acupressure; 6 15min(s) sessions,1-2 times/wk, a period of 1 mo(s).  **Drop outs**: A = 0, B = 4,C=0,D=9  **CG** (n = 77) –physical therapy: thermotherapy, infrared light therapy, electrical stimulation, exercise therapy, and pelvic manual traction.. which one decided by one senior physical therapist. followed the routine practice of the hospital.  **Drop outs**: A =0, B =5,C=0,D=8 | **Outcomes:**  **Pain**: SF-PQ (0-45)(Chinese version)  N based on ITT  **Disability**: NR  **Results-Baseline:** mean±SD  Pain: IG = NR  Disability: NR  **Immediate post tx**: Pain: IG = 2.28±2.26, CG = 5.05±5.11  Disability: NR  **Short term**: NR  **Intermediate**:  Pain: IG = 1.08±1,43, CG = 3.15±3.62  Disability: NR  **Long term**: Pain: NR | | **Outcome**  **instruments**:  **QoL/ well being**: NR  **Other**: NR  **Results:**  **Baseline**: NR  **immediate post tx**: NR  **Short term**: NR  **intermediate**: NR  **Long term**: NR  **Harms**: no adverse direct of side effects were reported |
| **Hsieh, 2006 [**[**71**](#_ENREF_71)**] China** | **Trial Design:** RCT  **Tx duration**: 1mo(s)  **Fu duration**  (last assessment): 6mo(s)  N screened: 188  N randomized: 129  N completed tx: 118  N attended last fu: 109  **Inclusion**: 18 years and older with persistent(≥4mo(s)) low back pain.  **Exclusion**:  CLBP was caused by systemic or organic diseases, cancers, or psychiatric diseases; pregnant; acute severe pains needing immediate treatment or surgery; contraindication to acupressure (that is, open wound). | **Mean age**  **(SD/range)**: IG = 50.2 (13.8) vs. CG = 52.6 (17.2) yrs  **% of female**: 70.5%  **Racial composition**: NR  **Other socio-demographics**:  marital status, level of education, occupation  **Co morbidities**: NR  **Prior episode of**  **pain if acute**: Median (range) length of latest pain period  (months), IG= 14.5 (0.02-360); CG= 12 (0.25-432)  **Prior CAM intervention**: NR  **Prior surgery related to current complaint**: None | **Cause of Pain**:  Not caused by systemic or organic diseases, cancers, or psychiatric diseases.  **Duration of Pain**:  chronic, IG＞3 mo(s); CG ＞3 mo(s)  **Severity of pain (Grading)**: CPGQ, IG= 30.6(11.0); CG= 29.6 (11.6)  **Co-interventions**: NR | **Groups**  **IG** (n = 64)– acupressure; 6 15min(s) sessions,1-2 times/wk, a period of 1 mo(s). by one therapist.  **Drop outs**: A = 4, B =0,C=2,D=9  **CG** (n = 65) –physical therapy: thermotherapy, infrared light therapy, electrical stimulation, exercise therapy, and pelvic manual traction.. which one decided by one senior physical therapist. followed the routine practice of the hospital.  **Drop outs**: A =7, B =0,C=6,D=11 | **Outcomes:**  **Pain**: VAS(0-100)  N based on ITT  **Disability**: Roland and Morris disability questionnaire (RMDQ) (0-24)  N based on ITT  **Results-Baseline:** Mean (SD)  Pain: IG = 58.8 (17.88), CG = 57 (17.83)  Disability: IG = 10.9 (6.2), CG = 10.0 (5.3)  **Immediate post tx**:  Pain: IG = 30.6 (21.75), CG = 48.0(23.4)  Disability: IG = 5.4 (5.0), CG = 9.2 (5.8)  **Short term**: NR  **Intermediate**:  Pain: IG = 16.1 (17.4), CG = 41.4  (24.6)  Disability: IG = 2.2 (3.2), CG = 6.7 (5.5)  **Long term**: NR | | **Outcome**  **instruments**:  **QoL/ well being**: NR  **Other**: disability (mODQ)  **Results:**  **Baseline**: IG= 24.4(10.0); CG= 21.1 (8.7)  **immediate post tx**: IG= 17.0(7.6); CG= 20.6 (8.8)  **Short term**: NR  **Intermediate**: IG= 12.2(4.9); CG= 17.9 (8.1)  **Long term**: NR  **Harms**: no adverse direct of side effects were reported |
| **Suen, 2007[**[**69**](#_ENREF_69)**] China** | **Trial Design:** RCT  **Tx duration**: 3 wks  **Fu duration**  (last assessment): 4wks  N screened: 31  N randomized: 21  N completed tx: 21  N attended last fu: 19  **Inclusion**: (1) age >=60 yrs; (2) able to read and write in English; (3) LBP≥3mo(s)  **Exclusion**: LBP caused by specific pathologies, such as infection, metastasis, neoplasm, fractures, spine deformity, or proplapsed disc | **Mean age**  **(SD/range)**: IG = 45.4 (21.8) vs. CG = 49.8 (14.4) yrs  **% of female**: 78.9%  **Racial composition**: n(%)  White: IG= 9 (90%), CG= 8 (89%)  Black: IG= 1 (10%), CG= 1 (11%)  **Other socio-demographics**:  NR  **Co morbidities**: NR  **Prior episode of**  **pain if acute**: NR  **Prior CAM intervention**: NR  **Prior surgery related to current complaint**: None | **Cause of Pain**:  N-S, chronic LBP  **Duration of Pain**:  chronic,＞3 mo(s);  **Severity of pain (Grading)**: NR  **Co-interventions**: NR | **Groups**  **IG** (n = 30)–auricular point acupressure: seven auricular acupoints, shenmen, kidney, urinary bladder, lumbosacral vertebrae and buttock, liver and spleen; use of magnetic pellets with an average of -6.58 mT and diameter of 0.13 cm; pressing, 3wks  **Drop outs**: 10  **CG** (n = 30) –sham:Semen Vaccariae is a small round seed which is of no therapeutic effect if no pressing is applied to it; diameter of 0.13 cm. no pressing, The courses were the same as IG.  **Drop outs**: 9 | **Outcomes:**  **Pain**: VAS 10  N not based on ITT  **Disability**: NR  **Results:** Mean (SD)  **Immediate post tx**:  Pain: IG =1.87 (0.68), CG = 2.27(0.58)  **1 month**:  Pain: IG = 2.2(0.55), CG = 2.27(0.58)  **Intermediate**: NR  **Long term**: NR | | **Outcome**  **instruments**:  **QoL/ well being**: NR  **Harms**: NR |
| **Yeh, 2013 [**[**68**](#_ENREF_68)**] USA** | **Trial Design:** RCT  **Tx duration**: 4 wks  **Fu duration**  (last assessment): 1mo(s)  N screened: 31  N randomized: 21  N completed tx: 21  N attended last fu: 19  **Inclusion**: (1) age 18 years or over; (2) able to read and write in English; (3) LBP≥3mo(s));(4)willing to commit to all the study visits; (5) reported an average CLBP pain intensity score ≥4 on a (VAS,0-10) in the past week.  **Exclusion**:  (1)malignant, autoimmune disease or recent trauma causing their pain; (2) concurrent use of other adjunctive pain therapies (i.e., physical therapy, chiropractic treatment, and acupuncture); (3) previous use of acupressure techniques; (4) allergy to tape; and (5) presence of acute back pain. | **Mean age**  **(SD/range)**: IG = 45.4 (21.8) vs. CG = 49.8 (14.4) yrs  **% of female**: 78.9%  **Racial composition**: n(%)  White: IG= 9 (90%), CG= 8 (89%)  Black: IG= 1 (10%), CG= 1 (11%)  **Other socio-demographics**:  marital status, level of education,  **Co morbidities**: NR  **Prior episode of**  **pain if acute**: NR  **Prior CAM intervention**: NR  **Prior surgery related to current complaint**: None | **Cause of Pain**:  Not caused by systemic or organic diseases, cancers, or psychiatric diseases.  **Duration of Pain**:  chronic, IG＞3 mo(s); CG ＞3 mo(s)  **Severity of pain (Grading)**: RMDQ, IG= 3.48 (0.74); CG= 4.36(1.28)  **Co-interventions**: NR | **Groups**  **IG** (n = 11)–auricular point acupressure; true acupoints with taped seeds on the correctly designated points for CLBP.  three acupoints (i.e., shenmen, sympathetic, and nervous subcortex). first visit lasted from 1.5–2h, and the followup visits were 30mins., a period of 4 wk(s). press the seeds at least 3 times a day for 3 mins at a time. press the seeds for 3 mins whenever they experienced pain. Remove the seeds every 5 days, rest 2 days, replace seeds again.  **Drop outs**: A = 0, B =0,C=1  **CG** (n = 10) –sham: sham acupoints with taped seeds but on diferent acupoints than those designated for CLBP. The courses were the same as IG.  **Drop outs**: A =0, B =0,C=1 | **Outcomes:**  **Pain**: BPI (0-10) (overall pain intensity)  N not based on ITT  **Disability**: Roland and Morris disability questionnaire (RMDQ) (0-24)  N not based on ITT  **Results-Baseline:** Mean (SD)  Pain: IG = 3.48 (0.74), CG = 4.36(1.28)  Disability: IG = 3.30 (2.54), CG = 7.75 (6.23)  **Immediate post tx**:  Pain: IG = 0.86 (0.79), CG = 3.09(1.18)  Disability: IG = 1.67(1.32), CG = 7.00 (6.74)  **1 month**:  Pain: IG = 0.68(0.79), CG = 3.61(1.15)  Disability: IG = 1.90(1.66), CG = 6.13 (5.28)  **Intermediate**: NR  **Long term**: NR | | **Outcome**  **instruments**:  **QoL/ well being**: WHO Quality of Life-BREF (physical) (0-100)  **Other**: disability (mODI)  **Results:**  **Baseline**: IG= 12.63(1.06); CG= 12.64 (1.77)  **immediate post tx**: IG= 12.74(1.75); CG= 13.43 (1.43)  **1 month**: IG= 12.74(1.32); CG= 13.57 (1.49)  **Intermediate**: NR  **Long term**: NR  **Harms**: both groups reported that their ear had more sensitive sensation (N=3,16%), soreness(N=4, 21%), and discomfort (N=4, 21%) at the seed placement. his discomfort usually appeared on day 1∼2 and gradually disappeared. Participants also reported itching (N=7,37%) and sleep disturbance when sleeping on the APA side (N=2, 11%). |
| **Yip, 2004 [**[**72**](#_ENREF_72)**] China** | **Trial Design:** RCT  **Tx duration**: 3 wks  **Fu duration**  (last assessment): 1wk(s)  N screened: NR  N randomized: 61  N completed tx: 51  N attended last fu: 51  **Inclusion**: (1) aged 18 or above with N-S sub-acute LBP for most days in the past 4 weeks; (2) had not received acupuncture, physiotherapy or manipulative therapy in the past week; (3) could understand the explanation of the study, complete the interview, and comprehend the instructions.  **Exclusion**: (1) had LBP caused by speciﬁc entities, such as infection, metastases, neoplasm osteoporosis, fractures, spine deformity, or prolapsed intervertebral disc; (2)had cancer or systemic disorders; (3) pregnant; (4)allergic to lavender aromatic oil; (5)had a wound at the acupoints; (6)had a surgical intervention within the last 3mon(s). | **Mean age**  **(SD/range)**: 45.81 (19.10) yrs  **% of female**: 97%  **Racial composition**: NR  **Other socio-demographics**:  marital status, level of education, occupation  **Co morbidities**: NR  **Prior episode of**  **pain if acute**: NR  **Prior CAM intervention**: NR  **Prior surgery related to current complaint**: None | **Cause of Pain**:  non-speciﬁc  **Duration of Pain**:  Subacute or chronic,＞1 mo(s)  **Severity of pain (Grading)**: VAS, IG= 6.30(0.22); CG= 5.7± 0.37  **Co-interventions**: NR | **Groups**  **IG** (n = 32)–acupoint stimulation + acupressure + conventional treatment: an 8-session over 3 wks for relaxation with electrode pads followed by an acupressure massage with natural aromatic lavender oil (3% lavender oil) and conventional treatment.  **Drop outs**: A =5, B =0,C=0  **CG** (n = 29) –conventional treatment: received conventional treatment alone, which was not recorded, and were offered acupressure treatment after the completion of the study.  **Drop outs**: A =5, B =0,C=0 | **Outcomes:**  **Pain**: VAS (0-10cm)  N not based on ITT  **Disability**: walking time for 15m (50 ft);  N not based on ITT  **Results-Baseline:** Mean (SD)  Pain**:** IG = 6.38 ± 0.22, CG = 5.7 ± 0.37  Disability: IG = 16.61 ± 0.83, CG = 17.47 ± 1.27  **1 week**:  Pain: IG = 0.61 ± 0.06, CG = 0.99 ± 0.06  Disability: IG = 0.91 ± 0.03, CG = 1.03 ± 0.04  **1 month**: NR  **Intermediate**: NR  **Long term**: NR | | **Outcome**  **instruments**:  **QoL/ well being**: NR  **Other**: ROM of lateral spine ﬂexion  **Results:**  **Baseline**: IG= 38.08 ± 1.81; CG= 39.70 ± 1.21  **1 week**: IG= 0.96 ± 0.01; CG= 1.01 ± 0.01  **1 month**: NR  **Intermediate**: NR  **Long term**: NR  **Harms**: no side effect was reported. |
| **Cupping for NP** | | | | | | | |
| **Lauche, 2011 [**[**95**](#_ENREF_95)**] Germany** | **Trial Design:** RCT  **Tx duration**: 2 wks  **Fu duration**  (last assessment):  immediate post-treatment  N screened: 75  N randomized: 50  N completed tx: 45  N attended last fu: 46  **Inclusion**: ages between 18 and 75 and neck pain for at least 5 days a week for at least 3 consecutive months with a mean pain intensity of 40 mm on a 100-mm visual analogue scale (VAS). Non-specific NP.  **Exclusion**:  neck pain caused by trauma or whiplash, inflammatory or malignant disease, congenital malformation of the spine, or neck pain accompanied by radicular symptoms such as radiating pain, paresis, prickling, or tingling. serious acute or chronic organic disease. | **Mean age (SD/range)**:  50.5 ± 11.9yrs  **% of female**: 76%  **Racial composition**: NR  **Other socio-demographics**: NR  **Co morbidities**: NR  **Prior episode of**  **pain if acute**: at least 5 days a week  **Prior CAM intervention**: NR  **Prior surgery related to current complaint**: NR | **Cause of Pain**:  Non-specific  **Duration of Pain**:  >3 mon(s), average of 7.2 ± 6.9 years duration, the majority reported that their pain was permanent and that they had no pain-free intervals (93%).  **Severity of pain (Grading)**:  VAS (0-100)， Sore>40  **Co-interventions**: NR | **Groups**  **IG** (n = 25) – dry-cupping; five cupping treatments 10 to 20 mins, repeated every 3 to 4 days for 2 wks,  **Drop outs**: A = 3, B = 0  **CG** (n = 25) –wait list:  No cupping treatment.  **Drop outs**: A = 1, B =0 | **Outcomes:**  **Pain**: VAS(0-100)  N based on ITT  **Disability**: NDI (0-100)  N based on ITT  **Results-Baseline:** mean (SD)  Pain**:** IG= 45.5 ± 20.9; CG= 42.3 ± 18.0  Disability: IG = 27.5 ± 12.1, CG = 29.1 ± 10.5  **Immediate post tx**:  Pain**:** IG = 26.1 ± 22.7, CG = 47.1 ± 19.8  Disability: IG =21.1 ± 11.2, CG = 29.2 ± 8.4  **Short term**: (baseline adjusted)  NR  **Intermediate**: NR  **Long term**: NR | | **Outcome**  **instruments**:  **QoL/ well being**: SF-36 (General health)  **Other**: PPT  **Results:**  **Baseline**: IG =65.9 ± 21.1, CG = 58.1 ± 18.5  **immediate post tx**:  IG =65.5 ± 23.5, CG = 56.8 ± 16.8  **Short term**: NR  **intermediate**: NR  **Long term**: NR  **Harms**: No side effects were reported. |
| **Lauche, 2012b [**[**74**](#_ENREF_74)**] Germany** | **Trial Design:** RCT  **Tx duration**: 1 time  **Fu duration**  (last assessment):  immediate post-treatment  N screened: 122  N randomized: 50  N completed tx: 45  N attended last fu: 45  **Inclusion**: aged 18 to 75 who suﬀered from neck pain for at least three months in a row with a minimum of 40mm intensity on a 100mm visual analogue scale (VAS). Non-specific NP.  **Exclusion**:  neck pain caused by trauma or whiplash, inflammatory or malignant disease, congenital malformation of the spine, or neck pain accompanied by radicular symptoms such as radiating pain, paresis, prickling, or tingling. serious acute or chronic organic disease. | **Mean age (SD/range)**:  50.5 ± 11.9yrs  **% of female**: 75.6%  **Racial composition**: NR  **Other socio-demographics**: NR  **Co morbidities**: NR  **Prior episode of**  **pain if acute**: NR  **Prior CAM intervention**: NR  **Prior surgery related to current complaint**: NR | **Cause of Pain**:  Non-specific  **Duration of Pain**:  >3 mon(s), average of duration, IG=12 yrs  CG=10.4 yrs  **Severity of pain (Grading)**:  VAS (0-100)， Sore>40  **Co-interventions**: NR | **Groups**  **IG** (n = 25) –Traditional Cupping: traditional cupping treatments 10 to 15 mins, only once.  **Drop outs**: A = 3, B = 0  **CG** (n = 25) –wait list:  No cupping treatment.  **Drop outs**: A = 2, B =0 | **Outcomes:**  **Pain**: VAS(0-100), pain at rest  N based on ITT  **Disability**: NDI (0-100)  N based on ITT  **Results-Baseline:** mean (SD)  Pain**:** IG= 44.9±18.2; CG= 42.6±17.8  Disability: IG = 29.9 ± 11.8, CG = 31.1 ± 9.1  **Immediate post tx**:  Pain**:** IG = 28.5 ± 23.9, CG = 45.7 ± 16.4  Disability: IG =24.5 ± 13.5, CG = 29.0 ± 9.3  **Short term**: (baseline adjusted)  NR  **Intermediate**: NR  **Long term**: NR | | **Outcome**  **instruments**:  **QoL/ well being**: SF-36 (General health)  **Other**: PPT  **Results:**  **Baseline**: IG =62.2 ± 14.2, CG = 64.0 ± 19.3  **immediate post tx**:  IG =64.0 ± 14.8, CG = 61.3 ± 20.7  **Short term**: NR  **intermediate**: NR  **Long term**: NR  **Harms**: One patient reported that the procedure itself was painful, other adverse events including slight reactions such as circulatory instability in the ﬁrst minutes after treatment, tension headaches, a migraine attack, a reappearing tinnitus or wound healing itches. All of these adverse events were minor and transient. |
| **Cramer, 2011 [**[**77**](#_ENREF_77)**] Germany** | **Trial Design:** RCT  **Tx duration**: 2 wks  **Fu duration**  (last assessment):  immediate post-treatment  N screened: 109  N randomized: 50  N completed tx: 48  N attended last fu: 43  **Inclusion**: 18–75 years old and >3 mon(s). The mean pain intensity >4 on (NRS, 0-10), Non-specific NP.  **Exclusion**:  Radicular syndrome, congenital deformity of the spine, spinal stenosis, inflammatory rheumatic disease, active oncologic disease, major depression, insulin-dependent diabetes mellitus, and pregnancy. had invasive treatment of the spine within the previous 4 weeks or spinal surgery within the previous 12 months. Taking oral steroids or anticoagulants or if they had hemophilia or a skin condition in the area to be treated. | **Mean age (SD/range)**:  46.17 ± 12.21yrs  **% of female**: 79.15%  **Racial composition**: NR  **Other socio-demographics**: NR  **Co morbidities**: NR  **Prior episode of**  **pain if acute**: NR  **Prior CAM intervention**: NR  **Prior surgery related to current complaint**: IG=4.2%; CG=0. | **Cause of Pain**:  Non-specific  **Duration of Pain**:  >3 mon(s), average of duration, IG = 107.04 ± 101.84 mon(s)  CG = 107.17 ± 87.18 mon(s)  **Severity of pain (Grading)**:  NRS (0-10)， Sore>4  **Co-interventions**: NR | **Groups**  **IG** (n = 25) –pneumatic pulsation: 5 semi-standardized pneumatic pulsation treatments (cupping + massage) over a period of 2 weeks, 1 treatment every 3–4 days.  **Drop outs**: A = 1, B =3  **CG** (n = 25) –standard medical care: Patients continued self-directed standard medical care (SMC) with their general practitioner or orthopedist. In Germany, SMC for neck pain mainly comprises physiotherapy, sports activities, and analgesics as needed. Patients were allowed to use all such treatments, but no complementary therapies, e.g., acupuncture or homeopathy.  **Drop outs**: A = 1, B =2 | **Outcomes:**  **Pain**: NRS (0-10),  N based on ITT  **Disability**: NDI (0-100)  N based on ITT  **Results-Baseline:** mean (SD)  Pain**:** IG= 4.12 ± 1.45; CG= 4.20 ± 1.57  Disability: IG = 25.92 ± 8.23, CG = 29.17 ± 9.65  **Immediate post tx**:  Pain**:** IG = 2.72 ± 1.62, CG = 4.44 ± 1.96  Disability: IG =20.44 ± 10.17, CG = 28.83 ± 11.94  **Short term**: (baseline adjusted)  NR  **Intermediate**: NR  **Long term**: NR | | **Outcome**  **instruments**:  **QoL/ well being**: SF-36 (PC), SF-36 (MC)  (0-100)  **Other**: NR  **Results:**  **Baseline**:  PC: IG = 43.85 ± 7.65, CG = 41.66 ± 7.09  MC: IG = 46.79 ± 9.97, CG = 47.48 ± 12.21  **immediate post tx**:  PC: IG = 47.60 ± 7.93, CG = 40.49 ± 8.03  MC: IG = 49.83 ± 11.66, CG = 48.07 ± 11.65  **Short term**: NR  **intermediate**: NR  **Long term**: NR  **Harms**: No side effects were reported. |
| **Kim, 2012 [**[**82**](#_ENREF_82)**] Japan** | **Trial Design:** RCT  **Tx duration**: 2 wks  **Fu duration**  (last assessment):  immediate post-treatment  N screened: 49  N randomized: 40  N completed tx: 48  N attended last fu: 43  **Inclusion**: video display terminal users who worked with computers at least 20 hours a week, 18–75 years old, and >3 mon(s). The mean pain intensity >40 on (NRS, 0-100), Non-specific NP.  **Exclusion**:  Serious conditions of the spine and spinal cord (e.g., ankylosing spondylitis), infectious disease, spinal fracture, myelopathy, acute herniation of the cervical intervertebral disk within 3 months, malignancy, immune disorders or whiplash injuries within the previous year. blood-borne diseases or hemostatic abnormalities | **Median age (SD/range)**:  IG=25.5 [22.5 to 40.5]yrs  CG=28 [25 to 41.5] yrs  **% of female**: 55%  **Racial composition**: NR  **Other socio-demographics**: NR  **Co morbidities**: NR  **Prior episode of**  **pain if acute**: NR  **Prior CAM intervention**: NR  **Prior surgery related to current complaint**: NR | **Cause of Pain**:  Non-specific  **Duration of Pain**:  median (range)  IG = 2.5 [1.85 to 4] yrs  CG = 2 [1.25 to 3.75]yr  **Severity of pain (Grading)**:  NRS (0-100)， Sore>40  **Co-interventions**: NR | **Groups**  **IG** (n = 20) –cupping: 3 times per week during a total of 2 wks, 5 to 10 mins, dry or wet cupping.  **Drop outs**: A = 0, B =1  **CG** (n = 20) –heating pad: heating pads warmed by hot water applied to the neck and upper trapezius for 10 mins, 3 times per wk for 2 wks. 55 degrees centigrade. Exercise program details were offered to both treatment groups. Except for this self-exercise program, all other neck pain treatments, such as acupuncture, yoga, Pilates, manipulation therapy, drug therapy, injection therapy, and physical therapy were not allowed.  **Drop outs**: A = 1, B =0 | **Outcomes:**  **Pain**: NRS (0-100),  N based on ITT  **Disability**: NDI (0-100)  N based on ITT  **Results-Baseline:** mean (SD)  Pain**:** IG= 59.25(16.33); CG= 64.85 (14.89)  Disability: IG = 23.33(10.41), CG = 22.96(8.61)  **1 week**:  Pain**:** IG = 28.55(17.83), CG = 64.85(14.89)  Disability: IG = 11.57(8.17), CG = 19.26(10.95)  **1 month**:  Pain**:** IG = 28.75(21.87), CG = 50.3(21.26)  Disability: IG = 10.19(5.99), CG = 20.63(9.82)  **Short term**:  NR  **Intermediate**:  NR  **Long term**: NR | | **Outcome**  **instruments**:  **QoL/ well being**: EQ-5D index  **Other**: NR  **Results:**  **Baseline**: median(range)  IG = 0.91 [0.87, 0.91], CG = 0.89 [0.86,0.91]  **1 week post tx**:  IG = 0.91 [0.91,1.0], CG = 0.91 [0.86, 0.91]  **1 month:**  IG = 1.0 [0.88, 1.0], CG = 0.91 [0.86, 0.91]  **Short term**: NR  **intermediate**: NR  **Long term**: NR  **Harms**: four patients complained skin laceration, whole body itching, pain at the cupping sites, and generalized body ache.  Except for the whole body itching, the other 3 symptoms were assumed to be caused by the cupping therapy. All of the symptoms were mild and disappeared within several days. |
| **Lauche, 2013 [**[**73**](#_ENREF_73)**] Germany** | **Trial Design:** RCT  **Tx duration**: 12 wks  **Fu duration**  (last assessment):  immediate post-treatment  N screened: 246  N randomized: 61  N completed tx: 57  N attended last fu: 57  **Inclusion**: aged 18–75 yrs,  experienced non-specific neck pain > 3 mons, for a minimum of five days a week. mean neck pain intensity > 45 mm (VAS, 0-100mm)  **Exclusion**:  neck pain caused by trauma, disc protrusion, whiplash, congenital deformity of the spine, spinal stenosis, neoplasm, inflammatory rheumatic disease, or active oncologic disease, affective disorder, addiction and psychosis, pregnant, or who had had invasive treatment of the spine within the previous four weeks or spinal surgery within the previous year | **Mean age (SD/range)**:  54.16 (12.7) yrs  **% of female**: 73.8%  **Racial composition**: NR  **Other socio-demographics**:  Marriage, education,  Employment,  **Co morbidities**: NR  **Prior episode of pain if acute**: a minimum of five days a week  **Prior CAM intervention**: massage, pain medication, injection, physiotherapy  **Prior surgery related to current complaint**: NR | **Cause of Pain**:  Non-specific  **Duration of Pain**:  an average of 8 ys of neck pain  **Severity of pain (Grading)**:  VAS (0-100)，  Sore = 56.1(19.0)  **Co-interventions**: NR | **Groups**  **IG** (n = 30) –cupping massage: 2 times per week during a total of 12 wks, 10 to 15 mins, home-based cupping massage.  **Drop outs**: A = 0, B =4  **CG** (n = 31) –Progressive muscle relaxation: practice relaxation at home twice a week for 20 minutes a session, total of 12 wks.  **Drop outs**: A = 0, B =3 | **Outcomes:**  **Pain**: VAS (0-100),  N based on ITT  **Disability**: NDI (0-50)  N based on ITT  **Results-Baseline:** mean (SD)  Pain**:** IG= 55.8(19.7); CG= 56.3 (18.6)  Disability: IG = 15.5(4.3), CG = 17.9(4.9)  **Immediate post tx**:  Pain**:** IG = 39.8(30.0), CG = 45.2(23.5)  Disability: IG = 12.6(5.2), CG = 16.8(5.1)  **Short term**: NR  **Intermediate**: NR  **Long term**: NR | | **Outcome**  **instruments**:  **QoL/ well being**: SF-36 (PC), SF-36 (MC)  (0-100)  **Other**: NR  **Results:**  **Baseline**:  PC: IG = 38.8(8.5), CG = 37.2(6.6)  MC: IG = 47.0(11.5), CG = 47.5(11.6)  **immediate post tx**:  PC: IG = 43.5(10.1), CG = 39.8(8.1)  MC: IG = 45.9(12.8), CG = 46.6(11.6)  **Short term**: NR  **intermediate**: NR  **Long term**: NR  **Harms**: Three people reported side effect. increased muscular tension and pain the morning after cupping massage, but this situation resolved some hours later. Another patient noted increased pain in the shoulder area, but cited a long history of shoulder problems following the use of crutches for a previous knee operation. A third patient was diagnosed with a prolapsed intervertebral disc. |
| **Cupping for LBP** | | | | | | | |
| **Xu, 2009[**[**78**](#_ENREF_78)**] China** | **Trial Design:** RCT  **Tx duration**: 3 wks  **Fu duration**  (last assessment): immediate post-tx  N screened: 105  N randomized:105  N completed tx: 105  N attended last fu: 105  **Inclusion**: (a) lower back pain; (b) current episode of LBP at work or morning.  **Exclusion**:  Cryptomerorachischisis, possible spinal pathology (e.g., carcinoma), disc prolapse, vertebral pedicle crack, The third lumbar transverse process syndrome | **Mean age (SD/range)**:  IG1 = 40.6 (18.9)vs IG2=41.6(19.2) vs. CG =43.2 (16.6)yrs  **% of female**: 36.2%  **Racial composition**: Asian  **Other socio-demographics**: **NR**  **Co morbidities**: NR  **Prior episode of**  **pain if acute**: NR  **Prior CAM intervention**: NR  **Prior surgery related to current complaint**: NR | **Cause of Pain**:  Back strain  **Duration of Pain**:  IG1 = 46.4 (13.9)vs IG2=43.4(11.9) vs. CG =44.8 (11.4 )mo  **Severity of pain (Grading)**: NR  **Co-interventions**: NR | **Groups**  **IG 1**(n =35) –Balance-cupping; Bilateral low back area, along BL and GV, every 2 days  **Drop outs**: A = 0  **IG2** (n = 35) –Cupping with retention: Bilateral at BL, 15 min daily.  **Drop outs**: A = 0  **CG** (n = 35) –Diclofenac: 50 mg, daily, orally  **Drop outs**: A = 0 | **Outcomes:**  **Pain**: VAS(100mm)  N not based on ITT  **Disability**: ODI (0-60)  N not based on ITT  **Results-Baseline:** mean (SD)  Pain**:** IG1 = 59.98 (12.91), IG2=58.76(13.69); CG = 57.32 (14.31)  Disability: IG1 = 30.66 (8.49), IG2=28.41(9.46); CG = 30.03 (9.21)  **Immediate post tx**: Pain**:** IG1 = 10.21(5.81), IG2 = 17.32(6.95); CG = 16.57 (6.31)  Disability: IG1 = 10.21 (3.69), IG2=14.72(5.43); CG = 16.93 (4.39)  **Short term**: NR | | **Outcome**  **instruments**:  **QoL/ well being**: NR  **Results:**  **Baseline**: NR  **immediate post tx**: NR  **Short term**: NR  **intermediate**: NR  **Long term**: NR  **Harms**: NR |
| **Li, 2009[**[**79**](#_ENREF_79)**] China** | **Trial Design:** RCT  **Tx duration**: 3 wks  **Fu duration**  (last assessment): immediate post-tx  N screened: unclear  N randomized:90  N completed tx: 90  N attended last fu: 90  **Inclusion**: (a) lower back pain; (b) current episode of LBP; (c) no systemic disease; (d) age 30-55 yrs  **Exclusion**:  spinal pathology (e.g., carcinoma), fracture, immune disease, nerve deficit, disc prolapse, vertebral pedicle crack, | **Mean age (SD/range)**:  IG1 = 30.5(8.9)vs IG2=31.7(9.7) vs. CG =30.9 (9.3)yrs  **% of female**: NR  **Racial composition**: Asian  **Other socio-demographics**: **NR**  **Co morbidities**: NR  **Prior episode of**  **pain if acute**: NR  **Prior CAM intervention**: NR  **Prior surgery related to current complaint**: NR | **Cause of Pain**:  N-S, LBP  **Duration of Pain**:  IG1 = 12.9 (3.1)vs IG2=11.6(2.9) vs. CG =13.1 (3.5 )mo  **Severity of pain (Grading)**: NR  **Co-interventions**: NR | **Groups**  **IG 1**(n =30) –wet cupping; Bilateral low back area, ashi points, blood letting 10-20ml, cupping, once a week  **Drop outs**: A = 0  **IG2** (n = 30) –Cupping with retention: Bilateral at BL, 15 min daily interval  **Drop outs**: A = 0  **CG** (n = 30) –Diclofenac: 50 mg, daily, orally  **Drop outs**: A = 0 | **Outcomes:**  **Pain**: VAS(10cm)  N not based on ITT  **Disability**: ODI (0-60)  N not based on ITT  **Results-Baseline:** mean (SD)  Pain**:** IG1 = 5.8 (1.9), IG2=5.6(2.0); CG = 5.9 (2.1)  Disability: IG1 = 31.6 (6.9), IG2=30.2(6.3); CG = 29.8 (6.1)  **Immediate post tx**: Pain**:** IG1 = 1.2(0.8), IG2 = 2.1(1.1); CG = 2.3 (1.4)  Disability: IG1 = 9.8 (2.7), IG2=14.6(3.2); CG = 15.7 (3.8)  **Short term**: NR | | **Outcome**  **instruments**:  **QoL/ well being**: NR  **Results:**  **Baseline**: NR  **immediate post tx**: NR  **Short term**: NR  **intermediate**: NR  **Long term**: NR  **Harms**: NR |
| **Liu, 2008[**[**80**](#_ENREF_80)**] China** | **Trial Design:** RCT  **Tx duration**: 3 wks  **Fu duration**  (last assessment): immediate post-tx  N screened: 75  N randomized:75  N completed tx: 75  N attended last fu: 75  **Inclusion**: (a) lower back pain; (b) current episode of LBP at work or morning; (c) no systemic disease; (d) no psychological illness; (e) age 35-65 yrs  **Exclusion**:  possible spinal pathology (e.g., carcinoma, fracture, osteoporosis), disc prolapse, self immune disease, nerve dificits | **Mean age (SD/range)**:  IG1 = 39.1(17.1)vs IG2=38.6(18.5) vs. CG =35.2 (14.3)yrs  **% of female**: 45.3%  **Racial composition**: Asian  **Other socio-demographics**: **NR**  **Co morbidities**: NR  **Prior episode of**  **pain if acute**: NR  **Prior CAM intervention**: NR  **Prior surgery related to current complaint**: NR | **Cause of Pain**:  N-S, LBP  **Duration of Pain**:  IG1 = 28.7(9.9)vs IG2=26.9(7.8) vs. CG =24.9(9.3 )mo  **Severity of pain (Grading)**: NR  **Co-interventions**: NR | **Groups**  **IG 1**(n =25) –Balance-cupping; Bilateral low back area, along BL and GV, every 2 days  **Drop outs**: A = 0  **IG2** (n = 25) –Cupping with retention: Bilateral at BL, 15 min daily.  **Drop outs**: A = 0  **CG** (n = 25) –Diclofenac: 50 mg, daily, orally  **Drop outs**: A = 0 | **Outcomes:**  **Pain**: VAS(10cm)  N not based on ITT  **Disability**: ODI (0-60)  N not based on ITT  **Results-Baseline:** mean (SD)  Pain**:** IG1 = 6.23 (1.64), IG2=5.97(1.75); CG = 5.86 (1.64)  Disability: IG1 = 30.53 (8.43), IG2=28.69(9.57); CG = 29.72 (9.43)  **Immediate post tx**: Pain**:** IG1 = 1.23(0.32), IG2 = 1.79(0.53); CG = 1.88 (0.41)  Disability: IG1 = 10.53 (3.43), IG2=14.69(5.57); CG = 15.72 (4.43)  **Short term**: NR | | **Outcome**  **instruments**:  **QoL/ well being**: NR  **Results:**  **Baseline**: NR  **immediate post tx**: NR  **Short term**: NR  **intermediate**: NR  **Long term**: NR  **Harms**: NR |
| **Hong, 2006[**[**81**](#_ENREF_81)**] China** | **Trial Design:** RCT  **Tx duration**: 11 d  **Fu duration**  (last assessment): immediate post-tx  N screened: unclear  N randomized:70  N completed tx: 70  N attended last fu: 70  **Inclusion**: (a) lower back pain; (b) current episode of LBP; (c) no systemic disease; (d) no psychological illness; (e) age 23-67 yrs  **Exclusion**:  possible spinal pathology (e.g., carcinoma, fracture, osteoporosis), infection, disc prolapse, self immune disease, nerve dificits | **Mean age (SD/range)**:  37.44 (10.65)yrs totally  **% of female**: 68.6%  **Racial composition**: Asian  **Other socio-demographics**: **NR**  **Co morbidities**: NR  **Prior episode of**  **pain if acute**: NR  **Prior CAM intervention**: NR  **Prior surgery related to current complaint**: NR | **Cause of Pain**:  N-S, LBP  **Duration of Pain**:  12.90(12.42 )mo  **Severity of pain (Grading)**: NR  **Co-interventions**: NR | **Groups**  **IG 1**(n =25) –Moving-cupping; Bilateral at BL, 5–10 times (about 5 min), alternate days for 11 days  **Drop outs**: A = 0  **CG** (n = 25) –Dexibuprofen: 0.15 g, T.I.D. for 12 days, orally  **Drop outs**: A = 0 | **Outcomes:**  **Pain**: VAS(10cm)  N not based on ITT  **Disability**: NR  **Results-Baseline:** mean (SD)  Pain**:** IG = 6.11(2.08), CG = 5.86 (1.99)  **Immediate post tx**: Pain**:** IG = 1.29(1.62), CG = 3.57 (2.96)  **Short term**: NR | | **Outcome**  **instruments**:  **QoL/ well being**: SF-36  **Results:**  **Baseline**: NR  **immediate post tx**: NR  **Short term**: NR  **intermediate**: NR  **Long term**: NR  **Harms**: NR |
| **Farhadi, 2009 [**[**83**](#_ENREF_83)**] Iran** | **Trial Design:** RCT  **Tx duration**: 6 days  **Fu duration**  (last assessment): 3mo(s)  N screened: 106  N randomized: 98  N completed tx: 98  N attended last fu: 86  **Inclusion**: (a) lower back pain persisting for 4 wks or more; (b) age 17—68 yrs; and (c) current episode of low back pain having at least a 4-week duration.  **Exclusion**:  possible spinal pathology (e.g., carcinoma), severe or progressive motor weakness or central disc prolapse, pending litigation (e.g., workplace injury), bleeding disorders (e.g., hemophilia), and current treatment with wet-cupping. | **Mean age (SD/range)**:  IG = 44.9 (14.8) vs. CG =41.8 (13.9)yrs  **% of female**: 30.6%  **Racial composition**: Asian  **Other socio-demographics**:  Previous back surgy  **Co morbidities**: NR  **Prior episode of**  **pain if acute**: NR  **Prior CAM intervention**: NR  **Prior surgery related to current complaint**: IG=8.3%, CG=10%. | **Cause of Pain**:  Non-specific  **Duration of Pain**:  IG=52.7 (71.7) mon(s); CG=55 (49.7) mon(s)  **Severity of pain (Grading)**: PPI (0-6)  IG= 2.7 (0.8); CG= 2.7 (0.9)  **Co-interventions**: NR | **Groups**  **IG** (n = 48) – wet-cupping; 10-12 a series of three staged wet-cupping treatments, placed at 3 days intervals (i.e., 0, 3, and 6 days). lasted about 20min and was conducted in ﬁve steps  **Drop outs**: A = 0, B = 0,C=7,  **CG** (n = 50) –usual care:  This treatment included: (1) encouragement for early return to usual activities, excluding heavy manual labor, (2) activity alteration to minimize symptoms, (3) acetaminophen, or NSAIDs, (4) short duration muscular relaxants or opioids (optional, based on patient preference), (5) bed rest—–not more than 2 days (optional, based on patient preference), and (6), spinal manipulation exercises..  **Drop outs**: A = 0, B =0,C=5 | **Outcomes:**  **Pain**: PPI(0-5)  N not based on ITT  **Disability**: ODI (0-60)  N not based on ITT  **Results-Baseline:** mean (SD)  Pain**:** IG = 2.7 (0.8) CG = 2.7 (0.9)  Disability: IG = 31.4 (6.6), CG = 30.9 (9.8)  **Immediate post tx**: NR  **Short term**:  Pain: IG = 0.7 (0.9), CG = 2.8 (1.3)  Disability: IG = 15.6 (6.7), CG = 30.6 (11.6)  **Intermediate**: NR  **Long term**: NR | | **Outcome**  **instruments**:  **QoL/ well being**: NR  **Other**: MQS  **Results:**  **Baseline**: NR  **immediate post tx**: NR  **Short term**: NR  **intermediate**: NR  **Long term**: NR  **Harms**: fainting (vaso-vagal shock) (3 patients) |
| **Kim, 2011 [**[**76**](#_ENREF_76)**] Republic of Korea** | **Trial Design:** RCT  **Tx duration**: 4 wks  **Fu duration**  (last assessment):  immediate post-treatment  N screened: 62  N randomized: 32  N completed tx: 32  N attended last fu: 29  **Inclusion**: continued low back pain for at least 12 weeks without recognisable specific causes such as radicular syndrome, infection or tumour.  **Exclusion**:  not meet the definition of PNSLBP or not suitable for wet-cupping treatment due to medical conditions (e.g., haematologic disease, anticoagulant use or systemic disease, such as diabetes and cardiovascular or renal disease), undergone cupping or alternative therapies during the previous 3 mon(s) and any therapies for PNSLBP during the previous 2 wks. | **Mean age (SD/range)**:  IG = 44.2 (9.4) vs. CG =48.1 (5.4)yrs  **% of female**: 75%  **Racial composition**: Asian  **Other socio-demographics**: NR  **Co morbidities**: NR  **Prior episode of**  **pain if acute**: NR  **Prior CAM intervention**: NR  **Prior surgery related to current complaint**: NR | **Cause of Pain**:  Non-specific  **Duration of Pain**:  >3 mon(s)  **Severity of pain (Grading)**:  NRS (0-100)  IG= 58.10(11.23); CG= 52.73(8.00)  **Co-interventions**: NR | **Groups**  **IG** (n = 21) – wet-cupping;  3 times/wk for 2 wks, treatment points were located bilaterally at BL23, BL24, and BL25. Offered a brochure about exercise, general advice for PNSLBP, and 500 mg acetaminophen tablets to both groups. Lumbar supports and hot packs could also be used. However, other treatments were forbidden..  **Drop outs**: A = 0, B = 0,C=2  **CG** (n = 11) –wait list:  No cupping treatment. Other treatments were the same as IG.  **Drop outs**: A = 0, B =0,C=1 | **Outcomes:**  **Pain**: NRS (0-100)  N based on ITT  **Disability**: ODQ (0-50)  N based on ITT  **Results-Baseline:** mean (SD)  (baseline adjusted)  Pain**:** (NRS) IG= 58.10(11.23); CG= 52.73(8.00)  Disability: IG = 47.94 (11.56), CG = 48.00 (10.88)  **Immediate post tx**: (baseline adjusted)  Pain**:** (NRS) IG = -16.0(19.5), CG = -9.1(15.2)  Disability: IG = -5.6(7.7), CG = -1.8(6.77)  **2 weeks**: (baseline adjusted)  Pain**:** (NRS) IG = -18.2(18.2), CG = 17.4(14.0)  Disability: IG = -7.3(8.42), CG = -4.9(8.29)  **Intermediate**: NR  **Long term**: NR | | **Outcome**  **instruments**:  **QoL/ well being**: NR  **Other**: PPI(0-5)  **Results:**  **Baseline**:Pain: (PPI) IG = 2.43 (0.75), CG = 1.91 (0.70)  **immediate post tx**: (baseline adjusted)  Pain**:** (PPI) IG =1.2(0.94), CG = -0.2(1.0)  **2 weeks:** (baseline adjusted)  Pain**:** (PPI) IG = -1.3(1.05), CG =-0.4(0.59)  **Short term**: NR  **intermediate**: NR  **Long term**: NR  **Harms**: fainting (vaso-vagal shock) (3 patients) |
| **Gua sha for CNP** | | | | | | | |
| **Braun, 2011 [**[**85**](#_ENREF_85)**] Germany** | **Trial Design:** RCT  **Tx duration**: 30min(s)  **Fu duration**  (last assessment): 7d posttreatment  N screened: 101  N randomized: 48  N completed tx: 48  N attended last fu: 44  **Inclusion**: Men and women, 18-70 yrs with a self-reported painful restriction of cervical spine mobility, long-term (＞3 mo(s)) NP, with ≥ 30 mm on VAS.  **Exclusion**:  had undergone invasive treatment within the previous month, receiving anti-coagulants or had hemophilia, anemia, skin disease in the region of treatment, or a coexisting serious illness. participating in another study, experienced treatments with Gua sha or the ginger heat pad, undergone previous surgery in the neck region or had a manifest neurological deﬁcit. | **Mean age (SD/range)**:  58.5 ±8.0 yrs  **% of female**: 85.5%  **Racial composition**: NR  **Other socio-demographics**: NR  **Co morbidities**: NR  **Prior episode of**  **pain if acute**: NR  **Prior CAM intervention**: NR  **Prior surgery related to current complaint**: NR | **Cause of Pain**:  NR  **Duration of Pain**:  Mean duration of illness was about 8 yrs in both groups.  **Severity of pain (Grading)**:  for average NP, week before the baseline measure, ≥ 30 mm on VAS.  **Co-interventions**: NR | **Groups**  **IG** (n = 24)–Gua sha; 30min(s), totally once, using a small lid with a rounded edge and a skin lubricant  **Drop outs**: A = 0, B = 3  **CG** (n = 24) –thermal therapy: once for 15–20 minutes, heat pad with external ginger(Chinese medicine)  **Drop outs**: A = 0, B =1 | **Outcomes:**  **Pain**: VAS(0-100)  N based on ITT  **Disability**: NDI (0-100)  N based on ITT  **Results-Baseline:** mean±SD  Pain**:** IG = 61.3±14.0 CG = 58.3±16.2  Disability: IG = 32.8±11.5, CG = 35.6±11.0  **Immediate post tx**: 7 d average  Pain: IG = 22.2±22.3, CG = 50.3±23.4  Disability: IG = 21.8±12.9, CG = 32.8±12.5  **Short term**: NR  **Intermediate**: NR  **Long term**: NR | | **Outcome**  **instruments**:  **QoL/wellbeing**: SF-36(0 to 100)mental component, physical component  **Other**: pain related to motion  **Results:** mean±SD  **Baseline**: [SF-mental]IG= 42.8±12.7; CG-= 41.6±12.0  [SF-physical] IG= 41.8±7.9; CG-= 41.2±9.8  **immediate post tx**: 7 days, both improved significantly while IG compared with CG.  **Short term**: NR  **intermediate**: NR  **Long term**: NR  **Harms**: petechiae, slight muscle aches and soreness in application area, and so on. all are not serious. |
| **Lauche, 2012a [**[**84**](#_ENREF_84)**] Germany** | **Trial Design:** RCT  **Tx duration**: 10–15min(s)  **Fu duration**  (last assessment): 7d posttreatment  N screened: NR  N randomized: 21  N completed tx: 20  N attended last fu: 20  **Inclusion**: Men and women, 18-75 yrs with N-S CNP at least ﬁve days a week, long-term (＞3 mo(s)), with ≥ 4cm on VAS(0-10cm), previously excluded speciﬁc causes for pain.  **Exclusion**:  vertebral disc prolapse, trauma, inﬂammatory or malignant disease, and congenital malformation of the spine, as well as radicular. anticoagulation treatment or a tendency to hemorrhage. mental health disorders,  pregnancy, had invasive treatments within the previous month, spinal surgery within the last year or previous treatment with corticosteroids or opiates. | **Mean age (SD/range)**:  58.5 ±8.0 yrs  **% of female**: 81%  **Racial composition**: NR  **Other socio-demographics**: NR  **Co morbidities**: NR  **Prior episode of**  **pain if acute**: NR  **Prior CAM intervention**: NR  **Prior surgery related to current complaint**: NR | **Cause of Pain**:  NR  **Duration of Pain**:  duration of pain was ＞3 mo(s) in both groups.  **Severity of pain (Grading)**:  for average NP, week before the baseline measure, ≥ 4cm on VAS(0-10cm).  **Co-interventions**: NR | **Groups**  **IG**(n=10)–Gua sha; 10–15min(s), totally once, using a small lid with a rounded edge and a skin lubricant, applied from C7 to T12  **Drop outs**: A = 0, B = 0  **CG** (n = 11) –wait list:  no treatment  **Drop outs**: A = 0, B =1 | **Outcomes:**  **Pain**: VAS(0-10)  N based on ITT  **Disability**: NR  **Results-Baseline:** mean±SD  Pain**:** IG = 4.3±1.7 CG = 5.2±1.6  Disability: NR  **Immediate post tx**: 7 d average  Pain: IG = 3.0±2.2, CG = 5.1±1.4  Disability: NR  **Short term**: NR  **Intermediate**: NR  **Long term**: NR | | **Outcome**  **instruments**:  **QoL/wellbeing**: SF-36 (GH)  **Other**: Pressure Pain Thresholds(PPT)  **Results:** mean±SD  **Baseline**: NR  **immediate post tx**: 7 days, both GH and PPT improved significantly while IG compared with CG.  **Short term**: NR  **intermediate**: NR  **Long term**: NR  **Harms**: No adverse events were reported. |
| **Gua sha for CLBP** | | | | | | | |
| **Lauche, 2012b [**[**84**](#_ENREF_84)**] Germany** | **Trial Design:** RCT  **Tx duration**: 10–15min(s)  **Fu duration**  (last assessment): 7d posttreatment  N screened: NR  N randomized: 19  N completed tx: 18  N attended last fu: 18  **Inclusion**: Men and women, 18-75 yrs with N-S CLBP at least ﬁve days a week, long-term (＞3 mo(s)), with ≥ 4cm on VAS(0-10cm), previously excluded speciﬁc causes for pain.  **Exclusion**:  vertebral disc prolapse, trauma, inﬂammatory or malignant disease, and congenital malformation of the spine, as well as radicular. anticoagulation treatment or a tendency to hemorrhage. mental health disorders, pregnancy, had invasive treatments within the previous month, spinal surgery within the last year or previous treatment with corticosteroids or opiate | **Mean age (SD/range)**:  58.5 ±8.0 yrs  **% of female**: 72.2%  **Racial composition**: NR  **Other socio-demographics**: NR  **Co morbidities**: NR  **Prior episode of**  **pain if acute**: NR  **Prior CAM intervention**: NR  **Prior surgery related to current complaint**: NR | **Cause of Pain**:  NR  **Duration of Pain**:  Mean duration of illness was about 8 yrs in both groups.  **Severity of pain (Grading)**:  for average LBP, week before the baseline measure, ≥ 30 mm on VAS.  **Co-interventions**: NR | **Groups**  **IG** (n = 10)–Gua sha; 10–15min(s), totally once, using a small lid with a rounded edge and a skin lubricant, applied from C7 to L5.  **Drop outs**: A = 0, B = 0  **CG** (n = 9) –wait list: no treatment  **Drop outs**: A = 0, B =1 | **Outcomes:**  **Pain**: VAS(0-10)  N based on ITT  **Disability**: NR  **Results-Baseline:** mean±SD  Pain**:** IG = 3.4±2.4 CG = 3.3±2.1  Disability: NR  **Immediate post tx**: 7 d average  Pain: IG = 2.1±1.9, CG = 3.1±2.4  Disability: NR  **Short term**: NR  **Intermediate**: NR  **Long term**: NR | | **Outcome**  **instruments**:  **QoL/wellbeing**: SF-36(GH)  **Other**: Pressure Pain Thresholds(PPT)  **Results:** mean±SD  **Baseline**: NR  **immediate post tx**: 7 days, GH improved significantly while IG compared with CG. However, not for PPT.  **Short term**: NR  **intermediate**: NR  **Long term**: NR  **Harms**: No adverse events were reported. |
| **Qigong for CNP** |  |  |  |  |  |  | |
| **Lansinger, 2007 [**[**88**](#_ENREF_88)**] Swedish** | **Trial Design:** RCT  **Tx duration**: 3 mo(s)  **Fu duration**  (last assessment): 12mo(s)  N screened: NR  N randomized: 139  N completed tx: 102  N attended last fu: 100  **Inclusion**: Men and women, 18-65 yrs with non-speciﬁc, long-term (＞3 mo(s)) NP, with ≥ 20 mm on VAS.  **Exclusion**:  chronic tension-type head- ache, migraine, traumatic neck injuries, neurologic signs or symptoms, rheumatic diseases, ﬁbromyalgia or other severe physiologic or physical diseases, treatment with anti-depressive and anti-inﬂammatory drugs, and difﬁculties in understanding the Swedish language. | **Mean age (SD/range)**:  IG = 44.9 (12.3) vs. CG = 42.8 (1.4)yrs  **% of female**: 70%  **Racial composition**: NR  **Other socio-demographics**:  Sick leave(100%)=5pts (4%), IG=2 (3%), CG=3 (5%)  **Co morbidities**: NR  **Prior episode of**  **pain if acute**: IG = 64Pt(s); CG = 59Pt(s)  **Prior CAM intervention**: IG= 67 Pt(s); CG= 64 Pt(s)  **Prior surgery related to current complaint**: None | **Cause of Pain**:  NR  **Duration of Pain**:  46 (38%) pts had a history of  NP lasting 1- 5 yrs and 55 (45%) pts ＞5 yrs.  **Severity of pain (Grading)**:  median (range) for average NP, week before the baseline measure, 53 mm (20–100 mm).VAS.  VAS, IG= 50 (20–100); CG= 56 (20–97)  **Co-interventions**: NR | **Groups**  **IG** (n = 72)– qigong(Biyun method); 10-12 1hr sessions,1-2 times/wk, a period of 3 mo(s). Biyun  **Drop outs**: A = 12, B = 12,D=1,E=0  **CG** (n = 67) –exercise: 10-12 1hr sessions,1-2 times/wk, a period of 3 mo(s).  **Drop outs**: A = 5, B = 8,D=0,E=0 | **Outcomes:**  **Pain**: VAS(0-100)  N based on ITT  **Disability**: NDI (0-70)  N based on ITT  **Results-Baseline:** median (range)**Pain:** IG = 50 (20–100) CG = 56 (20–97)  **Disability**: IG = 26 (6–60), CG = 22 (8–52)  **Immediate post tx**:  Pain: IG = 41 (2–81),  CG = 26 (0–84)  Disability: IG = 24 (2–68), CG = 17 (2–52)  **Short term**: NR  **Intermediate**:  Pain: IG = 34 (0–95), CG = 27 (0–85)  Disability: IG = 22 (0–64), CG = 18 (0–56)  **Long term**: Pain: IG = 35 (0–87), CG = 30 (0–91)  Disability: IG = 22 (0–54), CG = 18 (0–52) | **Outcome**  **instruments**:  **QoL/ well being**: NR  **Other**: Cervical ROM rotation; Cervical ROM ﬂexion-extension  **Results:** median (range)  **Baseline**: IG= 123 (50–170),CG= 123 (63–190); IG= 110 (60–153); CG= 110 (50–160)  **immediate post tx**: IG= 140 (50–200),CG= 140 (63–180);  IG= 120 (46–198),CG= 118 (73–175)  **Short term**: NR  **intermediate**: IG= 140 (50–200),CG= 140 (63–180);  IG= 120 (60–198),CG= 115 (73–160)  **Long term**: IG=140 (50–180),CG= 150 (63–180);  IG=120 (60–170); CG= 119 (73–165)  **Harms**: NR | |
| **Von trot, 2009 [**[**87**](#_ENREF_87)**] Germany** | **Trial Design:** RCT  **Tx duration**: 3 mo(s)  **Fu duration**  (last assessment): 6mo(s)  N screened: 328  N randomized: 121  N completed tx: 117  N attended last fu: 93  **Inclusion**: age≥55 yrs, had recurrent neck pain≥6 mon（s）, average pain intensity ≥20 mm（VAS）in the 7 d  **Exclusion**:  serious acute or chronic organic illness or mental disorder that disallowed participation in the study, planned start of a physiotherapeutic treatment for neck pain during study participation, or participation in another study during the last 6 months before study entry. | **Mean age (SD/range)**:  mean age 76.0(8) yrs  IG = 75.9(7.6) vs. CG-ex = 76.0(7.2) vs. CG-wt= 75.7( 7.6)yrs  **% of female**: 95%  **Racial composition**: NR  **Other socio-demographics**:  Living alone, IG= 60.5%; CG-ex= 66.7%;CG-wt= 65.0%  **Co morbidities**:  cardiovascular diseases playing the leading role (97 of 117; 83%).  **Prior episode of**  **pain if acute**: NR  **Prior CAM intervention**: last 3 months before study entry, 43 patients (37%) had consulted at least 1 physiotherapist for their neck pain  **Prior surgery related to current complaint**: NR | **Cause of Pain**:  NR  **Duration of Pain**:  19.0±14.9 years  **Severity of pain (Grading)**:  mean±SD for average NP, week before the baseline measure, 53 mm (20–100 mm).VAS.  VAS, IG= 56.4(19.7); CG-ex= 47.1(19.6);CG-wt= 49.9 (20.3)  **Co-interventions**: NR | **Groups**  **IG** (n = 38)– qigong(Dantian style): 24 sessions (each 45 minutes), period of 3mo（s）,2 sessions/wk, in groups of 6 to 12 pts, and used the same gymnasiums.  **Drop outs**: A =?, B =?,C=7,D=4  **CG** (n = 39) –exercise: 24 sessions (each 45 minutes), period of 3mo（s）,2 sessions/wk, in groups of 6 to 12 pts, and used the same gymnasiums.  **Drop outs**: A =?, B =?,C=4,D=1  CG(n=40)—waitlist: did not receive qigong or exercise therapy for the whole period  **Drop outs**: A =?, B =?,C=5,D=3  Note: In all groups, Pts free to treat their neck pain with the treatment or therapies they were using prior to randomization. | **Outcomes:**  **Pain**: VAS(0-100)  N based on ITT  **Disability**: NPAD (0-100)  N based on ITT  **Results-Baseline:** mean±SD  Pain**:**IG=56.4(19.7); CG-ex=47.1(19.6);CG-wt= 49.9 (20.3)  Disability: IG= 38.5（19.2）; CG-ex= 41.8（24.9）;CG-wt= 36.1（20.8）  **Immediate post tx**: NR  **Short term**: Pain**:** IG= 47.4(30.8); CG-ex= 44.5(25.7);CG-wt=54.9(28.5)  Disability: IG= 34.3(23.6); CG-ex= 33.6 (25.5);CG-wt= 39.1(21.7)  **Intermediate**:  Pain**:** IG=53.1(30.6); CG-ex= 47.7(30.5);CG-wt= 59.9(25.5)  Disability: IG= 39.8(25.8); CG-ex= 34.3(24.8);CG-wt= 41.3(23.4)  **Long term**: NR | **Outcome**  **instruments**:  **QoL/ well being**:  SF-36(0 to 100)mental component, physical component  **Other**: ADS(0-60) depression  **Results:** mean±SD  **Baseline**: [SF-mental]IG= 30.4(7.9); CG-ex= 49.6(10.9);CG-wt= 49.9(9.1)  [SF-physical] IG= 30.4(7.9); CG-ex= 28.7(7.2);CG-wt= 30.6 (9.3)  **immediate post tx**: NR  **Short term**: [SF-mental]IG= 48.8(9.8); CG-ex= 49.2(10.9);CG-wt= 49.8(12.6)  [SF-physical] IG= 30.4(7.4); CG-ex= 30.3(7.8);CG-wt= 28.6(9.7)  **intermediate**: [SF-mental]IG= 43.5(10.8);CG-ex=45.5(10.8);CG-wt= 44.4(10.7)  [SF-physical]IG=31.4(7.7); CG-ex=29.3(8.5);CG-wt=31.5(8.3)  **Long term**: NR  **Harms**: 5 side-effects were reported by 4 pts(10%) in the qigong group (2 nausea, 2 aching muscles, 1 muscle tension) and 4 side effects by 2 pats in the ex-therapy group (2 muscle tensions, 1 aching muscles, 1 nausea). | |
| **Rendant, 2011 [**[**86**](#_ENREF_86)**] Germany** | **Trial Design:** RCT  **Tx duration**: 6 mo(s)  **Fu duration**  (last assessment): 6mo(s)  N screened: 231  N randomized: 123  N completed tx: 122  N attended last fu: 111  **Inclusion**: 20-60 yrs, duration of neck pain 6 mo(s)-5 yrs, average NP≥40mm(VAS). normal cervical spine ﬂexibility, predominantly NP.  **Exclusion**:  acute or chronic disorders (physical and mental) that disqualiﬁed, pregnancy, had qigong or exercise therapy during the last 6 months, whiplash or cancer causing NP, inﬂammatory arthritis column-surgery or prolapsed vertebral disc, analgesics, planned start of physiotherapy, taking up activities or in another study during the last 6 months. | **Mean age (SD/range)**:  mean age 45.6(10.7) yrs  **% of female**:87.7%  **Racial composition**: NR  **Other socio-demographics**:  Living partnership(76.2%),  **Co morbidities**:  53.3% reported concomitant diseases  **Prior episode of**  **pain if acute**: NR  **Prior CAM intervention**: last 3 months before study entry, 78.7% had consulted at least 1 physiotherapist for their neck pain  **Prior surgery related to current complaint**: NR | **Cause of Pain**: NR  **Duration of Pain**:  IG= 3.4(1.5); CG-ex= 3.2(1.6);CG-wt= 2.9(1.6)  **Severity of pain (Grading)**:  mean±SD for average NP, week before the baseline measure, 56.2±14.1 mm.VAS.  **Co-interventions**: NR | **Groups**  **IG** (n = 42)– qigong (Neiyanggong): 18 sessions (each 90 minutes), period of 6mo（s）,1 session/wk(first 3mo(s)),1session/2wks(second 3mo(s))  **Drop outs**: A =?, B =?,C=1,D=2  **CG** (n = 39) –exercise: 18 sessions (each 90 minutes), period of 6mo（s）,1 session/wk (first3mo(s)),1session/2wks(second 3mo(s))  **Drop outs**: A =?, B =?,C=3,D=1  CG(n=41)—waitlist: did not receive qigong or exercise therapy for the whole period  **Drop outs**: A =?, B =?,C=0,D=2  Note: In all groups, Pts free to do qigong or exercise in itself group had taught daily. | **Outcomes:**  **Pain**: VAS(0-100)  N based on ITT  **Disability**: NPAD (0-100)  N based on ITT  **Results-Baseline:** mean±SD  Pain**:** IG=57.7（13.5）; CG-ex=57.5（15.5）;CG-wt= 53.4（13.2）  Disability: IG= 44.0（12.7）; CG-ex= 39.5（15.4）;CG-wt= 43.2（16.1）  **Immediate post tx**: NR  **Short term**: Pain**:** IG= 28.7(22.6); CG-ex= 27.4(21.5);CG-wt=47.3(17.2)  Disability: IG= 32.8(14.2); CG-ex= 31.3 (15.7);CG-wt= 41.3(10.9)  **Intermediate**:  Pain**:** IG=26.7(20.3); CG-ex= 27.4(18.7);CG-wt=38.1(14.2)  Disability: IG= 30(10.7); CG-ex= 31.5(15.9);CG-wt= 29.1(14.2)  **Long term**: NR | **Outcome**  **instruments**:  **QoL/ well being**:  SF-36(0 to 100)mental component, physical component  **Other**: ADS(0-60) depression  **Results:** mean±SD  **Baseline**: [SF-mental]IG= 46.0（9.6）; CG-ex= 45.5（11.8）;CG-wt= 48.6 （9.8）  [SF-physical] IG= 43.1（7.5）; CG-ex= 43.7（ 6.9）;CG-wt= 43.3（7.8）  **immediate post tx**:  **Short term**: [SF-mental]IG= 50(9.4); CG-ex= 46.5(10.6);CG-wt= 45.1(8.4)  [SF-physical] IG= 46.6(7.1); CG-ex= 45.2(9.1);CG-wt= 43.2(6.8)  **intermediate**: [SF-mental]IG= 47.4(10.6);CG-ex=47.8(9.6);CG-wt= 45.4(9.1)  [SF-physical]IG=47(7.9); CG-ex=44.7(8.3);CG-wt= 43.1(7.4)  **Long term**: NR  **Harms**:muscle soreness, myogelosis, vertigo, other pain, headache, thirst, engorged hands, twinge in the neck， urinary urgency, bursitis of left shoulder. （both in qigong and EX groups） | |
| **Tai Chi for CLBP** |  |  |  |  |  |  | |
| **Hall, 2011 [**[**96**](#_ENREF_96)**] Australia** | **Trial Design:** RCT  **Tx duration**: 10wk(s)  **Fu duration**  (last assessment): immediately posttreatment, no follow-up  N screened: 412  N randomized: 160  N completed tx: 149  N attended last fu: 149  **Inclusion**: 18 and 70 years with persistent(≥3mo(s)) nonspeciﬁc low back pain±leg pain. response to questions 7 or 8 on the SF-36.  **Exclusion**:   1. known or suspected serious spinal pathology, 2) any contraindication to exercise, 3) scheduled for spinal surgery. | **Mean age**  **(SD/range)**: IG = 43.4(13.5) vs. CG = 44.3(13.0)yrs  **% of female**: 74.4%  **Racial composition**: a general community setting in Sydney, New South Wales, Australia.  **Other socio-demographics**: NR  **Co morbidities**: NR  **Prior episode of**  **pain if acute**: IG = 64Pt(s); CG = 59Pt(s)  **Prior CAM intervention**: IG= 67 Pt(s); CG= 64 Pt(s)  **Prior surgery related to current complaint**: None | **Cause of Pain**:  NR  **Duration of Pain**:  chronic, IG＞3 mo(s); CG ＞3 mo(s)  **Severity of pain (Grading)**: CPGQ, IG= 30.6(11.0); CG= 29.6 (11.6)  **Co-interventions**: NR | **Groups**  **IG** (n = 80)– tai chi(Sun style); 18 40-minute sessions over a 10-week period by a certiﬁed tai chi instructor, generally 2 sessions/wk for 8 wks followed by 1 sessions/wk for 2 wks, continued with their usual health care  **Drop outs**: A = 2, B = 4  **CG** (n = 90) –waitlist: continued with their usual health care  **Drop outs**: A = 0, B = 5 | **Outcomes:**  **Pain**: numerical rating scale (NRS) (0–10)  N based on ITT  **Disability**: PDI (0-70)  N based on ITT  **Results-Baseline:**  Pain: IG = 4.4 (4.0, 4.9),  CG = 4.44 (3.98, 4.89)  Disability: IG = 22.7 (19.8, 25.7), CG = 23.9 (20.9, 26.9)  **Immediate post tx**:  Pain: IG = 3.4 (2.91, 3.8),  CG = 4.7 (4.2, 5.1)  Disability: IG = 17.0 (13.9, 20.0), CG = 23.8 (20.7, 27.0)  **Short term**: NR  **Intermediate**: NR  **Long term**: NR | **Outcome**  **instruments**:  **QoL/ well being**: GPE (-5to --+5)  **Other**: disability (PDI, NPQ) improvement  **Results:**  **Baseline**: IG=0.4 (-0.1, 0.8); CG= -0.1 (-0.6, 0.8)  **immediate post tx**: IG= 1.6 (1.2, 2.1); CG= 0.4 (-0.1, 0.8)  **Short term**: NR  **Intermediate**: NR  **Long term**: NR  **Harms**: 3 pt(s) reported a small initial increase in BP symptoms, 1 pt reported an increase in upper BP | |
| **Chinese herbal medicine for NP** |  |  |  |  |  |  | |
| **Li, 2007[**[**90**](#_ENREF_90)**] China** | **Trial Design:** RCT  **Tx duration**: 4 wks  **Fu duration** (last assessment): immediate post-tx  N screened: 360  N randomized:360  N completed tx: 360  N attended last fu: 360  **Inclusion**:(a)18 to 60 yrs; (b) neck pain or stiffness, >=1 monthly recurrence, >=6 mons; confirmed by X-ray  **Exclusion**:  NR | **Mean age (SD/range)**:  IG= 50.8±9.3 yrs; CG= 18.4±5.1 yrs  **% of female**: 48.3%  **Racial composition**: NR  **Other socio-demographics**: NR  **Co morbidities**: NR  **Prior episode of pain if acute**: NR  **Prior CAM intervention**: NR  **Prior surgery related to current complaint**: NR | **Cause of Pain**:  Chronic neck  **Duration of Pain**:  IG= 18.4±5.1mons  CG=17.8±5.9 mons  **Severity of pain (Grading)**:  NR  **Co-interventions**: NR | **Groups**  **IG** (n = 180) –Extractum Nucis Vomicae: made in the Shenzhen Hospital of Traditional Chinese Medicine, composed of Maqianzi(Semen Strychni), Chanshu(Venenum Bufonis), Honghua(Flos Carthami), Chuanwu(Radix Aconiti), and Tiannanxing(Rhizoma Pinelliae seu Arisaematis). 3 times daily, for 4 weeks  **Drop outs**: A = 0  **CG** (n =180) –Diclofenac Diethylamine Emulgel: 3 times a day for 4 weeks (Dose and composition not reported)  **Drop outs**: A = 0 | **Outcomes:**  **Pain**: NRS (0-3),  Not based on ITT  **Disability**: NR  **Results-Baseline:** Pain**:** IG = 2.73±0.52, CG = 2.58±0.62  **Immediate post tx**:  Pain**:** IG = 0.59±0.14, CG = 0.86±0.21  **Short term**:  NR | **Outcome**  **instruments**:  **QoL/ well being**: NR  **Harms**: Herbal treatment, 14 cases had pruritus, 9 cases had reddish skin, and among them 2 cases had small blister on the skin. Diclofenac Diethylamine Emulgel, 10 cases had pruritus, and 6 cases had reddish skin. | |
| **Wang, 2004$** | **Trial Design:** RCT  **Tx duration**: 4 wks  **Fu duration** (last assessment): immediate post-tx  N screened: 240  N randomized: 240  N completed tx: 240  N attended last fu: 217  **Inclusion**:(a) 25 to 65 yrs; (b) neck pain or stiffness, disc herniation confirmed by X-ray  **Exclusion**:  NR | **Mean age (SD/range)**:  IG= 54.86±7.47 yrs; CG= 54.65±8.90 yrs  **% of female**: 59.6%  **Racial composition**: NR  **Other socio-demographics**: NR  **Co morbidities**: NR  **Prior episode of pain if acute**: NR  **Prior CAM intervention**: NR  **Prior surgery related to current complaint**: NR | **Cause of Pain**:  Chronic neck  **Duration of Pain**:  IG= 5.06±4.62 years  CG=4.94±4.16 years  **Severity of pain (Grading)**:  NR  **Co-interventions**: NR | **Groups**  **IG** (n = 120) –Qishe: Compound Qishe Tablet 25 tablets twice a day for 4 weeks  **Drop outs**: A = 11  **CG** (n =120) –Placebo: Placebo 25 tablets twice a day for 4 weeks  **Drop outs**: A = 12 | **Outcomes:**  **Pain**: NRS (0-3),  Not based on ITT  **Disability**: NR  **Results-Baseline:** NR  **Immediate post tx**:  Pain**:** IG = 0.71±0.54, CG = 1.38±0.61  **Short term**:  NR | **Outcome**  **instruments**:  **QoL/ well being**: NR  **Harms**: Index treatment: 2 cases with diarrhea, 1 case with abdominal pain and diarrhea, and 1 case with stomachache. Comparison treatment: 1 case with diarrhea and abdominal pain. | |
| **Wang, 2005$** | **Trial Design:** RCT  **Tx duration**: 4 wks  **Fu duration** (last assessment): immediate post-tx  N screened: 440  N randomized: 440  N completed tx: 440  N attended last fu: 416  **Inclusion**:(a) 29 to 63 yrs; (b) neck pain or stiffness, disc herniation confirmed by X-ray  **Exclusion**:  NR | **Mean age (SD/range)**:  IG= 49.71±11.07 yrs; CG= 50.45±10.78 yrs  **% of female**: 67.5%  **Racial composition**: NR  **Other socio-demographics**: NR  **Co morbidities**: NR  **Prior episode of pain if acute**: NR  **Prior CAM intervention**: NR  **Prior surgery related to current complaint**: NR | **Cause of Pain**:  Chronic neck  **Duration of Pain**:  IG=4.77±5.42 years  CG=4.89±5.26years  **Severity of pain (Grading)**:  NR  **Co-interventions**: NR | **Groups**  **IG** (n = 120) –Qishe plus placebo Jingfukang: Compound Qishe Tablet 1 bag (25 tablets) twice a day for 4 weeks with placebo Jingfukang 1 bag (granules) twice a day for 4 weeks  **Drop outs**: A = 19  **CG** (n =120) –placebo Qishe plus Jingfukang: Placebo Qishe Tablet 1 bag (25 tablets) twice a day for 4 weeks with Jingfukang 1 bag (granules) twice a day for 4 weeks  **Drop outs**: A = 5 | **Outcomes:**  **Pain**: NRS (0-3),  Not based on ITT  **Disability**: NR  **Results-Baseline:** NR  **Immediate post tx**:  Pain**:** IG = 0.81±0.44, CG = 0.89±0.47  **Short term**:  NR | **Outcome**  **instruments**:  **QoL/ well being**: NR  **Harms**: Index treatment, 11 cases with diarrhea, 9 cases with abdominal pain, and 2 cases with stomachache. Comparison treatment, 1 case with diarrhea, 1 case with stomachache. | |
| **Chinese Manipulation for NP** | | | | | | | |
| **Lin, 2013[**[**91**](#_ENREF_91)**] China** | **Trial Design:** RCT  **Tx duration**: 4 wks  **Fu duration** (last assessment): 3 mons  N screened: 75  N randomized: 63  N completed tx: 40  N attended last fu: 40  **Inclusion**: (1) NP without neurologic or vascular deficit, (2) Identified by static or motion palpation, (3) discomfort with joint pressure, (4) abnormal changes in radiological test, (5) NP referred from peripheral joints or viscera, rheumatic fibromyalgia and neurasthenia  **Exclusion**:  (1) contraindications to manipulation, (2) history of whiplash or surgery to the neck, (3) congenital abnormality (4) ervical radiculopathy or myelopathy, (5) cardiac disease, (6) having received LM or other bone-setting treatment in the past 3 months. | **Mean age (SD/range)**:  IG=38.94(11.71); CG=40.90(11.80)  **% of female**: 73%  **Racial composition**: NR  **Other socio-demographics**:  NR  **Co morbidities**: NR  **Prior episode of pain if acute**: NR  **Prior CAM intervention**: NR  **Prior surgery related to current complaint**: NR | **Cause of Pain**:  Non specific  **Duration of Pain**:  IG=37.06(35.20); CG=39.23(28.73)mo  **Severity of pain (Grading)**:  **Co-interventions**: NR | **Groups**  IG (n = 33–Long’s manipulation: there are totally four steps, Relaxation, Manipulation, Reinforcing, Painful region massage step; 20 min, 3 d interval, 8 sessions  **Drop outs**: A =2, B =4  **CG** (n = 30–traditional Chinese massage: performed through the step 1, 3 and 4 as aforementioned for IG.  **Drop outs**: A = 4, B =13 | **Outcomes:**  **Pain**: NRS (0-10),  Not based on ITT  **Disability**: NPQ  Not based on ITT  **Results-Baseline:** mean (SD)  Pain**:** IG=5.79(1.96), CG=5.63(1.90)  Disability: IG=35.44(14.05), CG=36.14(14.23)  **Immediate post tx**:  Pain**:** IG=2.06(1.65), CG=4.04(1.59)  Disability: IG=12.08(7.30), CG=21.43(11.18)  **Short term**:  Pain**:** IG=2.07(1.44), CG=4.54(2.26)  Disability: IG=15.07(7.47), CG=25.88(11.91) | | **Outcome**  **instruments**:  **QoL/ well being**: NR  **Other**: NR  **Results:**  **Baseline**: NR  **immediate post tx**: NR  **Short term**: NR  **intermediate**: NR  **Long term**: NR  **Harms**: there is no serious adverse event reported and the increased pain experienced by the patient in the TCM group relieved within one day |
| **Chen, 2009[**[**92**](#_ENREF_92)**] China** | **Trial Design:** RCT  **Tx duration**: 12-36 d  **Fu duration** (last assessment): immediate post-tx  N screened: unclear  N randomized: 120  N completed tx: 120  N attended last fu: 120  **Inclusion**: cervical spondylotic radiculopathy, age>=18  **Exclusion**:  NR | **Mean age (SD/range)**:  IG=22-42; CG=20-40 yrs  **% of female**: Unclear  **Racial composition**: NR  **Other socio-demographics**:  NR  **Co morbidities**: NR  **Prior episode of pain if acute**: NR  **Prior CAM intervention**: NR  **Prior surgery related to current complaint**: NR | **Cause of Pain**:  cervical spondylotic radiculopathy **Duration of Pain**:  acute-chronic  **Severity of pain (Grading)**:  **Co-interventions**: NR | **Groups**  IG (n = 60)–Chinese manipulation: technique, transverse thrust; frequency, 1 session/2 days; dose, 12~36 days; route, spinous process of cervical spine  **Drop outs**: A =0  **CG** (n = 60)–traditional Chinese massage: technique, rolling; frequency, 1 session/2 days; dose, 12~36 days; route, muscles in cervical region  **Drop outs**: A =0 | **Outcomes:**  **Pain**: NRS (0-10),  Not based on ITT  **Disability**: NR  **Results-Baseline:** mean (SD)  Pain**:** IG=5.81, CG=5.08  **Immediate post tx**:  Pain**:** IG=1.78(1.76), CG=3.8(2.42)  **Short term**:  NR | | **Outcome**  **instruments**:  **QoL/ well being**: NR  **Other**: NR  **Results:**  **Baseline**: NR  **immediate post tx**: NR  **Short term**: NR  **intermediate**: NR  **Long term**: NR  **Harms**: there is no serious adverse event reported |
| **Zhu, 2005[**[**93**](#_ENREF_93)**] China** | **Trial Design:** RCT  **Tx duration**: 4 wks  **Fu duration** (last assessment): immediate post-tx  N screened: unclear  N randomized: 213  N completed tx: 213  N attended last fu: 213  **Inclusion**: cervical spondylotic radiculopathy, age>=20  **Exclusion**:  NR | **Mean age (SD/range)**:  IG=25-65; CG=27-65 yrs  **% of female**: Unclear  **Racial composition**: NR  **Other socio-demographics**:  NR  **Co morbidities**: NR  **Prior episode of pain if acute**: NR  **Prior CAM intervention**: NR  **Prior surgery related to current complaint**: NR | **Cause of Pain**:  cervical spondylotic radiculopathy **Duration of Pain**:  acute-chronic  **Severity of pain (Grading)**:  **Co-interventions**: NR | **Groups**  IG (n = 115–Chinese manipulation: technique, rotatory traction; frequency, 2 sessions/week; dose, 4 weeks; route, cervical spine **Drop outs**: A =0  **CG** (n = 98–cervical traction: in sitting: frequency, 3 sessions/week; dose, 3~6 kg, 20 min/ session for 4 weeks; route, cervical spine  **Drop outs**: A = 0 | **Outcomes:**  **Pain**: VAS (0-10),  Not based on ITT  **Disability**: NR  **Results-Baseline:** mean (SD)  Pain**:** IG=4.46, CG=4.51  **Immediate post tx**:  Pain**:** IG=1.89(1.09), CG=2.95(1.21)  **Short term**:  NR | | **Outcome**  **instruments**:  **QoL/ well being**: NR  **Other**: NR  **Results:**  **Baseline**: NR  **immediate post tx**: NR  **Short term**: NR  **intermediate**: NR  **Long term**: NR  **Harms**: there is no serious adverse event reported |

$ study was unpublished.

**Outcomes:** ODQ = Oswestry disability questionnaire; RMQ = Roland Morris Questionnaire; RMDQ = Roland Morris Disability Questionnaire; NPQ = Northwick Neck Pain Questionnaire; MPQ = McGill Pain Questionnaire; ODI = Oswestry Disability Index; mODQ = modified Oswestry Disability Questionnaire; ODQ = Oswestry Disability Questionnaire; NDI = Neck Disability Index; PDI = pain disability index; GPE=global perceived effect; PPI = present pain intensity; PRI = pain rating index; MRP = motion related pain; NPAD = Neck Pain and Disability Scale; QoL = Quality of Life; MVEE = maximum voluntary extension effort; PQ = pain questionnaire; MPQ = Short Form McGill Pain Questionnaire; QBPDS = Quebec Back Pain Disability Scale; mRDQ = modified Roland Morris Questionnarie NRS = numeric pain rating scale; PPT = pressure pain threshold; VAS = visual analogue scale; PSFS = Patient-Speciﬁc Functional Scale; GPE = 11-point global perceived effect; PDI = Pain Disability Index; NPAD = Neck Pain and Disability Scale; SF-36 = 36-item short-form questionnaire; PC = physical component; MC = mental component; GH = general health; SF-PQ = Short-Form Pain Questionnaires; VRS-Chinese = Chinese Pain Intensity Verbal Rating Scale; BPI = Brief Pain Inventory Short Form; PPI = Present Pain Intensity Scale of the McGill Pain Questionnaire; MQS = Medication Quantiﬁcation Scale Version III; EQ-5D = EuroQol health index. **Special terms:** HVLA = high velocity low amplitude; ETOIMS = electrical twitch-obtaining intramuscular stimulation; IMS = intramuscular stimulation; FDT = flexion distraction technique; TrP = trigger point; GP = general practitioner; CAM = complementary and alternative medicine; NSAIDs = non-steroidal anti-inflammatory drugs; NP = neck pain; N-S = non-specific; S = specific; Med = medication; PT = physiotherapy; ST = standard therapy; E-acu = electro acupuncture; MR = muscle relaxation; EX = exercise; WT = waitlist; CLBP = chronic low back pain; CNP = chronic neck pain; A = baseline evaluation; B = immediately post treatment; C = short term follow up (up to 3 months post treatment); D = intermediate follow up (up to 6 months post treatment); E = long term follow up (over 6 months post treatment); acu = acupuncture; SM = spinal manipulation; LBP = low back pain; NP = neck pain; TP = thoracic pain TENS/TNS = transcutaneous electrical nerve stimulation; ROM = range of motion; MPS = myofascial pain syndrome; Mob = mobilization; ext = extension; flx = flexion; rot = rotation; MS = MS; PM = physical modalities; mA = milli Amp; Statistical: NS = statistically non-significant; SD = standard deviation; SE = standard error; WMD = weighted mean difference; p = p-value; 95% CI = 95% Confidence Interval; SS = statistically significant. **General terms:** NA = not available/applicable; NR = not reported; Pt(s) = patient(s); d = day(s); mo(s) = month(s); yr(s) = year(s); wk(s) = week(s); N = number NS = not significant; pt/s = patient/s; tx = treatment/intervention Fu = follow up; ITT = intention to treat; IG = intervention group; CG = control group; RCT = randomized controlled trial; AE(s) = adverse event(s); SAE = serious adverse events; WDAE = withdrawal due to adverse events.

$ study was unpublished.
