# Supplementary material for: Traditional Chinese Medicine for Neck Pain and Low Back Pain: A Systematic Review and Meta-Analysis
Source: PLoS One. 2015 Feb 24;10(2):e0117146. doi: 10.1371/journal.pone.0117146 (PMC4339195; doi:10.1371/journal.pone.0117146)
Supplement: S7 Table — (DOCX) [file pone.0117146.s008.docx]

**S7 Table.** The Risk of Bias and Quality of Individual Studies Included.

| **Author and year** | **Q1. Randomization Adequate?** | **Q2. Treatment Allocation Concealed?** | **Q3. Groups Similar at Baseline** | **Q4. Patient Blinded to the Intervention?** | **Q5. Care Provider Blinded to the Intervention?** | **Q6. Outcome Assessor Blinded to the Intervention?** | **Q7. Co- interventions Avoided or Similar?** | **Q8. Compliance Acceptable in All Groups** | **Q9. Dropout Rate Described and Acceptable?** | **Q10. Timing of the Outcome Assessment in All Groups Similar?** | **Q11. Analysis Includes an Intention-to-treat analysis?** | **Q12. Reports of the Study Free of Suggestion of Selective Outcome Reporting?** | **Total Score** | **Quality (Q2-4, 9)** |
| --- | --- | --- | --- | --- | --- | --- | --- | --- | --- | --- | --- | --- | --- | --- |
| **Acupuncture in NP** |  |  |  |  |  |  |  |  |  |  |  |  |  |  |
| Liang, 2011 [[21](file:///C:\Users\Administrator\Desktop\PLOSone回编辑tou\正文\Traditional%20Chinese%20Medicine%20for%20Neck%20Pain%20and%20Low%20Back%20Pain1.docx#_ENREF_21)] | Y | Y | Y | Y | N | N | ? | ? | Y | Y | N | Y | 7 | Good |
| Sahin, 2010 [[22](file:///C:\Users\Administrator\Desktop\PLOSone回编辑tou\正文\Traditional%20Chinese%20Medicine%20for%20Neck%20Pain%20and%20Low%20Back%20Pain1.docx#_ENREF_22)] | ? | Y | Y | Y | N | Y | ? | ? | Y | Y | N | Y | 7 | Good |
| Fu, 2009 [[23](file:///C:\Users\Administrator\Desktop\PLOSone回编辑tou\正文\Traditional%20Chinese%20Medicine%20for%20Neck%20Pain%20and%20Low%20Back%20Pain1.docx#_ENREF_23)] | Y | Y | Y | ? | N | ? | Y | ? | Y | Y | N | Y | 7 | Fair |
| Itoh, 2007 [[24](file:///C:\Users\Administrator\Desktop\PLOSone回编辑tou\正文\Traditional%20Chinese%20Medicine%20for%20Neck%20Pain%20and%20Low%20Back%20Pain1.docx#_ENREF_24)] | ? | ? | Y | Y | N | Y | ? | ? | Y | Y | N | Y | 6 | Fair |
| Nabeta, 2002 [[28](file:///C:\Users\Administrator\Desktop\PLOSone回编辑tou\正文\Traditional%20Chinese%20Medicine%20for%20Neck%20Pain%20and%20Low%20Back%20Pain1.docx#_ENREF_28)] | ? | ? | ? | Y | N | ? | ? | Y | Y | Y | Y | N | 5 | Fair |
| Zhu, 2002 [[27](file:///C:\Users\Administrator\Desktop\PLOSone回编辑tou\正文\Traditional%20Chinese%20Medicine%20for%20Neck%20Pain%20and%20Low%20Back%20Pain1.docx#_ENREF_27)] | ? | Y | Y | N | N | Y | ? | Y | Y | Y | ? | Y | 7 | Fair |
| Birch, 1998[[30](file:///C:\Users\Administrator\Desktop\PLOSone回编辑tou\正文\Traditional%20Chinese%20Medicine%20for%20Neck%20Pain%20and%20Low%20Back%20Pain1.docx#_ENREF_30)] | ? | ? | Y | N | N | ? | ? | N | N | Y | ? | N | 2 | Poor |
| Vas, 2006 [[25](file:///C:\Users\Administrator\Desktop\PLOSone回编辑tou\正文\Traditional%20Chinese%20Medicine%20for%20Neck%20Pain%20and%20Low%20Back%20Pain1.docx#_ENREF_25)] | ? | Y | Y | N | N | Y | ? | ? | Y | Y | Y | Y | 7 | Fair |
| White, 2004[[26](file:///C:\Users\Administrator\Desktop\PLOSone回编辑tou\正文\Traditional%20Chinese%20Medicine%20for%20Neck%20Pain%20and%20Low%20Back%20Pain1.docx#_ENREF_26)] | Y | Y | Y | Y | N | ? | Y | Y | Y | Y | Y | N | 9 | Good |
| Petrie, 1986[[31](file:///C:\Users\Administrator\Desktop\PLOSone回编辑tou\正文\Traditional%20Chinese%20Medicine%20for%20Neck%20Pain%20and%20Low%20Back%20Pain1.docx#_ENREF_31)] | ? | ? | N | Y | N | ? | ? | Y | Y | Y | ? | Y | 5 | Fair |
| Irnich, 2001[[29](file:///C:\Users\Administrator\Desktop\PLOSone回编辑tou\正文\Traditional%20Chinese%20Medicine%20for%20Neck%20Pain%20and%20Low%20Back%20Pain1.docx#_ENREF_29)] | ? | ? | N | N | ? | ? | ? | Y | Y | Y | Y | N | 4 | Poor |
| Zhang, 2003[[36](file:///C:\Users\Administrator\Desktop\PLOSone回编辑tou\正文\Traditional%20Chinese%20Medicine%20for%20Neck%20Pain%20and%20Low%20Back%20Pain1.docx#_ENREF_36)] | Y | Y | Y | ? | ? | ? | Y | Y | N | Y | N | ? | 6 | Fair |
| Thomas, 1991[[34](file:///C:\Users\Administrator\Desktop\PLOSone回编辑tou\正文\Traditional%20Chinese%20Medicine%20for%20Neck%20Pain%20and%20Low%20Back%20Pain1.docx#_ENREF_34)] | ? | ? | Y | N | N | N | ? | Y | Y | Y | ? | N | 4 | Fair |
| Giles, 2003[[32](file:///C:\Users\Administrator\Desktop\PLOSone回编辑tou\正文\Traditional%20Chinese%20Medicine%20for%20Neck%20Pain%20and%20Low%20Back%20Pain1.docx#_ENREF_32)] | Y | Y | N | N | N | Y | N | N | N | Y | Y | N | 5 | Poor |
| Giles, 1999[[33](file:///C:\Users\Administrator\Desktop\PLOSone回编辑tou\正文\Traditional%20Chinese%20Medicine%20for%20Neck%20Pain%20and%20Low%20Back%20Pain1.docx#_ENREF_33)] | ? | ? | N | N | N | ? | ? | N | N | ? | N | Y | 1 | Poor |
| Li, 2006[[35](file:///C:\Users\Administrator\Desktop\PLOSone回编辑tou\正文\Traditional%20Chinese%20Medicine%20for%20Neck%20Pain%20and%20Low%20Back%20Pain1.docx#_ENREF_35)] | ? | ? | Y | N | N | N | ? | ? | Y | Y | N | ? | 3 | Fair |
| Coan, 1981[[37](file:///C:\Users\Administrator\Desktop\PLOSone回编辑tou\正文\Traditional%20Chinese%20Medicine%20for%20Neck%20Pain%20and%20Low%20Back%20Pain1.docx#_ENREF_37)] | Y | Y | N | ? | ? | ? | ? | Y | Y | N | ? | N | 4 | Fair |
| **Acupuncture in LBP** |  |  |  |  |  |  |  |  |  |  |  |  |  |  |
| Miyazaki, 2009 [[39](file:///C:\Users\Administrator\Desktop\PLOSone回编辑tou\正文\Traditional%20Chinese%20Medicine%20for%20Neck%20Pain%20and%20Low%20Back%20Pain1.docx#_ENREF_39)] | ? | ? | Y | Y | Y | Y | Y | Y | Y | Y | Y | Y | 10 | Fair |
| Cherkin, 2009 [[40](file:///C:\Users\Administrator\Desktop\PLOSone回编辑tou\正文\Traditional%20Chinese%20Medicine%20for%20Neck%20Pain%20and%20Low%20Back%20Pain1.docx#_ENREF_40)] | ? | ? | ? | N | N | Y | Y | Y | Y | Y | Y | N | 6 | Poor |
| Haake, 2007[[41](file:///C:\Users\Administrator\Desktop\PLOSone回编辑tou\正文\Traditional%20Chinese%20Medicine%20for%20Neck%20Pain%20and%20Low%20Back%20Pain1.docx#_ENREF_41)] | Y | Y | Y | Y | N | Y | ? | ? | Y | Y | Y | Y | 9 | Good |
| Itoh, 2006[[42](file:///C:\Users\Administrator\Desktop\PLOSone回编辑tou\正文\Traditional%20Chinese%20Medicine%20for%20Neck%20Pain%20and%20Low%20Back%20Pain1.docx#_ENREF_42)] | Y | Y | Y | Y | N | Y | ? | ? | Y | Y | N | Y | 8 | Good |
| Inoue, 2006[[43](file:///C:\Users\Administrator\Desktop\PLOSone回编辑tou\正文\Traditional%20Chinese%20Medicine%20for%20Neck%20Pain%20and%20Low%20Back%20Pain1.docx#_ENREF_43)] | Y | Y | Y | Y | N | Y | ? | ? | Y | Y | Y | Y | 9 | Good |
| Brinkaus, 2006[[44](file:///C:\Users\Administrator\Desktop\PLOSone回编辑tou\正文\Traditional%20Chinese%20Medicine%20for%20Neck%20Pain%20and%20Low%20Back%20Pain1.docx#_ENREF_44)] | Y | Y | Y | Y | N | ? | ? | ? | Y | Y | Y | Y | 8 | Good |
| Itoh, 2004[[45](file:///C:\Users\Administrator\Desktop\PLOSone回编辑tou\正文\Traditional%20Chinese%20Medicine%20for%20Neck%20Pain%20and%20Low%20Back%20Pain1.docx#_ENREF_45)] | Y | ? | Y | Y | N | Y | ? | ? | Y | Y | N | Y | 7 | Fair |
| Molsberger, 2002[[46](file:///C:\Users\Administrator\Desktop\PLOSone回编辑tou\正文\Traditional%20Chinese%20Medicine%20for%20Neck%20Pain%20and%20Low%20Back%20Pain1.docx#_ENREF_46)] | ? | N | Y | N | N | N | ? | N | N | Y | Y | ? | 3 | Poor |
| Leibing, 2002[[47](file:///C:\Users\Administrator\Desktop\PLOSone回编辑tou\正文\Traditional%20Chinese%20Medicine%20for%20Neck%20Pain%20and%20Low%20Back%20Pain1.docx#_ENREF_47)] | ? | ? | Y | ? | N | ? | N | N | N | Y | N | N | 2 | Poor |
| Hasegawa, 2013[[48](file:///C:\Users\Administrator\Desktop\PLOSone回编辑tou\正文\Traditional%20Chinese%20Medicine%20for%20Neck%20Pain%20and%20Low%20Back%20Pain1.docx#_ENREF_48)] | Y | Y | Y | Y | N | Y | Y | Y | Y | Y | Y | Y | 11 | Good |
| Su, 2010[[50](file:///C:\Users\Administrator\Desktop\PLOSone回编辑tou\正文\Traditional%20Chinese%20Medicine%20for%20Neck%20Pain%20and%20Low%20Back%20Pain1.docx#_ENREF_50)] | Y | Y | Y | Y | N | ? | Y | Y | Y | Y | Y | Y | 10 | Good |
| Kennedy, 2008[[51](file:///C:\Users\Administrator\Desktop\PLOSone回编辑tou\正文\Traditional%20Chinese%20Medicine%20for%20Neck%20Pain%20and%20Low%20Back%20Pain1.docx#_ENREF_51)] | Y | Y | N | N | N | Y | ? | Y | Y | Y | Y | Y | 8 | Fair |
| Zaringhalam, 2010[[52](file:///C:\Users\Administrator\Desktop\PLOSone回编辑tou\正文\Traditional%20Chinese%20Medicine%20for%20Neck%20Pain%20and%20Low%20Back%20Pain1.docx#_ENREF_52)] | Y | Y | Y | ? | ? | ? | ? | Y | Y | Y | Y | ? | 7 | Fair |
| Witt, 2006[[53](file:///C:\Users\Administrator\Desktop\PLOSone回编辑tou\正文\Traditional%20Chinese%20Medicine%20for%20Neck%20Pain%20and%20Low%20Back%20Pain1.docx#_ENREF_53)] | Y | Y | Y | N | N | Y | ? | ? | Y | Y | N | Y | 7 | Fair |
| Coan, 1980[[55](file:///C:\Users\Administrator\Desktop\PLOSone回编辑tou\正文\Traditional%20Chinese%20Medicine%20for%20Neck%20Pain%20and%20Low%20Back%20Pain1.docx#_ENREF_55)] | ? | ? | N | N | ? | ? | ? | Y | Y | ? | ? | Y | 3 | Poor |
| Itoh, 2009[[56](file:///C:\Users\Administrator\Desktop\PLOSone回编辑tou\正文\Traditional%20Chinese%20Medicine%20for%20Neck%20Pain%20and%20Low%20Back%20Pain1.docx#_ENREF_56)] | ? | ? | Y | N | N | ? | Y | Y | Y | Y | ? | Y | 6 | Fair |
| Grant, 1999[[57](file:///C:\Users\Administrator\Desktop\PLOSone回编辑tou\正文\Traditional%20Chinese%20Medicine%20for%20Neck%20Pain%20and%20Low%20Back%20Pain1.docx#_ENREF_57)] | Y | Y | N | N | N | Y | N | N | N | Y | Y | N | 5 | Poor |
| Muller, 2005[[58](file:///C:\Users\Administrator\Desktop\PLOSone回编辑tou\正文\Traditional%20Chinese%20Medicine%20for%20Neck%20Pain%20and%20Low%20Back%20Pain1.docx#_ENREF_58)] | Y | Y | Y | N | N | Y | ? | Y | Y | Y | ? | Y | 8 | Fair |
| Wang, 2004[[59](file:///C:\Users\Administrator\Desktop\PLOSone回编辑tou\正文\Traditional%20Chinese%20Medicine%20for%20Neck%20Pain%20and%20Low%20Back%20Pain1.docx#_ENREF_59)] | ? | ? | ? | N | N | ? | ? | ? | ? | Y | N | Y | 2 | Poor |
| Yun, 2012 [[129](file:///C:\Users\Administrator\Desktop\PLOSone回编辑tou\正文\Traditional%20Chinese%20Medicine%20for%20Neck%20Pain%20and%20Low%20Back%20Pain1.docx#_ENREF_129)] | Y | Y | Y | ? | N | Y | ? | ? | N | Y | ? | N | 5 | Fair |
| Shankar, 2011[[61](file:///C:\Users\Administrator\Desktop\PLOSone回编辑tou\正文\Traditional%20Chinese%20Medicine%20for%20Neck%20Pain%20and%20Low%20Back%20Pain1.docx#_ENREF_61)] | Y | ? | Y | ? | ? | ? | ? | ? | ? | N | Y | ? | 3 | Poor |
| Tsui, 2004[[62](file:///C:\Users\Administrator\Desktop\PLOSone回编辑tou\正文\Traditional%20Chinese%20Medicine%20for%20Neck%20Pain%20and%20Low%20Back%20Pain1.docx#_ENREF_62)] | ? | ? | Y | Y | ? | Y | ? | ? | Y | Y | ? | Y | 6 | Fair |
| Sator, 2004[[63](file:///C:\Users\Administrator\Desktop\PLOSone回编辑tou\正文\Traditional%20Chinese%20Medicine%20for%20Neck%20Pain%20and%20Low%20Back%20Pain1.docx#_ENREF_63)] | Y | Y | N | Y | N | Y | ? | Y | Y | Y | Y | Y | 9 | Fair |
| Yeung, 2003[[64](file:///C:\Users\Administrator\Desktop\PLOSone回编辑tou\正文\Traditional%20Chinese%20Medicine%20for%20Neck%20Pain%20and%20Low%20Back%20Pain1.docx#_ENREF_64)] | ? | ? | Y | N | N | ? | Y | Y | Y | Y | Y | Y | 7 | Fair |
| Meng, 2003[[65](file:///C:\Users\Administrator\Desktop\PLOSone回编辑tou\正文\Traditional%20Chinese%20Medicine%20for%20Neck%20Pain%20and%20Low%20Back%20Pain1.docx#_ENREF_65)] | Y | Y | Y | N | N | ? | Y | ? | Y | Y | Y | N | 7 | Fair |
| Hunter, 2012 [[66](file:///C:\Users\Administrator\Desktop\PLOSone回编辑tou\正文\Traditional%20Chinese%20Medicine%20for%20Neck%20Pain%20and%20Low%20Back%20Pain1.docx#_ENREF_66)] | Y | Y | Y | N | N | Y | ? | Y | Y | Y | Y | Y | 9 | Fair |
| Weiss, 2013 [[130](file:///C:\Users\Administrator\Desktop\PLOSone回编辑tou\正文\Traditional%20Chinese%20Medicine%20for%20Neck%20Pain%20and%20Low%20Back%20Pain1.docx#_ENREF_130)] | Y | ? | Y | N | N | ? | ? | ? | Y | Y | N | N | 4 | Fair |
| Cho, 2013 [[38](file:///C:\Users\Administrator\Desktop\PLOSone回编辑tou\正文\Traditional%20Chinese%20Medicine%20for%20Neck%20Pain%20and%20Low%20Back%20Pain1.docx#_ENREF_38)] | Y | Y | Y | Y | N | Y | Y | Y | Y | Y | N | N | 9 | Good |
| Vas, 2012[[49](file:///C:\Users\Administrator\Desktop\PLOSone回编辑tou\正文\Traditional%20Chinese%20Medicine%20for%20Neck%20Pain%20and%20Low%20Back%20Pain1.docx#_ENREF_49)] | Y | Y | Y | Y | N | Y | Y | ? | Y | Y | Y | Y | 9 | Good |
| **Acupressure** |  |  |  |  |  |  |  |  |  |  |  |  |  |  |
| Hsieh, 2004 [[67](file:///C:\Users\Administrator\Desktop\PLOSone回编辑tou\正文\Traditional%20Chinese%20Medicine%20for%20Neck%20Pain%20and%20Low%20Back%20Pain1.docx#_ENREF_67)] | Y | Y | Y | N | N | Y | ? | ? | Y | Y | Y | N | 7 | Fair |
| Hsieh, 2006 [[71](file:///C:\Users\Administrator\Desktop\PLOSone回编辑tou\正文\Traditional%20Chinese%20Medicine%20for%20Neck%20Pain%20and%20Low%20Back%20Pain1.docx#_ENREF_71)] | Y | Y | Y | N | N | Y | ? | ? | Y | Y | Y | Y | 8 | Fair |
| Suen, 2007 [[69](file:///C:\Users\Administrator\Desktop\PLOSone回编辑tou\正文\Traditional%20Chinese%20Medicine%20for%20Neck%20Pain%20and%20Low%20Back%20Pain1.docx#_ENREF_69)] | ? | ? | Y | Y | N | ? | Y | N | N | Y | N | N | 4 | Fair |
| Yeh, 2013 [[68](file:///C:\Users\Administrator\Desktop\PLOSone回编辑tou\正文\Traditional%20Chinese%20Medicine%20for%20Neck%20Pain%20and%20Low%20Back%20Pain1.docx#_ENREF_68)] | Y | ? | Y | Y | N | ? | ? | ? | Y | Y | N | Y | 6 | Fair |
| Yip, 2004 [[72](file:///C:\Users\Administrator\Desktop\PLOSone回编辑tou\正文\Traditional%20Chinese%20Medicine%20for%20Neck%20Pain%20and%20Low%20Back%20Pain1.docx#_ENREF_72)] | Y | ? | Y | N | N | ? | Y | ? | Y | Y | N | Y | 6 | Fair |
| Yip, 2006 [[70](file:///C:\Users\Administrator\Desktop\PLOSone回编辑tou\正文\Traditional%20Chinese%20Medicine%20for%20Neck%20Pain%20and%20Low%20Back%20Pain1.docx#_ENREF_70)] | ? | ? | Y | N | N | ? | Y | Y | Y | Y | Y | N | 6 | Fair |
| **Cupping in NP** |  |  |  |  |  |  |  |  |  |  |  |  |  |  |
| Lauche, 2011 [[95](file:///C:\Users\Administrator\Desktop\PLOSone回编辑tou\正文\Traditional%20Chinese%20Medicine%20for%20Neck%20Pain%20and%20Low%20Back%20Pain1.docx#_ENREF_95)] | Y | Y | Y | N | N | N | Y | ? | Y | Y | Y | Y | 8 | Fair |
| Lauche, 2012b [[74](file:///C:\Users\Administrator\Desktop\PLOSone回编辑tou\正文\Traditional%20Chinese%20Medicine%20for%20Neck%20Pain%20and%20Low%20Back%20Pain1.docx#_ENREF_74)] | Y | Y | Y | N | N | ? | Y | ? | Y | Y | Y | Y | 8 | Fair |
| Cramer, 2011 [[77](file:///C:\Users\Administrator\Desktop\PLOSone回编辑tou\正文\Traditional%20Chinese%20Medicine%20for%20Neck%20Pain%20and%20Low%20Back%20Pain1.docx#_ENREF_77)] | Y | Y | Y | N | N | N | Y | ? | Y | Y | Y | Y | 8 | Fair |
| Kim, 2011 [[76](file:///C:\Users\Administrator\Desktop\PLOSone回编辑tou\正文\Traditional%20Chinese%20Medicine%20for%20Neck%20Pain%20and%20Low%20Back%20Pain1.docx#_ENREF_76)] | Y | Y | Y | N | N | N | Y | ? | Y | Y | Y | Y | 8 | Fair |
| Lauche, 2013 [[73](file:///C:\Users\Administrator\Desktop\PLOSone回编辑tou\正文\Traditional%20Chinese%20Medicine%20for%20Neck%20Pain%20and%20Low%20Back%20Pain1.docx#_ENREF_73)] | Y | Y | Y | N | N | Y | ? | Y | Y | Y | Y | Y | 9 | Fair |
| **Cupping in LBP** |  |  |  |  |  |  |  |  |  |  |  |  |  |  |
| Xu, 2009[[78](file:///C:\Users\Administrator\Desktop\PLOSone回编辑tou\正文\Traditional%20Chinese%20Medicine%20for%20Neck%20Pain%20and%20Low%20Back%20Pain1.docx#_ENREF_78)] | ? | ? | Y | ? | N | ? | ? | ? | ? | Y | Y | ? | 3 | Poor |
| Li, 2009[[79](file:///C:\Users\Administrator\Desktop\PLOSone回编辑tou\正文\Traditional%20Chinese%20Medicine%20for%20Neck%20Pain%20and%20Low%20Back%20Pain1.docx#_ENREF_79)] | ? | ? | Y | ? | N | ? | ? | ? | ? | Y | Y | ? | 3 | Poor |
| Liu, 2008[[80](file:///C:\Users\Administrator\Desktop\PLOSone回编辑tou\正文\Traditional%20Chinese%20Medicine%20for%20Neck%20Pain%20and%20Low%20Back%20Pain1.docx#_ENREF_80)] | Y | ? | Y | ? | N | ? | ? | ? | ? | Y | Y | ? | 4 | Poor |
| Hong, 2006[[81](file:///C:\Users\Administrator\Desktop\PLOSone回编辑tou\正文\Traditional%20Chinese%20Medicine%20for%20Neck%20Pain%20and%20Low%20Back%20Pain1.docx#_ENREF_81)] | Y | ? | Y | ? | N | ? | ? | ? | ? | Y | Y | ? | 4 | Poor |
| Farhadi, 2009 [[83](file:///C:\Users\Administrator\Desktop\PLOSone回编辑tou\正文\Traditional%20Chinese%20Medicine%20for%20Neck%20Pain%20and%20Low%20Back%20Pain1.docx#_ENREF_83)] | ? | Y | Y | N | N | ? | Y | ? | Y | Y | N | Y | 6 | Fair |
| Kim, 2012 [[82](file:///C:\Users\Administrator\Desktop\PLOSone回编辑tou\正文\Traditional%20Chinese%20Medicine%20for%20Neck%20Pain%20and%20Low%20Back%20Pain1.docx#_ENREF_82)] | Y | Y | Y | N | N | ? | Y | ? | Y | Y | Y | Y | 8 | Fair |
| **Gua sha** |  |  |  |  |  |  |  |  |  |  |  |  |  |  |
| Braum, 2011 [[85](file:///C:\Users\Administrator\Desktop\PLOSone回编辑tou\正文\Traditional%20Chinese%20Medicine%20for%20Neck%20Pain%20and%20Low%20Back%20Pain1.docx#_ENREF_85)] | Y | Y | Y | N | N | Y | Y | ? | Y | Y | Y | Y | 9 | Fair |
| Lauche, 2012a [[84](file:///C:\Users\Administrator\Desktop\PLOSone回编辑tou\正文\Traditional%20Chinese%20Medicine%20for%20Neck%20Pain%20and%20Low%20Back%20Pain1.docx#_ENREF_84)] | Y | Y | Y | N | N | N | N | ? | Y | Y | Y | N | 6 | Fair |
| **Qigong** |  |  |  |  |  |  |  |  |  |  |  |  |  |  |
| Lansinger, 2007 [[88](file:///C:\Users\Administrator\Desktop\PLOSone回编辑tou\正文\Traditional%20Chinese%20Medicine%20for%20Neck%20Pain%20and%20Low%20Back%20Pain1.docx#_ENREF_88)] | ? | Y | Y | N | N | Y | Y | ? | Y | Y | Y | N | 7 | Fair |
| Von Trott, 2009 [[87](file:///C:\Users\Administrator\Desktop\PLOSone回编辑tou\正文\Traditional%20Chinese%20Medicine%20for%20Neck%20Pain%20and%20Low%20Back%20Pain1.docx#_ENREF_87)] | Y | Y | Y | N | N | ? | N | ? | Y | Y | Y | Y | 7 | Fair |
| Rendant, 2011 [[86](file:///C:\Users\Administrator\Desktop\PLOSone回编辑tou\正文\Traditional%20Chinese%20Medicine%20for%20Neck%20Pain%20and%20Low%20Back%20Pain1.docx#_ENREF_86)] | Y | Y | Y | N | N | ? | Y | ? | Y | Y | Y | Y | 8 | Fair |
| **Tai chi** |  |  |  |  |  |  |  |  |  |  |  |  |  |  |
| Hall, 2011 [[96](file:///C:\Users\Administrator\Desktop\PLOSone回编辑tou\正文\Traditional%20Chinese%20Medicine%20for%20Neck%20Pain%20and%20Low%20Back%20Pain1.docx#_ENREF_96)] | Y | Y | Y | N | N | Y | ? | N | Y | Y | Y | Y | 8 | Fair |
| **Herbal medicine** |  |  |  |  |  |  |  |  |  |  |  |  |  |  |
| Li, 2007[[90](file:///C:\Users\Administrator\Desktop\PLOSone回编辑tou\正文\Traditional%20Chinese%20Medicine%20for%20Neck%20Pain%20and%20Low%20Back%20Pain1.docx#_ENREF_90)] | Y | N | Y | N | N | N | ? | ? | Y | Y | N | ? | 4 | Fair |
| Wang, 2004$ | Y | N | Y | Y | Y | Y | ? | ? | ? | Y | N | N | 6 | Fair |
| Wang, 2005$ | Y | N | Y | Y | Y | Y | ? | ? | ? | Y | N | N | 6 | Fair |
| **Manipulation** |  |  |  |  |  |  |  |  |  |  |  |  |  |  |
| Lin, 2013[[91](file:///C:\Users\Administrator\Desktop\PLOSone回编辑tou\正文\Traditional%20Chinese%20Medicine%20for%20Neck%20Pain%20and%20Low%20Back%20Pain1.docx#_ENREF_91)] | Y | Y | Y | ? | N | ? | ? | ? | N | Y | Y | Y | 6 | Fair |
| Chen, 2009[[92](file:///C:\Users\Administrator\Desktop\PLOSone回编辑tou\正文\Traditional%20Chinese%20Medicine%20for%20Neck%20Pain%20and%20Low%20Back%20Pain1.docx#_ENREF_92)] | ? | N | Y | N | N | N | Y | N | Y | N | Y | ? | 4 | Poor |
| Zhu, 2005[[93](file:///C:\Users\Administrator\Desktop\PLOSone回编辑tou\正文\Traditional%20Chinese%20Medicine%20for%20Neck%20Pain%20and%20Low%20Back%20Pain1.docx#_ENREF_93)] | Y | N | Y | N | N | N | Y | N | Y | Y | Y | N | 6 | Fair |

ITT indicates intention to treat; RCTs, randomized clinical trials; Y, yes; N, no; ?, unsure. If the percentage of withdrawals and drop-outs does not exceed 20% for short-term follow-up and 30% for long-term follow-up and does not lead to substantial bias a “yes” is scored. (N.B. these percentages are arbitrary, not supported by literature).

$ study was unpublished.
