# Supplementary material for: Traditional Chinese Medicine for Neck Pain and Low Back Pain: A Systematic Review and Meta-Analysis
Source: PLoS One. 2015 Feb 24;10(2):e0117146. doi: 10.1371/journal.pone.0117146 (PMC4339195; doi:10.1371/journal.pone.0117146)
Supplement: S8 Table — (DOC) [file pone.0117146.s009.doc]

**S8 Table. Strength of Evidence and Clinical significance (Major Outcomes: Pain Intensity, Disability).**

| **Number of Studies and Participants** | **Outcome ( Follow-Up Time)** | **Comparison** | **Type of Evidence** | **Domains Pertaining to Strength of Evidence** | | | | **Effect Size (95% CI)** | **Clinical**  **Difference** | **Strength of Evidence** |
| --- | --- | --- | --- | --- | --- | --- | --- | --- | --- | --- |
|  |  |  |  | **Quality** | **Consistency** | **Directness** | **Preciseness** |  |  |  |
| **Acupuncture in NP** | | | | | | | | | | |
| **7, 428** | Pain (immediate) | Acupuncture v sham-acupuncture | RCT | Fair | Y | Y | Y | -0.58 [-0.94, -0.22] MD | Small | Moderate |
| **2,290** | Pain (1 month) |  | RCT | Fair | Y | Y | Y | -0.72 [-1.07, -0.37] MD | Small | Moderate |
| **3,319** | Pain (short term) |  | RCT | Fair | Y | Y | N | -0.32 [-0.68, 0.04] MD | No | Low |
| **4, 334** | Disability (immediate) |  | RCT | Fair | Y | Y | Y | -0.29 [-0.51, -0.07] SMD | Small | Moderate |
| **2,290** | Disability (1 month) |  | RCT | Fair | Y | Y | Y | -0.42 [-0.66, -0.19] SMD | Small | Moderate |
| **3, 305** | Disability (short term) |  | RCT | Fair | Y | Y | Y | -0.37 [-0.59, -0.14] SMD | Small | Moderate |
| **3, 272** | Pain (immediate) | Acupuncture v sham TENS | RCT | Fair | Y | Y | N | 0.73 [-2.05, 3.51] MD | No | Low |
|  | Pain (short term) |  | RCT | Fair | Y | Y | N | 0.45 [-0.98, 1.87] MD | No | Low |
|  | Disability (immediate) |  | RCT | Fair | Y | Y | N | 0.40 [-0.55, 1.36] SMD | No | Low |
|  | Disability (short term) |  | RCT | Fair | Y | Y | N | -0.18 [-0.51, 0.15] SMD | No | Low |
| **1, 108** | Pain (immediate) | Acupuncture v sham laser | RCT | Fair | NA | Y | N | -0.69 [-1.75, 0.37] MD | No | Low |
| **1, 108** | Pain (immediate) | Acupuncture v massage | RCT | Fair | NA | Y | Y | -1.63 [-2.68, -0.58] MD | Medium | Moderate |
| **4, 146** | Pain (immediate) | Acupuncture v medication | RCT | Fair | N | Y | N | -0.57 [-1.14, -0.01] SMD | Medium | Low |
| **2, 94** | Disability(immediate) |  | RCT | Fair | Y | Y | N | -0.18 [-0.59, 0.23] SMD | No | Low |
| 2, 99 | Pain (immediate) | Acupuncture v manipulation | RCT | Fair | Y | Y | N | -0.08 [-0.49, 0.32] SMD | No | Low |
| 1, 100 | Pain (short term) |  | RCT | Fair | Y | Y | N | 0.01 [-0.38, 0.40] SMD | No | Low |
| 2, 99 | Disability(immediate) |  | RCT | Fair | Y | Y | N | 0.49 [0.08, 0.89] SMD | No | Low |
| 1, 120 | Pain (immediate) | Acupuncture v traction | RCT | Fair | Y | Y | N | 1.31 [0.78, 1.84] MD | No | Low |
| 1, 30 | Pain (immediate) | Acupuncture v waitlist | RCT | Fair | NA | Y | Y | 26 [3.686, 183.418] OR | Large | Low |
| **Acupuncture in LBP** | | | | | | | | | | |
| **9, 1387** | Pain (immediate) | Acupuncture v sham-acupuncture | RCT | Fair | N | Y | Y | -0.49 [-0.76, -0.21] SMD | Small | Low |
| **6, 1261** | Pain (short term) |  | RCT | Fair | N | Y | Y | -0.45 [-0.76, -0.14] SMD | Small | Low |
| **3, 1054** | Pain (intermediate term) |  | RCT | Fair | Y | Y | Y | -0.15 [-0.27, -0.03] SMD | Small | Moderate |
| **5, 1536** | Disability (immediate term) |  | RCT | Fair | N | Y | Y | -0.15 [-0.46, 0.16] SMD | Small | Low |
| **3, 1436** | Disability (short term) |  | RCT | Fair | N | Y | Y | 0.07 [-0.10, 0.23] SMD | Small | Low |
| **4, 1525** | Disability (intermediate term) |  | RCT | Fair | N | Y | Y | -0.02 [-0.24, 0.20] SMD | Small | Low |
| **4, 2911** | Pain (immediate) | Acupuncture v waitlist | RCT | Fair | N | Y | Y | -0.73 [-0.96, -0.49] SMD | Medium | Low |
| **3, 451** | Disability (immediate) |  | RCT | Fair | N | Y | Y | -0.95 [-1.42, -0.48] SMD | Large | Low |
| **2, 70** | Pain (immediate term) | Acupuncture v TENS | RCT | Fair | N | Y | Y | **0.46 [-3.16, 4.08] MD** | No | Low |
| **2, 70** | Pain (short term) |  | RCT | Fair | Y | Y | Y | **-1.02 [-3.08, 1.04] MD** | No | Moderate |
| 6, 242 | Pain (immediate) | Acupuncture v medications | RCT | Fair | Y | Y | Y | -0.52 [-1.27, 0.23] MD | No | Moderate |
| 4, 186 | Disability (immediate) |  | RCT | Fair | Y | Y | Y | -0.23 [-0.52, 0.06] SMD | Small | Moderate |
| **6, 443** | Pain (immediate) | Acupuncture v usual care | RCT | Fair | N | Y | Y | -1.56 [-2.45, -0.67] SMD | Large | Low |
| **5, 383** | Pain (follow-up) |  | RCT | Fair | N | Y | Y | -1.76 [-2.76, -0.75] SMD | Large | Low |
| **5, 320** | Pain (immediate) | Acupuncture + usual care v usual care | RCT | Fair | N | Y | Y | **-11.47 (-19.33, -3.61)** MD | Medium | Low |
| **5, 320** | Pain (follow-up) |  | RCT | Fair | N | Y | Y | **-14.30 (-26.07, -2.54)** MD | Medium | Low |
| **4, 195** | Disability (immediate) |  | RCT | Fair | N | Y | N | **-0.45 (-1.18, 0.29)** SMD | No | Low |
| **4, 195** | Disability (follow-up) |  | RCT | Fair | Y | Y | Y | **-0.55 (-1.00, -0.10)** SMD | Medium | Moderate |
| **Acupuncture in LBP (Acute or subacute)** | | | | | | | | | | |
| **3, 188** | Pain (immediate) | Acupuncture v sham-acupuncture | RCT | Fair | Y | Y | Y | -9.38 (-17.00, -1.76) MD | Small | Moderate |
| **Acupressure in NP** | | | | | | | | | | |
| **1,32** | Pain (1 week) | Acupressure + CT v conventional treatment (CT) | RCT | Fair | NA | Y | Y | 23% reduction in VAS, P= 0.02, effect size =0.43) | ? | Low |
|  | Pain ( 1 month) |  | RCT | Fair | NA | Y | Y | 23% reduction in VAS, P= 0.02, effect size =0.43) | ? | Low |
| **Acupressure in LBP** | | | | | | | | | | |
| **2,275** | Pain (immediate) | Acupressure v Physical therapy | RCT | Fair | Y | Y | Y | -0.73 [-0.97, -0.48] SMD | Medium | Moderate |
|  | Pain (intermediate) |  | RCT | Fair | N | Y | Y | -0.95 [-1.39, -0.51] SMD | Large | Low |
| **1,129** | Disability (immediate) |  | RCT | Fair | NA | Y | Y | -3.8 (-5.7, -1.9) MD | Medium | Moderate |
|  | Disability (intermediate) |  | RCT | Fair | NA | Y | Y | -4.5 (-6.1, -2.9) MD | Medium | Moderate |
| **1,21** | Pain (immediate) | Acupressure( auricular) v Sham | RCT | Fair | N | Y | N | -1.36 [-2.93, 0.21] SMD | No | Low |
|  | Pain (short term) |  | RCT | Fair | Y | Y | N | -0.36 [-0.98, 0.27] SMD | No | Low |
| **1,21** | Disability (immediate) |  | RCT | Fair | NA | Y | Y | -5.33 (-9.81, -0.85) MD | Large | Low |
|  | Disability (short term) |  | RCT | Fair | NA | Y | Y | -4.23 (-7.83, -0.63) MD | Medium | Low |
| **1,51** | Pain (immediate) | Acupressure + CT v conventional treatment (CT) | RCT | Fair | NA | Y | Y | -0.38 (-0.41, -0.35) MD | Small | Low |
|  | Disability (immediate) |  | RCT | Fair | NA | Y | Y | -0.12 (-0.14, -0.10) MD | Small | Low |
| **Cupping in NP** | | | | | | | | | | |
| **2, 93** | Pain (immediate) | Cupping v wait list | RCT | Fair | Y | Y | Y | -19.10 (-27.61, -10.58) MD | Medium | Moderate |
|  | Disability (immediate) |  | RCT | Fair | Y | Y | Y | -6.65 (-10.97, -2.32) MD | Small | Moderate |
| **1, 48** | Pain (immediate) | Cupping v standard medical care | RCT | Fair | NA | Y | Y | -1.72 (-2.74, -0.70) MD | Medium | Low |
|  | Disability (immediate) |  | RCT | Fair | NA | Y | Y | -5.78 (-10.80, -0.76) MD | Small | Low |
| **1, 40** | Pain (1 week) | Cupping v heating pad | RCT | Fair | NA | Y | Y | -36.30 (-46.48, -26.12) MD | Large | Low |
|  | Pain (1 month) |  | RCT | Fair | NA | Y | Y | -21.55 [-34.92, -8.18] MD | Large | Low |
|  | Disability (1 week) |  | RCT | Fair | NA | Y | Y | -7.69 [-13.68, -1.70] MD | Small | Low |
|  | Disability (1 month) |  | RCT | Fair | NA | Y | Y | -10.44 [-15.48, -5.40] MD | Medium | Low |
| **1, 61** | Pain (immediate) | Cupping v Progressive muscle relaxation | RCT | Fair | NA | Y | N | -0.16 (-13.90, 13.55) MD | No | Low |
|  | Disability (immediate) |  | RCT | Fair | NA | Y | Y | -2.18 (-4.56, -0.21) MD | Small | Low |
| **Cupping in LBP** | | | | | | | | | | |
| **4, 430** | Pain (immediate term) | Cupping v medications | RCT | Poor | N | Y | Y | -0.54 [-0.89, -0.19] MD | Small | Low |
| **3, 180** |  | Cupping (retention) v medications | RCT | Poor | Y | Y | N | -0.04 [-0.23, 0.15] MD | No | Low |
| **2, 120** |  | Cupping (balance) v medications | RCT | Poor | Y | Y | Y | -0.65 [-0.81, -0.48] MD | Small | Low |
| **1, 60** |  | Cupping (wet) v medications | RCT | Poor | NA | Y | N | -1.10 [-1.68, -0.52] MD | Medium | Low |
| **1, 70** |  | Cupping (moving) v medications | RCT | Poor | NA | Y | N | -2.28 [-3.42, -1.14] MD | Large | Low |
| **3, 360** | Disability (immediate term) | Cupping v medications | RCT | Poor | N | Y | Y | -3.77 [-5.85, -1.69] MD | Small | Low |
| **3, 180** |  | Cupping (retention) v medications | RCT | Poor | Y | Y | Y | -1.41 [-2.67, -0.16] MD | Small | Low |
| **2, 120** |  | Cupping (balance) v medications | RCT | Poor | Y | Y | Y | -6.06 [-7.54, -4.57] MD | Medium | Low |
| **1, 60** |  | Cupping (wet) v medications | RCT | Poor | NA | Y | N | -5.90 [-7.57, -1.69] MD | Medium | Low |
| **1, 98** | Pain (Short term) | Cupping v usual care | RCT | Fair | NA | Y | Y | -2.20 (-2.60, -1.70) MD | Large | Moderate |
|  | Disability (Short term) |  | RCT | Fair | NA | Y | Y | -15.0 (-18.8, -11.2) MD | Large | Moderate |
| **1, 32** | Pain (immediate) | Cupping v wait list | RCT | Fair | NA | Y | N | -6.9 (-19.16, 5.36) MD | No | Low |
|  | Pain (2 weeks) |  | RCT | Fair | NA | Y | N | -0.8 (-12.16, 10.56) MD | No | Low |
|  | Disability (immediate) |  | RCT | Fair | NA | Y | N | -3.8 (-8.98, 1.38) MD | No | Low |
|  | Disability (2 weeks) |  | RCT | Fair | NA | Y | N | -2.4 (-8.48, 3.68) MD | No | Low |
| **Gua sha in NP** | | | | | | | | | | |
| **1,48** | Pain (immediate ) | Gua sha v thermal therapy | RCT | Fair | NA | Y | Y | -29.9 (-43.3, -16.6) MD | Large | Low |
|  | Disability (immediate ) |  | RCT | Fair | NA | Y | Y | -8.5 (-13.6, -3.5) MD | Small | Low |
| **1,21** | Pain (immediate) | Gua sha v wait list | RCT | Fair | NA | Y | Y | -1.6 (-3.0, -0.1) MD | Medium | Low |
|  | Disability (immediate ) |  | - | - | - | - | - | - | - | Insufficient |
| **Gua sha in LBP** | | | | | | | | | | |
| **1,19** | Pain (immediate ) | Gua sha v wait list | RCT | Fair | NA | Y | Y | -1.1 (-2.0, -0.2) MD | Medium | Low |
|  | Disability (immediate) |  | - | - | - | - | - | - | - | Insufficient |
| **Qigong in NP** | | | | | | | | | | |
| **2,158** | Pain (short) | Qigong v Exercise | RCT | Fair | Y | Y | N | 1.88 (-5.77, 9.54) MD | No | Low |
|  | Disability (short) |  | RCT | Fair | Y | Y | N | 1.29 (-4.32, 6.91)) MD | No | Low |
|  | Pain (intermediate) |  | RCT | Fair | Y | Y | N | 1 (-6.21, 8.21) MD | No | Low |
|  | Disability (intermediate) |  | RCT | Fair | Y | Y | N | 0.02 (-5.25, 5.28) MD | No | Low |
| **2,161** | Pain (short) | Qigong v Waitlist | RCT | Fair | Y | Y | Y | -15.27 [-22.49,-8.05] MD | Medium | Moderate |
|  | Disability (short) |  | RCT | Fair | Y | Y | Y | -7.67 [-12.45, -2.88] MD | Small | Moderate |
|  | Pain (intermediate) |  | RCT | Fair | Y | Y | Y | -10.18 [-16.63, -3.73] MD | Medium | Moderate |
|  | Disability (intermediate) |  | RCT | Fair | Y | Y | N | 0.43 [-4.43, 5.28] MD | No | Low |
| **Qigong in LBP** | | | | | | | | | | |
| **0,0** | Pain or Disability | - | - | - | - | - | - | - | - | Insufficient |
| **Tai chi in NP** | | | | | | | | | | |
| **0,0** | Pain or Disability | - | - | - | - | - | - | - | - | Insufficient |
| **Tai chi in LBP** | | | | | | | | | | |
| **1, 160** | Pain (immediate) | Tai chi v Waitlist | RCT | Fair | NA | Y | Y | -1.3 (-0.7, -1.9) MD | Medium | Moderate |
| **1, 160** | Disability (immediate) |  | RCT | Fair | NA | Y | Y | -5.7 (-1.8,- 9.6) MD | Small | Moderate |
| **Chinese herbal medicine in NP** | | | | | | | | | | |
| **1,360** | Pain (immediate) | Exractum nucis vomicae v Diclofenac diethylamine emulgel | RCT | Fair | NA | Y | Y | -0.27 [-0.31, -0.23] MD | Small | Moderate |
| **1,240$** | Pain (short term) | Qishe vs. placebo | RCT | Fair | NA | Y | Y | -0.67 [-0.82, -0.52] MD | Large | Moderate |
| **1,440$** | Pain (short term) | Qishe plus placebo Jingfukang v placebo Qishe plus Jingfukang | RCT | Fair | NA | Y | N | -0.08 [-0.18, 0.02] MD | No | Low |
| **0,0** | Disability | - | - | - | - | - | - | - | - | Insufficient |
| **Chinese herbal medicine in LBP** | | | | | | | | | | |
| **0,0** | Pain or Disability | - | - | - | - | - | - | - | - | Insufficient |
| **Chinese manipulation (CM) in NP** | | | | | | | | | | |
| 2, 183 | Pain (Immediate) | CM v Chinese massage | RCT | Fair | Y | Y | Y | -2.00 [-2.55, -1.45] MD | Large | Moderate |
| 1, 63 | Pain (Short term) |  | RCT | Fair | NA | Y | Y | –2.47 (–3.42, –1.52) MD | Large | Low |
| 1, 63 | Disability (Immediate) |  | RCT | Fair | NA | Y | Y | –9.35 (–14.06, –4.64) MD | Large | Low |
|  | Disability (Short term) |  | RCT | Fair | NA | Y | Y | –10.81 (–15.78, –5.84) MD | Large | Low |
| 1, 213 | Pain (Immediate) | CM v cervical traction | RCT | Fair | NA | Y | Y | –1.06 ( –1.37, –0.75) MD | Medium | Moderate |
| **Chinese manipulation in LBP** | | | | | | | | | | |
| **0,0** | Pain or Disability | - | - | - | - | - | - | - | - | Insufficient |
| **Moxibustion in NP or LBP** | | | | | | | | | | |
| **0,0** | Pain or Disability | - | - | - | - | - | - | - | - | Insufficient |
| **Tuina in NP or LBP** | | | | | | | | | | |
| **0,0** | Pain or Disability | - | - | - | - | - | - | - | - | Insufficient |

CI = confidence interval; MD = mean difference; SMD = standard mean difference; RCT = randomized controlled trial; LBP = low back pain; NP = neck pain; NA = not applicable; Y = yes; N = no; － = no evidence; ? = unclear; NSCLBP = non-specific chronic low back pain; OR = odds ratio.

$, study was unpublished.
